# Supplementary material for: Discovery of extended product structural space of the fungal dioxygenase AsqJ
Source: Nat Commun. 2023 Jun 20;14:3658. doi: 10.1038/s41467-023-39111-2 (PMC10282068; doi:10.1038/s41467-023-39111-2)
Supplement: Supplementary file 1 — Supplementary information [file 41467_2023_39111_MOESM1_ESM.docx]

**Discovery of extended product structural space of the fungal dioxygenase AsqJ**

Manuel Einsiedler^1^ and Tobias A. M. Gulder^1,2^*

^1^ Chair of Technical Biochemistry, Faculty of Chemistry and Food Chemistry, Technische Universität Dresden, Bergstraße 66, 01069 Dresden, Germany; *E-mail: tobias.gulder@tu-dresden.de.

^2^ Helmholtz Institute for Pharmaceutical Research Saarland (HIPS), Department of Natural Product Biotechnology, Helmholtz Centre for Infection Research (HZI) and Department of Pharmacy at Saarland University, Campus E8.1, 66123, Saarbrücken, Germany.

**Supplementary Information**

Contents

[**1. General Methods** 2](#_Toc134014418)

[**1.1 Chemical Methods** 2](#_Toc134014419)

[**1.2 Biochemical/Molecular Biological Methods** 4](#_Toc134014420)

[**2. Chemical Procedures** 6](#_Toc134014421)

[**2.1 Precursor Synthesis** 6](#_Toc134014422)

[**2.1.1 Liquid Phase Synthesis of Fmoc-*N*-methyl amino acids** 6](#_Toc134014423)

[**2.1.2 Protection of Functionalized Acids** 8](#_Toc134014424)

[**2.1.3 Linear Precursor Synthesis** 15](#_Toc134014425)

[**2.1.3.1 By SPPS** 15](#_Toc134014426)

[**2.1.3.2 By Liquid Phase Synthesis** 19](#_Toc134014427)

[**2.2 Substrate Synthesis** 23](#_Toc134014428)

[**2.2.1 By Direct Cyclization from Resin** 23](#_Toc134014429)

[**2.2.2 By Acid-induced Cyclization** 39](#_Toc134014430)

[**2.2.3 By Final *N^1^*-Methylation** 40](#_Toc134014431)

[**2.2.4 By Macrocyclization** 41](#_Toc134014432)

[**2.3 Synthesis of Standards** 45](#_Toc134014433)

[**3. Enzymatic Assays** 49](#_Toc134014434)

[**3.1 Procedure** 49](#_Toc134014435)

[**3.2 HPLC Data of Qualitative Assays** 49](#_Toc134014436)

[**3.3 Upscaled Assays for Product Structure Elucidation** 71](#_Toc134014437)

[**3.4 Quantification of Substrate Consumption** 81](#_Toc134014438)

[**4. Additional Schemes** 88](#_Toc134014439)

[**5. Selected UV Spectra** 91](#_Toc134014440)

[**6. NMR Data** 94](#_Toc134014441)

[**7. Supplementary References** 182](#_Toc134014442)

**1. General Methods**

**1.1 Chemical Methods**

**Solvents and reagents**

Solvents for HPLC and MS analysis, such as acetonitrile and methanol, were purchased from Fisher Scientific and VWR in a purity of over 99% (HPLC-grade). Water was purified using a TKA GenPure water treatment system and deionized. Dry solvents, such as acetonitrile, dichloromethane, methanol, ethanol and tetrahydrofuran, for procedures under inert atmosphere were prepared by distillation and dried over molecular sieves (3 Å or 4 Å). Commercial materials and other solvents were purchased at the highest commercial quality from the providers Acros Organics, Alfa Aesar, Carbolution, Carl Roth, Merck, Sigma Aldrich, VWR, TCI Chemicals and Thermo Fisher Scientific. H_2_^18^O (97% IE) was purchased from Deutero.

**Solid phase peptide synthesis (SPPS)**

*Fmoc test*

A small amount of resin (5 − 10 mg) was deprotected in 1 mL of 20% piperidine in DMF for 20 minutes. The resin was filtered off and 100 μL of the obtained solution was diluted with 10 mL of DMF. Three samples of 1 mL were transferred into cuvettes and the absorbance was measured using a NanoPhotometer® P330 by *Implen*, while DMF was used as reference. The absorbances were used to calculate the average resin loading via:

$$resin loading \left[ \frac{mmol}{g} \right]=\frac{101\cdot A_{301nm}}{7.8\cdot m_{resin}}$$

With $A_{301nm}$: Absorbance at 301 nm and $m_{resin}$: mass of used loaded resin in mg.

*Test cleavage*

A few milligrams of loaded resin were treated with 0.5 mL 5% trifluoroacetic acid in DCM for 10 minutes. The resin was filtered off and the filtrate was concentrated using an air stream. The obtained residue was diluted in 40 μL H_2_O and 80 μL ACN, before being analysed using HPLC.

**Chromatography**

Thin-layer chromatography (TLC) was performed on precoated plates of silica gel F254 (Merck) with UV detection at 254 and 365 nm. Column chromatography was performed on silica gel 60 Geduran® Si 60 (40-60 μm) (Merck). High Performance Liquid Chromatography (HPLC) analysis was performed on a Jasco system consisting of an UV-1575 Intelligent UV/VIS-Detector, DG-2080-53 3-Line Degasser, two PU-1580 Intelligent HPLC Pumps, AS-1550 Intelligent Sampler, HG-1580-32 Dynamic Mixer and a LC-NetII/ADC. The system was controlled by the Galaxie software. A reversed phase column (Eurospher II, 100-3 C18 A, 150 × 4.6 mm) with integrated precolumn manufactured by Knauer was used at 25 °C with the following solvents: A = H_2_O + 0.05% TFA, B = ACN + 0.05% TFA. The separation method consisted of the following gradient system: 0–2 min: 95% A, 2–25 min: 95–5% A, 25–28 min: 5% A, 28–31 min: 95% A, with a flowrate of 1 mL/min. All runs were monitored at 220 nm. Purification of compounds was performed on a semi-preparative Medium Pressure Liquid Chromatography (MPLC) device (Reveleris X2) manufactured by Grace with reversed phase columns (C18, 12 g/4 g). The system was controlled by the Reveleris Navigator software and the eluent system consisted of A and B. The separation method used the following gradient system: 0–2 min: 95% A, 2–21 min: 95–5% A, 21–23 min: 5% A, 23–25 min: 95% A, with a flowrate of 28 mL/min or 12 mL/min. Preparative HPLC was performed on a Jasco system consisting of an UV-1575 Intelligent UV/VIS-Detector, two PU-2086 Plus Intelligent Prep pumps, MIKA 1000 Dynamic Mixing Chamber, 1000 µL injection port and a LC-NetII/ADC. The system was controlled by the Galaxie software. A reversed phase column (100-5 C18 A, 250 × 16 mm) manufactured by Knauer was used. The separation method consisted of the following gradient system: 0–2 min: 95% A, 2–25 min: 95–5% A, 25–26 min: 5% A, 26–28 min: 95% A. The used flowrate was 12 mL/min at 220 nm.

**LC-MS**

Liquid Chromatography, coupled to Mass Spectrometry (LC-MS), was performed on an Azura HPLC device manufactured by Knauer, consisting of the following components: AS 6.1L sampler, P 6.1L pump, DAD 2.1L detector. This device was coupled to an ESI mass spectrometer manufactured by Advion with single-quadrupole mass analyser. The system was controlled by ClarityChrom software in combination with Mass Express software. The same gradient as described above on a C‑18 column (Eurospher II, 100-3 C18 A, 150 × 4.6 mm) was used.

**HR-MS**

For high resolution mass spectrometry (HR-MS) a LTQ FT Ultra with ESI and linear ion trap manufactured by Thermo Fisher Scientific, as well as an Agilent mass spectrometer 6538 with ESI or atmospheric pressure chemical ionization (APCI), with high resolution Q-TOF mass analyser and microchannel plate detector were used.

**NMR**

^1^H and ^13^C Nuclear Magnetic Resonance spectra (NMR) were recorded on Bruker AVANCE 300 and AVANCE 600 spectrometers at room temperature. The chemical shifts are given in δ-values (ppm) downfield from TMS and are referenced on the residual peak of the deuterated solvent (Ac-d_6_: δ_H_ = 2.05 ppm, δ_C_ = 29.9 ppm; CDCl_3_: δ_H_ = 7.26 ppm, δ_C_ = 77.2 ppm; DMSO-d_6_: δ_H_ = 2.50 ppm, δ_C_ = 39.5 ppm; MeOD-d_4_: δ_H_ = 3.31 ppm, δ_C_ = 49.0 ppm). ^19^F spectra are referenced on TFA (Ac-d_6_: δ_F_ = –76.87 ppm; CDCl_3_: δ_F_ = –75.39 ppm; DMSO-d_6_: δ_F_ = –74.95 ppm)^1^ or by unified chemical shift scale. The coupling constants *J* are given in Hertz [Hz]. The following abbreviations were used for the allocation of signal multiplicities: bs – broad singlet, s – singlet, d – doublet, bd – broad doublet, dd – doublet of doublets, ddd – doublet of doublets of doublets, t – triplet, dt – doublet of triplets, q – quartet, dq – doublet of quartets, h – heptet, m – multiplet. In case of different conformers with a predominant one, only the ^13^C signals of the major conformer are given (see chapter 6 for complete data). Spectra of some of the highly diluted samples have been baseline corrected by MestReNova software. ^13^C and ^19^F spectra were measured with proton decoupling (^13^C {^1^H} and ^19^F {^1^H}).

**Specific rotation**

The specific rotation was measured on a PerkinElmer Model 341 LLC or a Krüss P3000 polarimeter at room temperature in methanol or chloroform. The concentration of the compounds during the measurements is given in 10 mg/mL.

**1.2 Biochemical/Molecular Biological Methods**

Recombinant AsqJ was produced similar to a procedure described in literature as follows:^2^

**Bacterial culture**

The recombinant *E. coli* strain BL21(DE3) containing the plasmid pET28bSUMOAnAsqJ^3^ was grown in Erlenmeyer flasks containing 1 L of terrific broth (TB) medium supplemented with 1 mL kanamycin solution (50 mg/mL). The cultures were incubated at 37 °C with shaking (180 rpm) to an OD_600_ of about 0.8. 100 µL of isopropylthiogalactoside (IPTG) solution (1 M) was added and incubation was continued for ca. 13 h with shaking at 18 °C. Cells were harvested by centrifugation (15300 × *g*, 10 min), washed with 0.9% (w/v) NaCl, and stored as pellets (16800 × *g*, 10 min) in falcon tubes at –80 °C.

**Protein purification**

Frozen bacterial cell mass (15 g) was thawed in 90 mL of 50 mM sodiumdihydrogenphosphate (NaH_2_PO_4_), pH 8.0, containing 300 mM NaCl, 10 mM imidazole and 10% (v/v) glycerol. The cells were disrupted by sonication (Bandelin Sonopuls HD2070) with an amplitude of 35% (10 s pulse, 10 s pause). The resulting suspension was centrifuged at 16800 × *g* for 30 min at 4 °C using a Thermo Fisher Scientific Heraeus Multifuge X3R Centrifuge. 2 mL of nickel NTA resin suspension were added to the supernatant, followed by incubation at 0 °C for 1 h with slight shaking. The resin was applied on a HisPur NTA column from Thermo Fisher Scientific.

For **p**urification, two different methods were applied:

Method P1) Washing with 20 mL of 50 mM NaH_2_PO_4_, pH 7.5, containing 300 mM NaCl and 20 mM imidazole (washing buffer) and elution with 5 mL of 50 mM NaH_2_PO_4_, pH 8.0, containing 300 mM NaCl and 250 mM imidazole (elution buffer).

Method P2) Applying a stepwise gradient of washing and elution buffer to the following concentrations: 0–25 min: 20–135 mm imidazole; 25–35 min: 135 mm imidazole; 35–45 min: 135–235 mm imidazole. AsqJ eluted at imidazole concentration of ~135 mm.

**D**esalting and concentration were performed with two different methods:

Method D1) Using Sephadex G-25 PD-10 columns from GE Healthcare: The columns were equilibrated with 20 mM TRIS, pH 7.4, containing 100 mM NaCl, 100 µM FeSO_4_, and 4 mM ascorbic acid (buffer A). After applying 2.5 mL of enzyme solution, the protein was eluted with 3.5 mL of buffer A. The enzyme was concentrated using VivaSpin centrifugal filters from Sartorius Stedim Biotech (exclusion size: 10000 MWCO, 4200 × *g*).

Method D2) The eluted enzyme was directly applied to the VivaSpin filter, treated with 30 mL of buffer A, and concentrated as described above. This procedure was repeated three times.

Concentration of the solution was determined photometrically using a NanoPhotometer P330 from Implen (E = 29450, M = 47343.88 Da). The complete purification procedure was performed at 0 °C. The purified enzyme was stored in aliquots at –80 °C.

The yield of purified enzyme was between 8 and 11 mg / L main culture.

An example of a SDS PAGE gel of AsqJ expression/purification is given below.


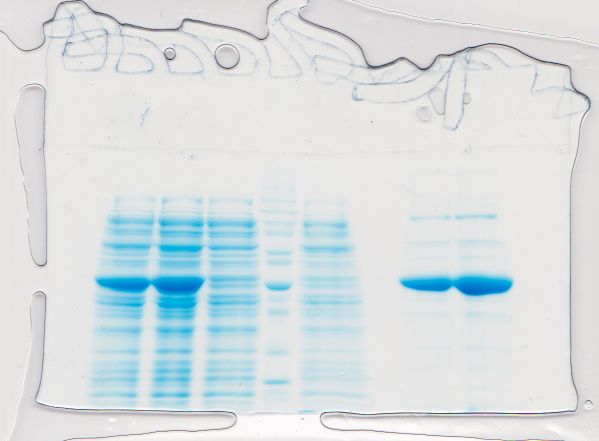


50

25

60

70

85

---------S---------

F

M

W

---------E---------

30 µg

20 µg

20 µg

10 µg

3.2 µg

6.4 µg

AsqJ, 47.3 kDa

**Supplementary Figure 1.** Example of SDS PAGE of AsqJ purification (employing methods P2 and D2) with amounts of applied samples. S – supernatant after sonication, F – flowthrough of Ni-NTA column, M – marker (protein ladder sizes are given in kDa), W – washing fraction of Ni-NTA column, E – eluted and concentrated enzyme.

# **2. Chemical Procedures**

## **2.1 Precursor Synthesis**

### **2.1.1 Liquid Phase Synthesis of Fmoc-*N*-methyl amino acids**

In the following section, the individual substitution of the precursor molecules **12**, **13**, **11** is defined as **a**: R = *^i^*Bu and **b**: R = benzyl.

**Oxazolidinone Formation^4^**

In a flame-dried 250 mL-Schlenk flask, the respective *N*-Fmoc amino acid (1.0 eq.) was dissolved in dry DCM (9 mL/mmol), and paraformaldehyde (10 eq.) and magnesium sulfate (8 g) were added. The mixture was vigorously stirred and BF_3_·OEt_2_ (1.0 eq.) was added slowly. After stirring under argon for 3 hours, the mixture was filtered and the solids were rinsed with DCM. The combined filtrates were washed with 1 m HCl_aq_ (1 × 25 mL) and brine (1 × 25 mL), dried over Na_2_SO_4_ and the solvent removed under reduced pressure. The crude product was purified by column chromatography (pentane/EtOAc = 10/1 to 5/1) to yield the oxazolidinone.

(9*H*-fluoren-9-yl)methyl (*S*)-4-isobutyl-5-oxooxazolidine-3-carboxylate (**13a**)

This compound was synthesized from *N*-Fmoc-l-leucine, yielding 2.75 g of a colourless oil, containing residual EtOAc from purification (10%, calculated from ^1^H NMR), resulting in a yield of 2.49 g (6.81 mmol, 80%).

**^1^H-NMR** (600 MHz, CDCl_3_)**:** δ [ppm] = 7.79–7.75 (m, 2 H), 7.57–7.52 (m, 2 H), 7.43–7.39 (m, 2 H), 7.33 (dt, *J* = 7.4, 1.1 Hz, 2 H), 5.56–5.17 (bs, 1 H), 5.07 (bd, *J* = 4.7 Hz, 1 H), 4.80–4.49 (bs, 2 H), 4.23 (t, *J* = 5.5 Hz, 1 H), 1.83–1.51 (bs, 2 H), 1.50–1.28 (bs, 1 H), 1.03–0.64 (bs, 6 H).

**^13^C-NMR** (151 MHz, CDCl_3_)**:** δ [ppm] = 172.6, 153.2, 143.5 (2C), 141.5 (2C), 128.1 (2C), 127.3 (2C), 124.6 (2C), 120.2 (2C), 77.4*, 67.3, 53.5, 47.3, 39.2, 24.3, 22.7, 22.2.

* Signal overlaps with solvent peak.

NMR spectra showed partly broad signals and a mixture of conformers. Only the ^13^C signals of the major conformer are given.

**HR-MS** (APCI+)**:** *m/z* calcd. for C_22_H_23_NO_4_ [M+H]^+^: 366.1700, found: 366.1697.

**HPLC:** *t*_R_ = 15.3 min.

***R*_f_:** 0.26 (pentane/EtOAc = 10/1).

**Specific rotation:** [α]_D_ = +78.4 °·mL·dm^–1^·g^–1^ (ρ = 1.60; MeOH).

(9*H*-fluoren-9-yl)methyl (*S*)-4-benzyl-5-oxooxazolidine-3-carboxylate (**13b**)

This compound was synthesized from *N*-Fmoc-l-phenylalanine, yielding 3.15 g of a colourless oil, containing residual EtOAc from purification (6%, calculated from ^1^H NMR), resulting in a yield of 2.49 g (7.46 mmol, 83%).

**^1^H-NMR** (300 MHz, CDCl_3_)**:** δ [ppm] = 7.80–7.75 (m, 2 H), 7.69–7.31 (m, 6 H), 7.30–7.04 (m, 4 H), 6.71 (bs, 1 H), 5.18–4.92 (m, 1.7 H), 4.75–4.67 (m, 1 H), 4.51 (bd, 0.8 H), 4.27 (t, *J* = 5.1 Hz, 1 H), 4.15 (bs, 0.3 H), 4.02 (bd, 1 H), 3.48–3.11 (m, 0.8 H), 2.78 (bd, *J* = 13.9 Hz, 0.5 H), 2.41 (bd, *J*= 13.6 Hz, 0.5 H).

**^13^C-NMR** (75 MHz, CDCl_3_)**:** δ [ppm] = 172.0, 152.4, 143.5 (2C), 141.6 (2C), 134.3, 129.6 (2C), 128.8 (2C), 128.1 (2C), 127.6, 127.4 (2C), 124.6 (2C), 120.3 (2C), 78.0, 66.8, 56.4, 47.4, 35.2.

NMR spectra showed partly broad signals and a mixture of conformers. Only the ^13^C signals of the major conformer are given.

**HR-MS** (APCI+)**:** *m/z* calcd. for C_25_H_21_NO_4_ [M+H]^+^: 400.1543, found: 400.1538.

**HPLC:** *t*_R_ = 15.4 min.

***R*_f_:** 0.17 (pentane/EtOAc = 7/1).

**Specific rotation:** [α]_D_ = +137.0 °·mL·dm^–1^·g^–1^ (ρ = 2.25; MeOH).

**Reductive Oxazolidinone Cleavage**

In a 250 mL round-bottomed flask, the corresponding 5-oxooxazolidinone (1.0 eq.) was dissolved in DCM and TFA (1/1, 6 mL/mmol), triethylsilane (3.0 eq.) was added slowly and the resulting mixture stirred at room temperature for 18 hours. The volatiles were removed under reduced pressure and the residue purified by MPLC to yield the respective Fmoc-protected *N*-methylated amino acid.

*N*-(((9*H*-fluoren-9-yl)methoxy)carbonyl)-*N*-methyl-l-leucine (**11a**)

This compound was synthesized from (9*H*-fluoren-9-yl)methyl (*S*)-4-isobutyl-5-oxooxazolidine-3-carboxylate, yielding 2.1 g of a white solid (5.71 mmol, 84%).

**^1^H-NMR** (300 MHz, CDCl_3_)**:** δ [ppm] = 11.19 (bs, 1 H), 7.73–7.65 (m, 2 H), 7.59–7.49 (m, 2 H), 7.37–7.19 (m, 4 H), 4.94 (dd, *J* = 8.7, 7.2 Hz, 0.6 H), 4.56 (dd, *J* = 10.6, 5.9 Hz, 0.4 H), 4.50–4.38 (m, 2 H), 4.23 (t, *J* = 6.9 Hz, 0.6 H), 4.16 (t, *J* = 6.9 Hz, 0.4 H), 2.83 (s, 1.7 H), 2.83 (s, 1.3 H), 1.75–1.70 (m, 1.2 H), 1.64–1.38 (m, 1.8 H), 0.92 (dd, *J* = 8.3, 6.5 Hz, 3.7 H), 0.84 (d, *J* = 6.6 Hz, 1.2 H), 0.71 (d, *J* = 6.5 Hz, 1.1 H).

**^13^C-NMR** (75 MHz, CDCl_3_)**:** δ [ppm] = 177.5, 157.2, 143.8 (2C), 141.3 (2C), 127.7 (2C), 127.1 (2C), 125.0 (2C), 120.0 (2C), 67.8, 56.6, 47.2, 37.2, 30.3, 24.8, 23.3, 21.2.

NMR spectra showed a mixture of conformers. Only the ^13^C signals of the major conformer are given.

**HR-MS** (APCI+)**:** *m/z* calcd. for C_22_H_25_NO_4_ [M+H]^+^: 368.1856, found: 368.1854.

**HPLC:** *t*_R_ = 14.6 min.

**Specific rotation:** [α]_D_ = –7.6 °·mL·dm^–1^·g^–1^ (ρ = 4.15; MeOH).

*N*-(((9*H*-fluoren-9-yl)methoxy)carbonyl)-*N*-methyl-l-phenylalanine (**11b**)

This compound was synthesized from (9*H*-fluoren-9-yl)methyl (*S*)-4-benzyl-5-oxooxazolidine-3-carboxylate, yielding 2.66 g of a white solid (6.62 mmol, 89%).

**^1^H-NMR** (300 MHz, CDCl_3_)**:** δ [ppm] = 11.39 (s, 1 H), 7.85–7.77 (m, 2 H), 7.60–7.27 (m, 10.2 H), 7.04 (d, *J* = 7.1 Hz, 0.8 H), 5.09 (dd, *J* = 11.1, 5.0 Hz, 0.6 H), 4.71 (dd, *J* = 10.8, 4.9 Hz, 0.4 H), 4.62 (dd, *J* = 10.7, 5.8 Hz, 0.4 H), 4.49–4.37 (m, 1.6 H), 4.26 (t, *J* = 6.7 Hz, 0.6 H), 4.20 (t, *J* = 6.1 Hz, 0.4 H), 3.50 (dd, *J* = 14.6, 5.0 Hz, 0.6 H), 3.27–3.16 (m, 1 H), 2.89 (s, 1.7 H), 2.87 (s, 1.3 H), 2.83 (dd, *J* = 14.5, 10.9 Hz, 0.4 H).

**^13^C-NMR** (75 MHz, CDCl_3_)**:** δ [ppm] = 176.0, 156.8, 143.9 (2C), 141.2 (2C), 136.9, 128.8 (2C), 128.6 (2C), 127.7 (2C), 127.1 (2C), 126.9, 125.0 (2C), 120.0 (2C), 67.9, 60.6, 47.0, 34.6, 32.3.

NMR spectra showed a mixture of conformers. Only the ^13^C signals of the major conformer are given.

**HR-MS** (APCI+)**:** *m/z* calcd. for C_25_H_23_NO_4_ [M+H]^+^: 402.1700, found: 402.1697.

**HPLC:** *t*_R_ = 14.5 min.

**Specific rotation:** [α]_D_ = –48.1 °·mL·dm^–1^·g^–1^ (ρ = 4.60; MeOH).

### **2.1.2 Protection of Functionalized Acids**

The corresponding anthranilic acid (1.0 eq.) was dissolved in a mixture of H_2_O/dioxane (1/1, 20 mL/mmol), then sodium carbonate (2.5 eq.) was added. After complete solvation of the base, Fmoc chloride (1.2 eq.) was added in small portions over 10 minutes and the resulting mixture stirred at room temperature for 18 hours. The solution was diluted with 15 mL of H_2_O, before being extracted with Et_2_O (2 × 20 mL). The aqueous phase was acidified with 1 m HCl_aq_ and extracted with EtOAc (3 × 20 mL). The combined organic phases were dried over Na_2_SO_4_, filtered, and the solvents removed under reduced pressure to yield the *N*-Fmoc-protected acid, which was used without further purification, if not stated otherwise.

2-((((9*H*-fluoren-9-yl)methoxy)carbonyl)amino)-6-fluorobenzoic acid (**16a**)

This compound was synthesized starting from 2-amino-6-fluorobenzoic acid, yielding 231 mg of an orange solid, containing residual (11%, calculated from ^1^H NMR) dioxane, resulting in a yield of 208 mg (0.55 mmol, 85%).

**^1^H-NMR** (300 MHz, Ac-d_6_)**:** δ [ppm] = 10.34 (bs, 1 H), 8.13 (bd, *J* = 9.0 Hz, 1 H), 7.88 (dt, *J* = 7.5, 1.1 Hz, 1 H), 7.73–7.69 (m, 2 H), 7.57 (td, *J* = 8.5, 6.0 Hz, 1 H), 7.46–7.39 (m, 2 H), 7.34 (td, *J* = 7.4, 1.3 Hz, 2 H), 6.93 (ddd, *J* = 11.1, 8.3, 1,1 Hz, 1 H), 4.51 (d, *J* = 7.4 Hz, 1 H), 4.35 (t, *J* = 7.0 Hz, 1 H).

**^13^C-NMR** (75 MHz, Ac-d_6_)**:** δ [ppm] = 168.2 (d, *J* = 2.7 Hz), 163.2 (d, *J* = 256 Hz), 153.8, 144.8 (2C), 143.0 (d, *J* = 4.2 Hz), 142.2 (2C), 135.3 (d, *J* = 11.0 Hz), 128.7 (2C), 128.0 (2C), 126.0 (2C), 120.9 (2C), 115.8 (d, *J* = 3.6 Hz), 110.9 (d, *J* = 23.5 Hz), 107.5 (d, *J* = 14.6 Hz), 67.8, 47.8.

**^19^F-NMR** (282 MHz, Ac-d_6_)**:** δ [ppm] = –107.26 (bs, 1 F).

**HR-MS** (ESI+)**:** *m/z* calcd. for C_22_H_16_FNO_4_ [M+Na]^+^: 400.0956, found: 400.0959.

**HPLC:** *t*_R_ = 15.0 min.

2-((((9*H*-fluoren-9-yl)methoxy)carbonyl)amino)-5-fluorobenzoic acid (**16b**)

This compound was synthesized starting from 2-amino-5-fluorobenzoic acid, yielding 234 mg of a light yellow solid, containing residual (2%, calculated from ^1^H NMR) dioxane, resulting in a yield of 230 mg (0.61 mmol, 84%).

**^1^H-NMR** (300 MHz, Ac-d_6_)**:** δ [ppm] = 10.69 (bs, 1 H), 8.42 (dd, *J* = 9.2, 5.0 Hz, 1 H), 7.88 (dt, *J* = 7.5, 1.0 Hz, 2 H), 7.78 (dd, *J* = 9.3, 3.2 Hz, 1 H), 7.74–7.69 (m, 2 H), 7.46–7.30 (m, 5 H), 4.51 (d, *J* = 7.0 Hz, 2 H), 4.36 (t, *J* = 7.0 Hz, 1 H).

**^13^C-NMR** (75 MHz, Ac-d_6_)**:** δ [ppm] = 169.2 (d, *J* = 2.4 Hz), 157.7 (d, *J* = 240 Hz), 154.0, 144.8 (2C), 142.2 (2C), 139.4 (d, *J* = 2.4 Hz), 128.7 (2C), 128.0 (2C), 126.0 (2C), 122.4 (d, *J* = 22.4 Hz), 121.4 (d, *J* = 7.2 Hz), 120.9 (2C), 117.9 (d, *J* = 24.1 Hz), 116.9 (d, *J* = 6.9 Hz), 67.7, 47.8.

**^19^F-NMR** (282 MHz, Ac-d_6_)**:** δ [ppm] = –123.19 (bs, 1 F).

**HR-MS** (ESI+)**:** *m/z* calcd. for C_22_H_16_FNO_4_ [M+Na]^+^: 400.0956, found: 400.0954.

**HPLC:** *t*_R_ = 15.6 min.

2-((((9*H*-fluoren-9-yl)methoxy)carbonyl)amino)-4-fluorobenzoic acid (**16c**)

This compound was synthesized starting from 2-amino-4-fluorobenzoic acid, yielding 233 mg of a white solid (0.62 mmol, 89%).

**^1^H-NMR** (300 MHz, Ac-d_6_)**:** δ [ppm] = 11.05 (bs, 1 H), 8.25–8.13 (m, 2 H), 7.89 (dt, *J* = 7.6, 1.0 Hz, 2 H), 7.75–7.71 (m, 2 H), 7.46–7.40 (m, 2 H), 7.35 (dt, *J* = 7.4, 1.3 Hz, 2 H), 6.91 (ddd, *J* = 8.9, 7.9, 2.7 Hz, 1 H), 4.54 (d, *J* = 7.0 Hz, 2 H), 4.38 (t, *J* = 7.0 Hz, 1 H).

**^13^C-NMR** (75 MHz, Ac-d_6_)**:** δ [ppm] = 169.7 (d, *J* = 5.4 Hz), 167.1 (d, *J* = 251 Hz), 153.9, 144.8 (2C), 142.2 (2C), 135.2 (d, *J* = 10.7 Hz), 128.7 (2C), 128.1 (2C), 126.0 (2C), 121.0 (2C), 112.0 (d, *J* = 3.0 Hz), 109.7 (d, *J* = 22.4 Hz), 106.2 (d, *J* = 3.3 Hz), 105.8 (d, *J* = 3.3 Hz), 67.9, 47.8.

**^19^F-NMR** (282 MHz, Ac-d_6_)**:** δ [ppm] = –104.56 (s), –104.58 (s).

**HR-MS** (ESI+)**:** *m/z* calcd. for C_22_H_16_FNO_4_ [M+Na]^+^: 400.0956, found: 400.0955.

**HPLC:** *t*_R_ = 15.8 min.

2-((((9*H*-fluoren-9-yl)methoxy)carbonyl)amino)-3-fluorobenzoic acid (**16d**)

This compound was synthesized starting from 2-amino-3-fluorobenzoic acid, yielding 207 mg of an orange solid, containing residual (8%, calculated from ^1^H NMR) starting material, resulting in a yield of 191 mg (0.51 mmol, 84%). The crude product was used without further purification. For spectroscopic analysis, a small amount of sample was purified by MPLC.

**^1^H-NMR** (600 MHz, Ac-d_6_)**:** δ [ppm] = 8.86 (bs, 1 H), 7.87 (dt, *J* = 7.6, 0.9 Hz, 2 H), 7.83 (dt, *J* = 7.9, 1.3 Hz, 1 H), 7.72 (bd, *J* = 7.6 Hz, 2 H), 7.47 (ddd, *J* = 10.3, 8.3, 1.5 Hz, 1 H), 7.42 (7.5, 0.9 Hz, 2 H), 7.38 (td, *J* = 8.1, 5.0 Hz, 1 H), 7.33 (td, *J* = 7.5, 1.2 Hz, 1 H), 4.41 (d, *J* = 7.4 Hz, 2 H), 4.31 (t, *J* = 7.4 Hz, 1 H).

**^13^C-NMR** (151 MHz, Ac-d_6_)**:** δ [ppm] = 167.7 (d, *J* = 4.0 Hz), 157.8 (d, *J* = 249 Hz), 154.4, 144.9 (2C), 142.1 (2C), 128.6 (2C), 128.1 (d, *J* = 13.9 Hz), 128.0 (2C), 127.24 (d, *J* = 7.6 Hz), 127.19 (d, *J* = 2.9 Hz), 126.9 (d, *J* = 8.1 Hz), 126.2 (2C), 121.0 (d, *J* = 20.8 Hz), 120.9 (2C), 68.0, 47.8.

**^19^F-NMR** (282 MHz, Ac-d_6_)**:** δ [ppm] = –120.59 (bs, 1 F).

**HR-MS** (ESI+)**:** *m/z* calcd. for C_22_H_16_FNO_4_ [M+Na]^+^: 400.0956, found: 400.0960.

**HPLC:** *t*_R_ = 13.9 min.

2-((((9*H*-fluoren-9-yl)methoxy)carbonyl)amino)-6-chlorobenzoic acid (**16e**)

This compound was synthesized starting from 2-amino-6-chlorobenzoic acid, yielding 540 mg of an orange solid, containing residual (11%, calculated from ^1^H NMR) dioxane, resulting in a yield of 480 mg (1.22 mmol, 83%).

**^1^H-NMR** (300 MHz, Ac-d_6_)**:** δ [ppm] = 8.87 (bs, 1 H), 7.93 (d, *J* = 8.1 Hz, 1 H), 7.85 (dt, *J* = 7.6, 1.1 Hz, 2 H), 7.73–7.65 (m,  H), 7.46–7.37 (m, 3 H), 7.32 (dt, *J* = 7.4, 1.3 Hz, 2 H), 7.25 (dd, *J* = 8.1, 1.1 Hz, 1 H), 4.48 (d, *J* = 7.2 Hz, 2 H), 4.30 (t, *J* = 7.0 Hz, 1 H).

**^13^C-NMR** (151 MHz, Ac-d_6_)**:** δ [ppm] = 167.3, 154.2, 144.8 (2C), 142.1 (2C), 139.2, 132.8, 132.5, 128.6 (2C), 128.0 (2C), 126.0 (2C), 125.9, 124.2, 121.2, 120.9 (2C), 67.8, 47.8.

**HR-MS** (ESI+)**:** *m/z* calcd. for C_22_H_16_ClNO_4_ [M+Na]^+^: 416.0660, 418.0631; found: 416.0664, 418.0644.

**HPLC:** *t*_R_ = 14.4 min.

2-((((9*H*-fluoren-9-yl)methoxy)carbonyl)amino)-5-chlorobenzoic acid (**16f**)

This compound was synthesized starting from 2-amino-5-chlorobenzoic acid, yielding 132 mg of a light yellow solid (0.34 mmol, 59%).

**^1^H-NMR** (300 MHz, Ac-d_6_)**:** δ [ppm] = 10.78 (bs, 1 H), 8.41 (d, *J* = 9.0 Hz, 1 H), 8.05 (d, *J* = 2.6 Hz, 1 H), 7.89 (dt, *J* = 7.6, 1.0 Hz, 2 H), 7.75–7.69 (m, 2 H), 7.62 (dd, *J* = 9.1, 2.6 Hz, 2 H), 7.46–7.39 (m, 2 H), 7.35 (dt, *J* = 7.5, 1.3 Hz, 2 H), 4.53 (d, *J* = 6.9 Hz, 2 H), 4.36 (t, *J* = 6.9 Hz, 1 H).

**^13^C-NMR** (75 MHz, DMSO-d_6_)**:** δ [ppm] = 169.1, 153.8, 144.8 (2C), 142.2 (2C), 141.8, 135.1, 131.5, 128.7 (2C), 128.0 (2C), 127.0, 126.0 (2C), 121.1, 121.0 (2C), 117.0, 67.8, 47.8.

**HR-MS** (ESI+)**:** *m/z* calcd. for C_22_H_16_ClNO_4_ [M+Na]^+^: 416.0660, 418.0631; found: 416.0663, 418.0640.

**HPLC:** *t*_R_ = 16.1 min.

2-((((9*H*-fluoren-9-yl)methoxy)carbonyl)amino)-4-chlorobenzoic acid (**16g**)

This compound was synthesized starting from 2-amino-4-chlorobenzoic acid, yielding 450 mg of a white solid (1.14 mmol, 78%).

**^1^H-NMR** (300 MHz, DMSO-d_6_)**:** δ [ppm] = 13.93 (bs, 1 H), 10.89 (s, 1 H), 8.26 (d, *J* = 2.1 Hz, 1 H), 7.97 (d, *J* = 8.6 Hz, 1 H), 7.91 (dt, *J* = 7.7, 0.9 Hz, 2 H), 7.71–7.65 (m, 2 H), 7.46–7.39 (m, 2 H), 7.34 (td, *J* = 7.4, 1.3 Hz, 2 H), 7.17 (dd, *J* = 8.6, 2.1 Hz, 1 H), 4.51 (d, *J* = 6.8 Hz, 2 H), 4.36 (t, *J* = 6.7 Hz, 1 H).

**^13^C-NMR** (75 MHz, DMSO-d_6_)**:** δ [ppm] = 169.0, 152.6, 143.5 (2C), 142.0, 140.8 (2C), 138.8, 133.0, 127.8 (2C), 127.2 (2C), 125.0 (2C), 122.0, 120.3 (2C), 117.8, 114.5, 66.5, 46.4.

**HR-MS** (ESI+)**:** *m/z* calcd. for C_22_H_16_ClNO_4_ [M+Na]^+^: 416.0660, 418.0631; found: 416.0662, 418.0638.

**HPLC:** *t*_R_ = 16.3 min.

2-((((9*H*-fluoren-9-yl)methoxy)carbonyl)amino)-4-nitrobenzoic acid (**16h**)

This compound was synthesized starting from 2-amino-4-nitrobenzoic acid and purified by MPLC, yielding 270 mg of a yellow solid (0.67 mmol, 49%).

**^1^H-NMR** (300 MHz, Ac-d_6_)**:** δ [ppm] = 10.94 (bs, 1 H), 9.32 (d, *J* = 2.3 Hz, 1 H), 8.34 (d, *J* = 8.7 Hz, 1 H), 7.92 (dd, *J* = 8.8, 2.3 Hz, 1 H), 7.88 (dt, *J* = 7.6, 1.0 Hz, 2 H), 7.76–7.71 (m, 2 H), 7.46–7.39 (m, 2 H), 7.34 (dt, *J* = 7.5, 1.0 Hz, 2 H), 4.57 (d, *J* = 7.0 Hz, 2 H), 4.38 (t, *J* = 7.0 Hz, 1 H).

**^13^C-NMR** (75 MHz, Ac-d_6_)**:** δ [ppm] = 169.0, 153.8, 152.2, 144.7 (2C), 143.8, 142.2 (2C), 133.8, 128.7 (2C), 128.1 (2C), 126.0 (2C), 121.0 (2C), 120.3, 116.7, 113.9, 68.1, 47.8.

**HR-MS** (ESI+)**:** *m/z* calcd. for C_22_H_16_N_2_O_6_ [M+Na]^+^: 427.0901, found: 427.0903.

**HPLC:** *t*_R_ = 15.8 min.

2-((((9*H*-fluoren-9-yl)methoxy)carbonyl)amino)-6-methylbenzoic acid (**16i**)

This compound was synthesized starting from 2-amino-6-methylbenzoic acid, yielding 630 mg of a slightly orange solid, containing residual (11%, calculated from ^1^H NMR) dioxane, resulting in a yield of 559 mg (1.49 mmol, 91%).

**^1^H-NMR** (300 MHz, Ac-d_6_)**:** δ [ppm] = 9.52 (bs, 1 H), 7.98 (d, *J* = 7.1 Hz, 1 H), 7.87 (dt, *J* = 7.6, 1.0 Hz, 2 H), 7.73–7.69 (m, 2 H), 7.45–7.40 (m, 2 H), 7.39–7.32 (m, 3 H), 7.02 (dt, *J* = 7.6, 1.0 Hz, 1 H), 4.47 (d, *J* = 7.1 Hz, 2 H), 4.33 (t, *J* = 7.1 Hz, 1 H), 2.52 (s, 3 H).

**^13^C-NMR** (75 MHz, Ac-d_6_)**:** δ [ppm] = 170.4, 154.1, 144.9 (2C), 142.1 (2C), 140.0, 139.9, 132.4, 128.6 (2C), 128.0 (2C), 126.7, 126.0 (2C), 121.2, 120.9 (2C), 119.0, 67.5, 47.8, 22.6.

^13^C-NMR showed a mixture of conformers. Only the signals of the major isomer are given.

**HR-MS** (ESI+)**:** *m/z* calcd. for C_23_H_19_NO_4_ [M+Na]^+^: 396.1206, found: 396.1208.

**HPLC:** *t*_R_ = 14.8 min.

2-((((9*H*-fluoren-9-yl)methoxy)carbonyl)amino)-5-methylbenzoic acid (**16j**)

This compound was synthesized starting from 2-amino-5-methylbenzoic acid, yielding 470 mg of a white solid, containing residual (1%, calculated from ^1^H NMR) dioxane, resulting in a yield of 464 mg (1.24 mmol, 75%).

**^1^H-NMR** (300 MHz, DMSO-d_6_)**:** δ [ppm] = 13.61 (bs, 1 H), 10.66 (s, 1 H), 8.04 (d, *J* = 8.5 Hz, 1 H), 7.90 (dt, *J* = 7.5, 1.0 Hz, 2 H), 7.78 (d, *J* = 2.4 Hz, 1 H), 7.70–7.65 (m, 2 H), 7.46–7.30 (m, 5 H), 4.47 (d, *J* = 6.8 Hz, 2 H), 4.34 (t, *J* = 6.8 Hz, 1 H).

**^13^C-NMR** (75 MHz, DMSO-d_6_)**:** δ [ppm] = 169.7, 152.7, 143.7 (2C), 140.8 (2C), 138.4, 134.9, 131.2, 131.1, 127.7 (2C), 127.2 (2C), 125.0 (2C), 120.2 (2C), 118.5, 115.8, 66.2, 46.5, 20.1.

**HR-MS** (ESI+)**:** *m/z* calcd. for C_23_H_19_NO_4_ [M+Na]^+^: 396.1206, found: 396.1209.

**HPLC:** *t*_R_ = 15.9 min.

2-((((9*H*-fluoren-9-yl)methoxy)carbonyl)amino)-3-methylbenzoic acid (**16k**)

This compound was synthesized starting from 2-amino-3-methylbenzoic acid and purified by MPLC, yielding 360 mg of a slightly red solid (0.96 mmol, 58%).

**^1^H-NMR** (300 MHz, Ac-d_6_)**:** δ [ppm] = 8.86 (bs, 1 H), 7.90–7.82 (m, 3 H), 7.71 (bs, 2 H), 7.49 (bd, *J* = 7.6 Hz, 1 H), 7.41 (bt, *J* = 7.5 Hz, 2 H), 7.32 (bt, *J* = 7.5 Hz, 2 H), 7.26 (bt, *J* = 7.7 Hz, 1 H), 4.41 (d, *J* = 7.3 Hz, 2 H), 4.30 (bt, *J* = 7.4 Hz, 1 H), 2.31 (s, 3 H).

**^13^C-NMR** (151 MHz, Ac-d_6_)**:** δ [ppm] = 168.9, 154.8, 144.9 (2C), 142.1 (2C), 138.1, 136.7, 135.9, 129.4, 128.6 (2C), 128.0 (2C), 126.22 (2C), 126.19, 120.8 (2C), 67.5, 48.0, 18.8.

^13^C-NMR partly showed a mixture of conformers. Only the signals of the major isomer are given. One quaternary carbon could not be detected.

**HR-MS** (ESI+)**:** *m/z* calcd. for C_23_H_19_NO_4_ [M+Na]^+^: 396.1206, found: 396.1207.

**HPLC:** *t*_R_ = 14.2 min.

3-((((9H-fluoren-9-yl)methoxy)carbonyl)amino)-2-naphthoic acid (**16l**)

This compound was synthesized starting from 3-amino-2-naphthoic acid, yielding 440 mg of a yellow solid (1.07 mmol, 80%).

**^1^H-NMR** (300 MHz, DMSO-d_6_)**:** δ [ppm] = 13.92 (bs, 1 H), 10.79 (s, 1 H), 8.70 (s, 1 H), 8.56 (bs, 1 H), 8.01 (bd, *J* = 8.0 Hz, 1 H), 7.92 (dt, *J*= 7.6, 1.0 Hz, 2 H), 7.84 (d, *J* = 8.2 Hz, 1 H), 7.73–7.69 (m, 2 H), 7.60 (ddd, *J* = 8.2, 6.8, 1.3 Hz, 1 H), 7.48–7.41 (m, 3 H), 7.35 (dt, *J* = 7.4, 1.2 Hz, 2 H), 4.52 (d, *J* = 6.9 Hz, 2 H), 4.38 (t, *J* = 6.9 Hz, 1 H).

**^13^C-NMR** (151 MHz, DMSO-d_6_)**:** δ [ppm] = 169.7, 152.9, 143.7 (2C), 140.8 (2C), 136.1, 135.6, 133.3, 129.4, 129.1, 127.9, 127.8 (2C), 127.2 (2C), 126.9, 125.3, 125.1 (2C), 120.3 (2C), 117.0, 115.0, 66.3, 46.5.

**HR-MS** (ESI+)**:** *m/z* calcd. for C_26_H_19_NO_4_ [M+Na]^+^: 432.1206, found: 432.1203.

**HPLC:** *t*_R_ = 16.5 min.

(2*S*,4*R*)-1-(((9*H*-fluoren-9-yl)methoxy)carbonyl)-4-hydroxypyrrolidine-2-carboxylic acid (**SI-1**)

In a 100 mL round-bottomed flask, 0.50 g *trans*-l-4-hydroxyproline (3.81 mmol, 1.0 eq.) was dissolved in a mixture of H_2_O and dioxane (14 mL/14 mL), and 1.01 g sodium carbonate (9.53 mmol, 2.5 eq.) was added. After cooling to 0 °C, 1.18 g Fmoc chloride (4.57 mmol, 1.2 eq.) was added in small portions over 15 minutes and the mixture stirred at room temperature for 18 hours. The slurry was diluted with water (20 mL) and extracted with Et_2_O (2 × 20 mL). The aqueous extracts were acidified by addition of 1 m HCl_aq_ to a pH of around 2, before being extracted with EtOAc (3 × 30 mL). Combined organic extracts were washed with brine (20 mL), dried over Na_2_SO_4_, filtered and the volatiles removed under reduced pressure, yielding 1.54 g of a white solid, containing residual (14%, calculated from ^1^H NMR), resulting in a yield of 1.32 g (3.74 mmol, 98%).

**^1^H-NMR** (600 MHz, Ac-d_6_)**:** δ [ppm] = 11.17 (bs, 1 H), 7.89–7.83 (m, 2 H), 7.78–7.69 (m, 2 H), 7.44–7.38 (m, 2 H), 7.36–7.29 (m, 2 H), 4.60 (t, *J* = 7.9 Hz, 0.4 H), 4.53 (bp, *J* = 4.4 Hz, 0.6 H), 4.50 (bp, *J* = 4.4 Hz, 0.4 H), 4.43 (t, *J*= 7.9 Hz, 0.6 H), 4.34–4.28 (m, 2 H), 4.24–4.18 (m, 1 H), 3.70 (dd, *J* = 10.9, 4.4 Hz, 0.6 H), 3.62–3.59 (m, overlap with signal of residual dioxane), 2.44–2.39 (m, 0.4 H), 2.35–2.29 (m, 0.6 H), 2.19 (ddd, *J* = 12.8, 7.6, 4.8 Hz, 0.4 H), 2.15–2.10 (ddd, *J* = 12.8, 7.6, 4.8 Hz, 0.6 H).

**^13^C-NMR** (151 MHz, Ac-d_6_)**:** δ [ppm] = 174.4, 173.8, 155.5, 155.2, 145.2, 145.1, 145.0, 144.9, 142.09, 142.07, 142.03, 141.97, 128.6, 128.5, 128.00, 127.97, 126.3, 126.2, 126.1, 120.84, 120.83, 120.79, 120.77, 70.3, 69.5, 68.4, 68.0, 58.8, 58.4, 56.0, 55.5, 48.0, 47.9, 40.3, 39.1.

NMR spectra showed a mixture of conformers. All ^13^C signals are given; signals between 145.2 ppm and 120.77 ppm represent two carbons each.

Known compound, CAS 88050-17-3.

(2*S*,4*R*)-1-(((9*H*-fluoren-9-yl)methoxy)carbonyl)-4-((*tert*-butyldimethylsilyl)oxy)pyrrolidine-2-carboxylic acid (**68**)

In a flame-dried 100 mL Schlenk flask, 309 mg Fmoc-*trans*-l-4-Hydroxyproline (874 µmol, 1.0 eq.) was dissolved in DCM (15 mL) and DMF (3 mL), and 343 mg imidazole (5.05 mmol, 5.8 eq.) were added. 303 mg TBSCl (2.02 mmol, 2.31 mmol) were added slowly and the mixture stirred at room temperature for 18 hours. The reaction was quenched by addition of saturated NH_4_Cl solution (20 mL) and the aqueous phase extracted with DCM (20 mL). Combined organic phases were concentrated *in vacuo* and the residue taken up in EtOAc (25 mL). After washing with water (2 × 15 mL), the organic phase was dried over Na_2_SO_4_, filtered and the solvents reduced under reduced pressure. The residue was pre-purified by short column chromatography (DCM to DCM/MeOH = 10/1), which yielded 229 mg of a reddish oil that then was used without further purification. A small amount was purified by preparative HPLC for spectroscopic analysis.

**^1^H-NMR** (600 MHz, CDCl_3_)**:** δ [ppm] = 10.04 (bs, 1 H), 7.76 (bd, *J* = 7.6 Hz, 1.3 H), 7.71 (bd, *J* = 7.5 Hz, 0.7j H), 7.60–7.52 (m, 2 H), 7.40 (bt, *J* = 7.4 Hz, 1.4 H), 7.40 (td, *J* = 7.4, 2.4 Hz, 1.3 H), 7.37–7.32 (m, 0.7 H), 7.32–7.25 (m, 2 H), 4.51 (t, *J* = 7.6 Hz, 0.7 H), 4.48–4.36 (m, 3.3 H), 4.27 (t, *J* = 7.2 Hz, 0.7 H), 4.16 (t, *J* = 7.1 Hz, 0.3 H), 3.66–3.61 (m, 1 H), 3.52 (bd, *J* = 11.0 Hz, 0.3 H), 3.47 (dd, *J* = 10.9, 3.1 Hz, 0.7 H), 2.31–2.25 (m, 0.3 H), 2.21 (dd, *J* = 7.5, 4.7 Hz, 1.3 H), 2.14–2.09 (m, 0.3 H), 0.89 (s, 6 H), 0.87 (s, 3 H), 0.09 (s, 2 H), 0.08 (s, 2 H), 0.07 (s, 1 H), 0.06 (s, 1 H).

**^13^C-NMR** (151 MHz, CDCl_3_)**:** δ [ppm] = 178.1, 176.3, 156.1, 154.8, 144.2, 143.92, 143.90, 143.8, 141.42, 141.40, 141.38, 141.34, 127.88, 127.87, 127.73, 127.71, 127.21, 127.20, 127.18, 125.2, 125.14, 125.09, 120.1, 120.0, 70.4, 69.7, 68.1, 67.8, 58.4, 57.9, 55.4, 55.1, 47.3, 47.2, 40.1, 38.5, 25.8, 18.1, –4.6, –4.71, –4.74.

NMR spectra showed a mixture of conformers. All ^13^C signals are given; signals between 144.2 ppm and 120.0 ppm represent two carbons each.

**HR-MS** (ESI+)**:** *m/z* calcd. for C_26_H_34_NO_5_Si [M+H]^+^: 468.2201, found: 468.2198; calcd. for [M+Na]^+^: 490.2021, found: 490.2018.

### **2.1.3 Linear Precursor Synthesis**

#### **2.1.3.1 By SPPS**

Note: During SPPS of *N^4^*-ethylated substrates (**9m**, **10m**, **33**, **34**), the washing steps after the reactions were conducted with EtOH instead of MeOH.

**Loading of Amino Acids on Wang Resin**

In an SPPS reactor, dry DCM was added to Wang resin (1.1 mmol/g, 1.0 eq.) and shaken for 30 minutes. In a flame-dried Schlenk flask the corresponding amino acid (1.2 eq.) was dissolved in a mixture of DCM and DMF (7:1, 5 mL/mmol acid) and the solution cooled to 0 °C. *N*,*N*’-diisopropylcarbodiimide (DIC, 5.0 eq.) was slowly added, followed by addition of *N*,*N*-dimethylaminopyridine (DMAP, 0.1 eq.). The solution was stirred at 0 °C for 10 minutes, added to the swollen resin, and vigorously shaken for 15 h at room temperature. Afterwards, the solution was removed, and the resin washed with DMF (3 × 5 mL), MeOH (3 × 5 mL) and DCM (4 × 5 mL). After drying the resin *in vacuo*, loading was determined using the Fmoc-test. Loadings were between 0.5 and 0.7 mmol/g.

Capping was achieved by adding a mixture of pyridine (2.0 eq.) and acetic anhydride (2.0 eq.) in 6 mL of DCM to the resin and shaking for 15 minutes. The resin was washed with DCM (4 × 5 mL).

**Fmoc Deprotection**

The resin was washed with DMF (5 mL). Next, the resin was treated with piperidine (20% v/v in DMF) and shaken for 15 minutes. The procedure was repeated once, then the resin was washed with DMF (3 × 5 mL), MeOH/EtOH (3 × 5 mL) and DCM (4 × 5 mL).

***o*-Ns Protection^5^**

The resin was washed with NMP (5 mL). In a 10 mL round bottomed flask *o*-NsCl (4.0 eq.) and collidine (10.0 eq.) were dissolved in 7 mL NMP. This solution was added to the resin and the reaction shaken for 20 minutes. Afterwards, the resin was washed with NMP (3 × 5 mL), MeOH/EtOH (3 × 5 mL) and DCM (4 × 5 mL).

***N*-Alkylation^5^**

The resin was washed with THF (10 mL). In a flame-dried Schlenk flask triphenylphosphine (5.0 eq.) and dry alcohol (10 eq.) was added to THF (8 mL). In another flame-dried flask a solution of diisopropylazodicarboxylate (DIAD, 5.0 eq.) in THF was prepared. These two solutions were added to the resin one after another. Afterwards, the resin was shaken for 30 minutes. The resin was washed with DMF (3 × 5 mL), MeOH (3 × 5 mL) and DCM (4 × 5 mL).

***o*-Ns Deprotection^5^**

The resin was washed with NMP (5 mL). In a 10 mL round bottomed flask mercaptoethanol (10 eq.) and diazabicycloundecen (DBU, 5.0 eq.) were dissolved in NMP (5 mL). After adding this solution to the resin, it was shaken for 10 minutes. The procedure was repeated once, then the resin was washed with NMP (3 × 5 mL), MeOH (3 × 5 mL) and DCM (4 × 5 mL).

**Coupling with (functionalized) Fmoc-protected benzoic acid**

The resin was washed with DMF (5 mL). In a 10 mL round bottomed flask, (functionalized) *N*-Fmoc-benzoic acid (1.3 eq.), HATU (2.0 eq.), HOAt (2.0 eq.) and DIPEA (4.0 eq.) were dissolved in DMF (8 mL). After addition of this solution to the resin, it was shaken for 6 hours. Afterwards, the resin was washed with DMF (3 × 5 mL), MeOH (3 × 5 mL) and DCM (4 × 5 mL).

**Coupling with salicylic acid (86)**

The resin was washed with DMF (5 mL). In a 10 mL round bottomed flask, HATU (1.5 eq.), HOAt (1.5 eq.) and DIPEA (2.0 eq.) were dissolved in DMF (6 mL). This solution was added to the resin and shaken for 5 minutes. Afterwards, salicylic acid (1.3 eq.), dissolved in 2 mL DMF, was added to the mixture and the resin shaken for 45 minutes at room temperature. The resin was washed with DMF (3 × 5 mL), MeOH (3 × 5 mL) and DCM (4 × 5 mL). Success of the reaction was analysed by a test cleavage.

**Coupling with 2-Nitrobenzoic Acid (37)**

The resin was washed with DMF (5 mL). In a 10 mL round bottomed flask 2-nitrobenzoic acid (5.0 eq.), HATU (3.0 eq.), HOAt (3.0 eq.) and DIPEA (4.0 eq.) were dissolved in DMF (8 mL). After addition of this solution to the resin, it was shaken for 3 hours. The procedure was repeated once, then the resin was washed with DMF (3 × 5 mL), MeOH (3 × 5 mL) and DCM (4 × 5 mL).

**Reduction of the Nitro Species**

The resin was washed with DMF (5 mL). In a 10 mL round bottomed flask 5 mL of a solution of SnCl_2_ in DMF was prepared (2M), added to the resin and shaken for 3 hours. The resin was washed with DMF (3 × 5 mL), MeOH (3 × 5 mL) and DCM (4 × 5 mL). Success of the reaction has been analysed by a test cleavage.

**Fmoc Deprotection of Dipeptide**

The second Fmoc deprotection was carried out in the same way as described above.

**Cleavage from Resin**

The resin was treated with 5 mL TFA (35% v/v in DCM) for 15 minutes. The filtrate was collected and the procedure repeated once, after which the resin was washed with DCM. All filtrates were combined and the solvents removed under reduced pressure. The residue was purified by MPLC or preparative HPLC.

*N*-(2-hydroxybenzoyl)-*N*-methyl-*l*-leucine (**88a**)

This compound was synthesized starting from Fmoc-l-leucine, yielding 9.0 mg (36 µmol, 45%) of a white solid.

**^1^H-NMR** (600 MHz, CDCl_3_)**:** δ [ppm] = 7.35 (bt, *J* = 6.4 Hz, 1 H), 7.31 (bd, *J* = 7.5 Hz, 1 H), 7.02 (bd, *J* = 7.6 Hz, 1 H), 6.89 (bt, *J* = 7.3 Hz, ,1 H), 5.08 (bs, 1 H), 3.11 (bs, 3 H), 1.88 (bs, 2 H), 1.65 (bs, 1 H), 1.00 (bs, 6 H).

**^13^C-NMR** (151 MHz, CDCl_3_)**:** δ [ppm] = 176.1, 173.5, 159.9, 133.1, 130.7, 128.4, 119.2, 118.1, 56.5, 37.0, 25.2, 23.3, 21.5.

NMR spectra showed very broad signals.

**HR-MS** (APCI+)**:** *m/z* calcd. for C_14_H_17_NO_3_ [M+H]^+^: 266.1392, found: 266.1386.

**HPLC:** *t*_R_ = 8.8 min.

**Specific rotation:** [α]_D_ = –2.6 °·mL·dm^–1^·g^–1^ (ρ = 1.0; MeOH).

*N*-(2-hydroxybenzoyl)-*N*-methyl-*l*-phenylalanine (**88b**)

This compound was synthesized starting from Fmoc-l-phenylalanine, yielding 11.2 mg of a white solid (37.4 µmol, 29%).

**^1^H-NMR** (600 MHz, CDCl_3_)**:** δ [ppm] = 7.93 (bs, 2 H), 7.36–7.18 (m, 6 H), 6.97 (bd, *J* = 7.5 Hz, 1 H), 6.86 (bs, 1 H), 6.77 (bt, *J* = 7.6 Hz, 1 H), 4.99 (bs, 1 H), 3.47 (bs, 1 H), 3.25 (bs, 1 H), 2.96 (bs, 3 H).

**^13^C-NMR** (151 MHz, CDCl_3_)**:** δ [ppm] = 174.5, 172.9, 157.5, 136.8, 132.8, 129.5, 128.94 (2C), 128.86 (2C), 128.3, 127.3, 119.2, 117.9, 60.6, 37.5, 34.4.

NMR spectra showed very broad signals.

**HR-MS** (APCI+)**:** *m/z* calcd. for C_17_H_17_NO_4_ [M+H]^+^: 300.1236, found: 300.1235.

**HPLC:** *t*_R_ = 9.6 min.

**Specific rotation:** [α]_D_ = –42.6 °·mL·dm^–1^·g^–1^ (ρ = 0.56; MeOH).

#### **2.1.3.2 By Liquid Phase Synthesis**

**Methyl Ester Protection**

In a 100 mL round bottomed Schlenk flask, the corresponding hydroxy acid (1.0 eq.) was dissolved in dry MeOH (10 mL/mmol), after which thionyl chloride (1.3 eq.) was slowly added at 0 °C. The solution was stirred at room temperature for one hour and heated to reflux for 16 hours afterwards. The solvents were removed under reduced pressure and the residue taken up in 70 mL EtOAc. The organic phase was washed with H_2_O (4 × 30 mL) and brine (1 × 30 mL), dried over Na_2_SO_4_, filtered and the solvents removed *in vacuo* to yield the corresponding methyl esters.

Methyl (*S*)-2-hydroxy-4-methylpentanoate (**81a**)

This compound was obtained as slightly yellow oil (0.35 g, 2.4 mmol, 79%).

**^1^H-NMR** (300 MHz, CDCl_3_)**:** δ [ppm] = 4.24–4.16 (m, 1 H), 3.77 (s, 3 H), 2.67 (bs, 1 H), 1.94–1.79 (m, 1 H), 1.61–1.51 (m, 2 H), 0.94 (d, *J* = 6.7, 3 H), 0.93 (d, *J* = 6.6 Hz, 3 H).

Analytical data was in agreement with published data for this compound (CAS 17392-84-6).^6^

Methyl (*S*)-2-hydroxy-3-phenylpropanoate (**81b**)

This compound was obtained as white solid (0.48 g, 2.7 mmol, 89%).

**^1^H-NMR** (300 MHz, CDCl_3_)**:** δ [ppm] = 7.32–7.20 (m, 5 H), 4.47 (dd, *J* = 7.0, 4.4 Hz, 1 H), 3.78 (s, 3 H), 3.13 (dd, *J* = 13.9, 4.3 Hz, 1 H), 2.97 (dd, *J* = 14.0, 6.8 Hz, 1 H).

**HPLC:** *t*_R_ = 6.0 min.

Analytical data was in agreement with published data for this compound (CAS 13673-95-5).^7^

**Esterification**

In a flame dried 25 mL Schlenk flask, 2-nitrobenzoic acid (1.05 eq.) was dissolved in dry THF (15 mL/mmol). DIC and DMAP were added at 0 °C, followed by stirring for 5 minutes. Afterwards, the corresponding alcohol (1. eq.) was added and the mixture allowed to warm to room temperature. After stirring for 18 hours, the solution was poured into 0.1 m aqueous HCl. The aqueous phase was extracted with DCM (2 × 20 mL). Combined organic phases were washed with brine (1 × 30 mL), dried over Na_2_SO_4_, filtered and the solvents removed under reduced pressure. The residue was purified by flash column chromatography with pentane/EtOAc (3/1) to give the ester.

(*S*)-1-methoxy-4-methyl-1-oxopentan-2-yl 2-nitrobenzoate (**82a**)

This compound was synthesized according to the esterification protocol from methyl (*S*)-2-hydroxy-4-methylpentanoate, yielding 80 mg (0.27 mmol, 26%) of a light yellow oil.

**^1^H-NMR** (300 MHz, CDCl_3_)**:** δ [ppm] = 7.94 (dd, *J* = 8.0, 1.3 Hz, 1 H), 7.80 (dd, *J* = 7.6, 1.6 Hz, 1 H), 7.69 (td, *J* = 7.6, 1.3 Hz, 1 H), 7.64 (td, *J* = 7.8, 1.6 Hz, 1 H), 5.30 (dd, *J*= 9.9, 3.9 Hz, 1 H), 3.78 (s, 3 H), 1.90 –1.74 (m, 2 H), 1.73–1.66 (m, 1 H), 0.97 (d, *J* = 6.5 Hz, 3 H), 0.94 (d, *J* = 6.6 Hz, 3 H).

**^13^C-NMR** (75 MHz, CDCl_3_)**:** δ [ppm] = 170.7, 165.2, 147.8, 133.3, 132.0, 130.1, 127.4, 124.1, 72.6, 52.6, 39.8, 24.5, 23.1, 21.4.

**HR-MS** (ESI+)**:** *m/z* calcd. for C_14_H_17_NO_6_ [M+Na]^+^: 318.0948, found: 318.0942.

**HPLC:** *t*_R_ = 13.8 min.

***R*_f_:** 0.36 (pentane/EtOAc = 5/1).

**Specific rotation:** [α]_D_ = +34.5 °·mL·dm^–1^·g^–1^ (ρ = 4.00; MeOH).

(*S*)-1-methoxy-1-oxo-3-phenylpropan-2-yl 2-nitrobenzoate (**82b**)

This compound was synthesized according to the esterification protocol from methyl (*S*)-2-hydroxy-3-phenylpropanoate, yielding 132 mg (0.35 mmol, 62%) of a yellowish oil.

**^1^H-NMR** (300 MHz, CDCl_3_)**:** δ [ppm] = 7.96–7.88 (m, 1 H), 7.74–7.59 (m, 3 H), 7.37–7.22 (m, 5 H), 5.55 (dd, *J* = 7.8, 5.0 Hz, 1 H), 3.79 (s, 3 H), 3.35–3.19 (m, 2 H).

**^13^C-NMR** (75 MHz, CDCl_3_)**:** δ [ppm] = 169.4, 164.7, 148.1, 135.4, 133.0, 132.2, 130.2, 129.4 (2C), 128.6 (2C), 127.3, 126.8, 124.0, 74.5, 52.6, 37.2.

**HR-MS** (ESI+)**:** *m/z* calcd. for C_17_H_15_NO_6_ [M+H]^+^: 352.0792, found: 352.0794.

**HPLC:** *t*_R_ = 13.8 min.

***R*_f_:** 0.51 (pentane/EtOAc = 3/1).

**Specific rotation:** [α]_D_ = +27.8 °·mL·dm^–1^·g^–1^ (ρ = 2.85; MeOH).

**Ester Saponification**

The corresponding ester was dissolved in a mixture of THF/H_2_O (3/2, 25 mL/mmol), and KOH (1.5 eq. was added. The solution was stirred at room temperature for 15 minutes and acidified with HCl_aq_ (1 m) to pH 2. The aqueous phase was extracted with DCM (3 × 15 mL) and the combined organic phases dried over Na_2_SO_4_, filtered and the solvents removed *in vacuo*. The residue was purified by MPLC to yield the corresponding acid.

(*S*)-4-methyl-2-((2-nitrobenzoyl)oxy)pentanoic acid (**83a**)

This compound was synthesized by saponification of (*S*)-1-methoxy-4-methyl-1-oxopentan-2-yl 2-nitrobenzoate, yielding 51 mg of a colourless oil (0.17 mmol, 68% brsm).

**^1^H-NMR** (600 MHz, CDCl_3_)**:** δ [ppm] = 7.97 (dd, *J* = 8.1, 1.3 Hz, 1 H), 7.80 (dd, *J* = 7.6, 1.5 Hz, 1 H), 7.71 (dt, *J* = 7.6, 1.3 Hz, 1 H), 7.66 (dt, *J* = 7.8, 1.5 Hz, 1 H), 5.33 (dd, *J* = 10.1, 3.8 Hz, 1 H), 1.95–1.89 (m, 1 H), 1.88–1.80 (m, 1 H), 1.79–1.73 (m, 1 H), 1.01 (d, *J* = 6.5 Hz, 3 H), 0.98 (d, *J* = 6.6 Hz, 3 H).

**^13^C-NMR** (151 MHz, CDCl_3_)**:** δ [ppm] = 176.4, 165.3, 147.8, 133.4, 132.1, 130.2, 127.3, 124.1, 72.2, 39.7, 24.6, 23.2, 21.4.

**HR-MS** (ESI+)**:** *m/z* calcd. for C_17_H_15_NO_3_ [M+Na]^+^: 304.0792, found: 304.0792.

**HPLC:** *t*_R_ = 12.2 min.

**Specific rotation:** [α]_D_ = +31.7 °·mL·dm^–1^·g^–1^ (ρ = ; MeOH).

(*S*)-2-((2-nitrobenzoyl)oxy)-3-phenylpropanoic acid (**83b**)

This compound was synthesized by saponification of (*S*)-1-methoxy-1-oxo-3-phenylpropan-2-yl 2-nitrobenzoate, yielding 0.11 g of a white solid (0.35 mmol, 90%).

**^1^H-NMR** (300 MHz, CDCl_3_)**:** δ [ppm] = 7.93 (d, *J* = 7.9 Hz, 1 H), 7.71–7.61 (m, 3 H), 7.34–7.22 (m, 5 H), 5.56 (dd, *J* = 8.1, 4.3 Hz, 1 H), 3.34 (dd, *J* = 14.5, 4.3 Hz, 1 H), 3.25 (dd, *J* = 14.5, 8.0 Hz, 1 H).

**^13^C-NMR** (75 MHz, CDCl_3_)**:** δ [ppm] = 173.9, 164.6, 147.9, 135.0, 133.1, 132.1, 130.2, 129.3 (2C), 128.6 (2C), 127.3, 126.6, 124.0, 78.8, 37.0.

**HR-MS** (ESI+)**:** *m/z* calcd. for C_16_H_13_NO_6_ [M+Na]^+^: 338.0635, found: 338.0642.

**HPLC:** *t*_R_ = 12.2 min.

**Specific rotation:** [α]_D_ = +17.0 °·mL·dm^–1^·g^–1^ (ρ = 0.73; MeOH).

**Nitro Reduction**

In a 50 mL Schlenk flask, the respective nitro acid (1.0 eq.) was dissolved in methanol (15 mL/mmol), followed by addition of palladium (10% on activated charcoal, 10 w.-%). The flask was equipped with a hydrogen balloon and flushed three times to generate a hydrogen atmosphere. The mixture was stirred vigorously for 18 hours and filtered afterwards. The solvent was removed under reduced pressure and the residue purified by MPLC to yield the corresponding amine.

(*S*)-2-((2-aminobenzoyl)oxy)-4-methylpentanoic acid (**84a**)

This compound was synthesized by hydrogenation of (*S*)-4-methyl-2-((2-nitrobenzoyl)oxy)pentanoic acid, yielding 20 mg of a slightly orange solid (0.08 mmol, 56%).

**^1^H-NMR** (600 MHz, CDCl_3_)**:** δ [ppm] = 8.47 (bs, 3 H), 7.95 (dd, *J* = 8.0, 1.6 Hz, 1 H), 7.36 (ddd, *J* = 8.5, 7.2, 1.6 Hz, 1 H), 6.91–6.84 (m, 2 H), 5.20 (dd, *J* = 9.9, 3.8 Hz, 1 H), 2.00–1.93 (m, 1 H), 1.91–1.84 (m, 1 H), 1.81–1.74 (m, 1 H), 1.00 (d, *J* = 6.7 Hz, 3 H), 0.97 (d, *J* = 6.6 Hz, 3 H).

**^13^C-NMR** (151 MHz, CDCl_3_)**:** δ [ppm] = 176.2, 167.3, 146.3, 134.9, 131.6, 119.9, 119.1, 113.0, 71.3, 39.8, 25.0, 23.2, 21.7.

**HR-MS** (APCI+)**:** *m/z* calcd. for C_13_H_17_NO_4_ [M+H]^+^: 252.1230, found: 252.1229.

**HPLC:** *t*_R_ = 11.5 min.

**Specific rotation:** [α]_D_ = –29.4 °·mL·dm^–1^·g^–1^ (ρ = 1.0; MeOH).

(*S*)-2-((2-aminobenzoyl)oxy)-3-phenylpropanoic acid (**84b**)

This compound was synthesized by hydrogenation of (*S*)-2-((2-nitrobenzoyl)oxy)-3-phenylpropanoic acid, yielding 54 mg of a white solid (0.19 mmol, 52%).

**^1^H-NMR** (600 MHz, CDCl_3_)**:** δ [ppm] = 7.86 (dd, *J* = 8.1, 1.7 Hz, 1 H), 7.60 (bs, 3 H), 7.32 (bd, *J* = 4.4 Hz, 4 H), 7.29–7.24 (m, 2 H), 6.70–6.64 (m, 2 H), 5.42 (dd, *J* = 8.7, 4.2 Hz, 1 H), 3.36–3.25 (m, 2 H).

**^13^C-NMR** (151 MHz, CDCl_3_)**:** δ [ppm] = 175.7, 167.3, 150.1, 135.9, 134.8, 131.4, 129.5 (2C), 128.7 (2C), 127.3, 117.2, 117.1, 110.3, 72.8, 37.4.

**HR-MS** (APCI+)**:** *m/z* calcd. for C_16_H_15_NO_4_ [M+H]^+^: 286.1074, found: 286.1075.

**HPLC:** *t*_R_ = 11.5 min.

**Specific rotation:** [α]_D_ = –43.1 °·mL·dm^–1^·g^–1^ (ρ = 1.77; MeOH).

## **2.2 Substrate Synthesis**

Loading of acid precursors on the resin and reduction of nitro groups was achieved following the procedures described in chapter 2.1.3.1. Yields are given in relation to loadings determined by the Fmoc test.

### **2.2.1 By Direct Cyclization from Resin**

The resin was dried *in vacuo* and suspended in THF, sodium *tert*-butoxide (2.0 eq.) was added and the mixture was heated to 55 °C under argon atmosphere. After completion of the reaction as monitored by HPLC, the resin was filtered off and washed with methanol (3 × 5 mL). The solvent of the combined filtrates was removed under reduced pressure before being purified as indicated.

(*R*)-3-isobutyl-4-methyl-3,4-dihydro-1*H*-benzo[1,4]diazepine-2,5-dione ((*R*)-**9**)

This compound was synthesized starting from d-Fmoc-leucine, yielding 35 mg of a beige solid (0.14 mmol, 30%).

**^1^H-NMR** (600 MHz, CDCl_3_)**:** δ [ppm] = 9.35 (bs, 0.4 H), 9.15 (bs, 0.6 H), 7.94 (bd, *J* = 8.1 Hz, 1 H), 7.50–7.44 (m, 1 H), 7.28 (t, *J* = 7.9 Hz, 0.6 H), 7.23 (t, *J* = 7.9 Hz, 0.4 H), 7.05 (d, *J* = 8.0 Hz, 0.6 H), 7.01 (d, *J* = 8.1 Hz, 0.4 H), 4.10 (bt, *J* = 7.4 Hz, 0.4 H), 4.01 (bt, *J* = 7.4 Hz, 0.6 H), 3.32 (s, 1.1 H), 3.08 (s, 1.9 H), 1.95–1.89 (m, 0.6 H), 1.82–1.77 (m, 0.8 H), 1.65–1.59 (m, 0.6 H), 1.58–1.52 (m, 0.4 H), 1.38–1.33 (m, 0.6 H), 0.89–0.86 (m, 3.8 H), 0.85 (d, *J* = 6.5 Hz, 1.1 H), 0.81 (d, *J* = 6.5 Hz, 1.1 H).

**^13^C-NMR** (151 MHz, CDCl_3_)**:** δ [ppm] = 170.9, 169.1, 135.8, 123.7, 131.5, 127.4, 125.5, 120.8, 53.7, 34.8, 29.4, 25.0, 22.7, 22.5.

NMR spectra showed a mixture of conformers. Only the ^13^C signals of the major conformer are given.

**HR-MS** (ESI+)**:** *m/z* calcd. for C_14_H_18_N_2_O_2_ [M+H]^+^: 247.1441, found: 247.1446.

**HPLC:** *t*_R_ = 9.2 min.

**LC-MS:** *t*_R_ = 10.7 min.

**Specific rotation:** [α]_D_ = –47.2 °·mL·dm^–1^·g^–1^ (ρ = 1.40; MeOH).

(*S*)-6-fluoro-3-isobutyl-4-methyl-3,4-dihydro-1*H*-benzo[1,4]diazepine-2,5-dione (**9a**)

This compound was obtained after purification by MPLC, yielding 18.0 mg of a white solid (68.1 µmol, 51%).

**^1^H-NMR** (600 MHz, CDCl_3_)**:** δ [ppm] = 9.08 (s, 0.2 H), 8.85 (s, 0.8 H), 7.44–7.37 (m, 1 H), 7.02–6.95 (m, 1 H), 6.85 (dt, *J* = 8.1, 0.9 Hz, 0.8 H), 6.81 (dt, *J* = 8.2, 1.0 Hz, 0.2 H), 4.12 (t, *J* = 7.4 Hz, 0.8 H), 4.06 (t, *J* = 7.4 Hz, 0.2 H), 3.31 (s, 0.6 H), 3.06 (s, 2.4 H), 1.91–1.86 (m, 0.8 H), 1.83–1.78 (m, 0.8 H), 1.67–1.56 (m, 1 H), 1.42–1.33 (m, 0.3 H), 0.90 (dd, *J* = 7.5, 6.6 Hz, 4.8 H), 0.85 (dd, *J* = 8.9, 6.6 Hz, 1.2 H).

**^13^C-NMR** (151 MHz, CDCl_3_)**:** δ [ppm] = 170.8, 164.7, 161.9 (d, *J* = 257 Hz), 137.2 (d, *J* = 4.6 Hz), 132.7 (d, *J* = 10.4 Hz), 116.8 (d, *J* = 15.0 Hz), 116.4 (d, *J* = 3.5 Hz), 113.3 (d, *J* = 22.0 Hz), 53.6, 34.7, 28.6, 24.9, 22.7, 22.5.

**^19^F-NMR** (282 MHz, CDCl_3_)**:** δ [ppm] = –109.28 (s, 0.8 F), –109.36 (s, 0.2 F).

NMR spectra showed a mixture of conformers. Only the ^13^C-signals of the major conformer are given.

**HR-MS** (APCI+)**:** *m/z* calcd. for C_14_H_17_FN_2_O_2_ [M+H]^+^: 299.1190, found: 299.1189.

**HPLC:** *t*_R_ = 9.1 min.

**LC-MS:** *t*_R_ = 10.9 min.

**Specific rotation:** [α]_D_ = +68.9 °·mL·dm^–1^·g^–1^ (ρ = 0.79; MeOH).

(*S*)-7-fluoro-3-isobutyl-4-methyl-3,4-dihydro-1*H*-benzo[1,4]diazepine-2,5-dione (**9b**)

This compound was obtained after purification by preparative HPLC, yielding 6.4 mg of a slightly yellow solid (24.2 µmol, 18%).

**^1^H-NMR** (600 MHz, CDCl_3_)**:** δ [ppm] = 8.04 (bs, 0.3 H), 7.88 (bs, 0.7 H), 7.68–7.62 (m, 1 H), 7.24–7.17 (m, 1 H), 6.97 (dd, *J* = 8.8, 4.5 Hz, 0.7 H), 6.91 (dd, *J* = 8.8, 4.5 Hz, 0.3 H), 4.09 (t, *J* = 7.4 Hz, 0.3 H), 4.00 (t, *J* = 7.4 Hz, 0.7 H), 3.31 (s, 1 H), 3.08 (s, 2 H), 1.97–1.91 (m, 0.7 H), 1.83–1.77 (m, 0.7 H)*, 1.66–1.60 (m, 0.7 H), 1.58–1.54 (m, 0.3 H), 1.40–1.35 (m, 0.7 H), 0.90 (d, *J* = 6.6 Hz, 2 H), 0.89 (d, *J* = 6.6 Hz, 2 H), 0.87 (d, *J* = 6.6 Hz, 1 H), 0.85 (d, *J* = 6.6 Hz, 1 H).

**^13^C-NMR** (151 MHz, CDCl_3_)**:** δ [ppm] = 170.5, 167.6, 159.7 (d, *J* = 247 Hz), 131.7 (d, *J* = 2.9 Hz), 129.1 (d, *J* = 7.5 Hz), 122.5 (d, *J*= 8.1 Hz), 120.1 (d, *J* = 23.1 Hz), 117.6 (d, *J* = 24.3 Hz), 53.5, 34.7, 29.3, 24.9, 22.5, 22.4.

**^19^F-NMR** (282 MHz, CDCl_3_)**:** δ [ppm] = –115.42 (s, 0.7 F), –116.16 (s, 0.3 F).

*Signal overlaps with water peak.

NMR spectra showed a mixture of conformers. Only the ^13^C-signals of the major conformer are given.

**HR-MS** (APCI+)**:** *m/z* calcd. for C_14_H_17_FN_2_O_2_ [M+H]^+^: 265.1347, found: 265.1346.

**HPLC:** *t*_R_ = 9.7 min.

**LC-MS:** *t*_R_ = 11.3 min.

**Specific rotation:** [α]_D_ = +23.5 °·mL·dm^–1^·g^–1^ (ρ = 0.26; MeOH).

(*S*)-8-fluoro-3-isobutyl-4-methyl-3,4-dihydro-1*H*-benzo[1,4]diazepine-2,5-dione (**9c**)

This compound was obtained after purification by MPLC, yielding 9.0 mg of a white solid (34.0 µmol, 26%).

**^1^H-NMR** (600 MHz, CDCl_3_)**:** δ [ppm] = 8.91 (s, 0.4 H), 8.73 (s, 0.6 H), 8.01–7.95 (m, 1 H), 7.00 (td, *J* = 8.1, 2.4 Hz, 0.6 H), 6.95 (td, *J* = 8.3, 2.4 Hz, 0.4 H), 6.75 (dd, *J* = 9.2, 2.4 Hz, 0.6 H), 6.71 (dd, *J* = 9.2, 2.4 Hz, 0.4 H), 4.10 (t, *J* = 7.4 Hz, 0.4 H), 4.01 (t, *J* = 7.4 Hz, 0.6 H), 3.31 (s, 1.1 H), 3.07 (s, 1.9 H), 1.97–1.91 (m, 0.7 H), 1.83–1.77 (m, 0.7 H), 1.66–1.60 (m, 0.6 H), 1.59–1.53 (m, 0.4 H), 1.42–1.36 (m, 0.7 H), 0.90 (d, *J* = 6.7 Hz, 2 H), 0.89 (d, *J* = 6.7 Hz, 2 H), 0.88 (d, *J* = 6.6 Hz, 1 H), 0.86 (d, *J* = 6.6 Hz, 1 H).

**^13^C-NMR** (151 MHz, CDCl_3_)**:** δ [ppm] = 170.5, 168.2, 164.8 (d, *J* = 239 Hz), 137.6 (d, *J* = 10.4 Hz), 134.1 (d, *J* = 10.4 Hz), 123.9 (d, *J* = 2.9 Hz), 113.2 (d, *J* = 21.4 Hz), 107.5 (d, *J* = 24.9 Hz), 53.8, 34.9, 29.4, 25.1, 22.7, 22.6.

**^19^F-NMR** (282 MHz, CDCl_3_)**:** δ [ppm] = –105.71 (s, 0.6 F), –105.79 (s, 0.4 F).

NMR spectra showed a mixture of conformers. Only the ^13^C-signals of the major conformer are given.

**HR-MS** (APCI+)**:** *m/z* calcd. for C_14_H_17_FN_2_O_2_ [M+H]^+^: 265.1347, found: 265.1349.

**HPLC:** *t*_R_ = 9.6 min.

**LC-MS:** *t*_R_ = 11.3 min.

**Specific rotation:** [α]_D_ = +43.3 °·mL·dm^–1^·g^–1^ (ρ = 0.38; MeOH).

(*S*)-9-fluoro-3-isobutyl-4-methyl-3,4-dihydro-1*H*-benzo[1,4]diazepine-2,5-dione (**9d**)

This compound was obtained after purification by preparative HPLC, yielding 12.5 mg of a beige solid (47.3 µmol, 36%).

**^1^H-NMR** (600 MHz, CDCl_3_)**:** δ [ppm] = 7.77–7.73 (m, 1 H), 7.68 (bs, 0.3 H), 7.61 (bs, 0.6 H), 7.31–7.17 (m, 2 H)*, 4.12 (t, *J* = 7.4 Hz, 0.3 H), 4.02 (t, *J* = 7.4 Hz, 0.7 H), 3.32 (s, 1 H), 3.09 (s, 2 H), 1.97–1.90 (m, 0.7 H), 1.85–1.78 (m, 0.7 H), 1.66–1.61 (m, 0.7 H)**, 1.59–1.54 (m, 0.3 H), 1.40–1.35 (m, 0.7 H), 0.91 (d, *J* = 6.6 Hz, 2 H), 0.89 (d, *J* = 6.6 Hz, 2 H), 0.88 (d, *J* = 6.6 Hz, 1 H), 0.84 (d, *J* = 6.6 Hz, 1 H).

**^13^C-NMR** (151 MHz, CDCl_3_)**:** δ [ppm] = 169.6, 167.9, 152.1 (d, *J* = 247 Hz), 129.1, 126.8 (d, *J* = 3.5 Hz), 125.7 (d, *J* = 8.1 Hz), 124.8 (d, *J* = 12.7 Hz), 118.4 (d, *J* = 19.7 Hz), 53.8, 34.9, 29.4, 25.0, 22.7, 22.5.

**^19^F-NMR** (282 MHz, CDCl_3_)**:** δ [ppm] = –128.30 (s, 0.7 F), –128.91 (s, 0.3 F).

*Signal overlaps with solvent residual peak.

**Signal overlaps with water peak.

NMR spectra showed a mixture of conformers. Only the ^13^C-signals of the major conformer are given.

**HR-MS** (APCI+)**:** *m/z* calcd. for C_14_H_17_FN_2_O_2_ [M+H]^+^: 265.1347, found: 265.1349.

**HPLC:** *t*_R_ = 9.4 min.

**LC-MS:** *t*_R_ = 11.0 min.

**Specific rotation:** [α]_D_ = +15.9 °·mL·dm^–1^·g^–1^ (ρ = 0.37; MeOH).

(*S*)-6-chloro-3-isobutyl-4-methyl-3,4-dihydro-1*H*-benzo[1,4]diazepine-2,5-dione (**9e**)

This compound was obtained after purification by preparative HPLC, yielding 6.5 mg of a white solid (23.7 µmol, 18%).

**^1^H-NMR** (600 MHz, CDCl_3_)**:** δ [ppm] = 8.47 (bs, 0.3 H), 8.44 (bs, 0.6 H), 8.37–8.29 (m, 2 H), 6.94 (dd, *J* = 7.3, 1.7 Hz, 0.8 H), 6.90 (dd, *J* = 7.5, 1.7 Hz), 4.11 (t, *J* = 7.4 Hz, 0.8 H), 4.06–4.02 (m, 0.2 H), 3.31 (s, 0.5 H), 3.06 (s, 2.5 H), 1.83 (t, *J* = 7.2 Hz, 2 H), 1.68–1.62 (m, 1 H), 0.92 (d, *J* = 6.7 Hz, 2.5 H), 0.89 (d, *J* = 6.7 Hz, 2.5 H), 0.86 (d, *J* = 6.7 Hz, 0.5 H), 0.84 (d, *J* = 6.7 Hz, 0.5 H).

**^13^C-NMR** (151 MHz, CDCl_3_)**:** δ [ppm] = 171.0, 166.1, 137.2, 135.1, 131.7, 127.9, 126.7, 119.3, 53.6, 34.5, 28.5, 24.9, 22.8, 22.3.

NMR spectra showed a mixture of conformers. Only the ^13^C-signals of the major conformer are given.

**HR-MS** (APCI+)**:** *m/z* calcd. for C_14_H_17_ClN_2_O_2_ [M+H]^+^: 281.1052, 283.1022; found: 281.1054, 283.1024.

**HPLC:** *t*_R_ = 10.1 min.

**LC-MS:** *t*_R_ = 11.8 min.

**Specific rotation:** [α]_D_ = +59.2 °·mL·dm^–1^·g^–1^ (ρ = 0.35; MeOH).

(*S*)-7-chloro-3-isobutyl-4-methyl-3,4-dihydro-1*H*-benzo[1,4]diazepine-2,5-dione (**9f**)

This compound was obtained after purification by preparative HPLC, yielding 8.9 mg of a white solid (31.7 µmol, 24%).

**^1^H-NMR** (600 MHz, CDCl_3_)**:** δ [ppm] = 9.00 (bs, 0.3 H), 8.74 (bs, 0.6 H), 7.94 (d, *J* = 2.5 Hz, 0.4 H), 7.92 (d, *J* = 2.5 Hz, 0.6 H), 7.45–7.40 (m, 1 H), 6.97 (d, *J* = 8.6 Hz, 0.6 H), 6.93 (d, *J* = 8.6 Hz, 0.4 H), 4.09 (bt, *J* = 7.4 Hz, 0.4 H), 3.99 (t, *J* = 7.4 Hz, 0.6 H), 3.31 (s, 1 H), 3.07 (s, 2 H), 1.96–1.89 (m, 0.7 H), 1.83–1.77 (m, 0.7 H), 1.66–1.59 (m, 0.7 H), 1.58–1.54 (m, 0.3 H), 1.41–1.36 (m, 0.6 H), 0.90 (dd, *J* = 6.6 Hz, 2 H), 0.89 (d, *J* = 6.6 Hz, 2 H), 0.87 (d, *J* = 6.6 Hz, 1 H), 0.85 (d, *J* = 6.6 Hz, 1 H).

**^13^C-NMR** (151 MHz, CDCl_3_)**:** δ [ppm] = 170.5, 167.6, 134.3, 132.6, 131.2, 131.1, 128.9, 122.2, 53.7, 34.8, 29.4, 25.0, 22.7, 22.6.

NMR spectra showed a mixture of conformers. Only the ^13^C-signals of the major conformer are given.

**HR-MS** (APCI+)**:** *m/z* calcd. for C_14_H_17_ClN_2_O_2_ [M+H]^+^: 281.1052, 283.1022; found: 281.1051, 283.1027.

**HPLC:** *t*_R_ = 10.8 min.

**LC-MS:** *t*_R_ = 12.5 min.

**Specific rotation:** [α]_D_ = +57.6 °·mL·dm^–1^·g^–1^ (ρ = 0.47; MeOH).

(*S*)-8-chloro-3-isobutyl-4-methyl-3,4-dihydro-1*H*-benzo[1,4]diazepine-2,5-dione (**9g**)

This compound was obtained after purification by MPLC, yielding 12.5 mg of a white solid (44.5 µmol, 33%).

**^1^H-NMR** (600 MHz, CDCl_3_)**:** δ [ppm] = 9.15 (s, 0.4 H), 8.96 (s, 0.6 H), 7.94–7.88 (m, 1 H), 7.25 (dd, *J* = 8.5, 2.0 Hz, 0.6 H), 7.21 (dd, *J* = 8.5, 2.0 Hz, 0.4 H), 7.05 (d, *J* = 2.0 Hz, 0.6 H), 7.02 (d, *J* = 2.1 Hz, 0.4 H), 4.10 (t, *J* = 7.4 Hz, 0.4 H), 3.99 (t, *J* = 7.4 Hz, 0.6 H), 3.31 (s, 1.1 H), 3.08 (s, 1.9 H), 1.98–1.91 (m, 0.7 H), 1.83–1.77 (m, 0.7 H), 1.65–1.59 (m, 0.6 H), 1.58–1.53 (m, 0.4 H), 1.41–1.36 (m, 0.6 H), 0.90 (d, *J* = 6.6 Hz, 2 H), 0.89 (d, *J* = 6.6 Hz, 2 H), 0.88 (d, *J* = 6.6 Hz, 1 H), 0.84 (d, *J* = 6.7 Hz, 1 H).

**^13^C-NMR** (151 MHz, CDCl_3_)**:** δ [ppm] = 170.7, 168.2, 138.5, 136.8, 133.0, 125.8, 125.4, 120.7, 53.8, 26.8, 29.4, 25.1, 22.7, 22.6.

**HR-MS** (APCI+)**:** *m/z* calcd. for C_14_H_17_ClN_2_O_2_ [M+H]^+^: 281.1052, 283.1022; found: 281.1051, 253.1031.

**HPLC:** *t*_R_ = 10.7 min.

**LC-MS:** *t*_R_ = 12.5 min.

**Specific rotation:** [α]_D_ = +56.2 °·mL·dm^–1^·g^–1^ (ρ = 0.51; MeOH).

(*S*)-3-isobutyl-4,6-dimethyl-3,4-dihydro-1*H*-benzo[1,4]diazepine-2,5-dione (**9i**)

This compound was obtained after purification by preparative HPLC, yielding 1.0 mg of a white solid (3.8 µmol, 3%).

**^1^H-NMR** (600 MHz, CDCl_3_)**:** δ [ppm] = 7.70 (bs, 0.9 H), 7.32–7.27 (m, 1 H), 7.13 (dt, *J* = 7.6, 0.9 Hz, 0.8 H), 7.09 (bd, *J* = 7.7 Hz, 0.2 H), 6.81 (bd, *J* = 8.0 Hz, 0.8 H), 6.75 (bd, *J* = 8.0 Hz, 0.2 H), 4.10 (t, *J* = 7.5 Hz, 0.8 H), 4.06–4.01 (m, 0.2 H), 3.31 (s, 0.5 H), 3.06 (s, 2.5 H), 2.54 (s, 2.5 H), 2.49 (s, 0.5 H), 1.83 (t, *J* = 7.3 Hz, 2 H), 1.67–1.62 (m, 1 H), 0.91 (d, *J* = 6.6 Hz, 2.3 H), 0.87 (d, *J* = 6.6 Hz, 2.3 H), 0.85 (d, *J* = 6.6 Hz, 0.7 H), 0.81 (d, *J* = 6.6 Hz, 0.7 H).

**^13^C-NMR** (151 MHz, CDCl_3_)**:** δ [ppm] = 171.1, 168.6, 140.6, 135.7, 130.9, 128.5, 127.2, 118.3, 26.6, 34.6, 28.5, 25.0, 22.9, 22.3, 21.0.

NMR spectra showed a mixture of conformers. Only the ^13^C-signals of the major conformer are given.

**HR-MS** (APCI+)**:** *m/z* calcd. for C_15_H_20_N_2_O_2_ [M+H]^+^: 261.1598, found: 261.1596.

**HPLC:** *t*_R_ = 10.1 min.

**LC-MS:** *t*_R_ = 11.8 min.

**Specific rotation:** [α]_D_ = +29.2 °·mL·dm^–1^·g^–1^ (ρ = 0.12; MeOH).

(*S*)-3-isobutyl-4,7-dimethyl-3,4-dihydro-1*H*-benzo[1,4]diazepine-2,5-dione (**9j**)

This compound was obtained after purification by MPLC, yielding 23.8 mg of a white solid (91.4 µmol, 68%).

**^1^H-NMR** (600 MHz, CDCl_3_)**:** δ [ppm] = 9.10 (bs, 0.3 H), 8.85 (bs, 0.6 H), 7.74 (d, *J* = 2.4 Hz, 0.3 H), 7.73 (d, *J* = 2.2 Hz, 0.7 H), 7.28 (dd, *J* = 8.2, 2.1 Hz, 0.7 H), 7.25 (dd, *J* = 8.2, 2.1 Hz, 0.3 H)*, 6.92 (d, *J* = 8.1 Hz, 0.7 H), 6.88 (d, *J* = 8.1 Hz, 0.3 H), 4.07 (t, *J* = 7.4 Hz, 0.3 H), 4.01 (t, *J* = 7.4 Hz, 0.7 H), 3.31 (s, 1 H), 3.07 (s, 2 H), 2.37 (s, 2 H), 2.36 (s, 1 H), 1.95–1.88 (m, 0.7 H), 1.82–1.76 (m, 0.7 H), 1.65–1.59 (m, 0.7 H), 1.58–1.52 (m, 0.3 H), 1.39–1.34 (m, 0.6 H), 0.88 (d, *J* = 6.6 Hz, 1.9 H), 0.87 (d, *J* = 6.6 Hz, 1.9 H), 0.85 (d, *J* = 6.6 Hz, 1.1 H), 0.82 (d, *J* = 6.6 Hz, 1.1 H).

**^13^C-NMR** (151 MHz, CDCl_3_)**:** δ [ppm] = 170.9, 169.2, 135.5, 133.5, 133.4, 131.5, 127.2, 120.7, 53.7, 34.9, 29.3, 25.0, 22.7, 22.5, 20.9.

*Signal overlaps with solvent residual peak.

NMR spectra showed a mixture of conformers. Only the ^13^C-signals of the major conformer are given.

**HR-MS** (APCI+)**:** *m/z* calcd. for C_15_H_20_N_2_O_2_ [M+H]^+^: 261.1598, found: 261.1596.

**HPLC:** *t*_R_ = 10.5 min.

**LC-MS:** *t*_R_ = 11.9 min.

**Specific rotation:** [α]_D_ = +56.3 °·mL·dm^–1^·g^–1^ (ρ = 1.04; MeOH).

(*S*)-3-isobutyl-4,9-dimethyl-3,4-dihydro-1*H*-benzo[1,4]diazepine-2,5-dione (**9k**)

This compound was obtained after purification by MPLC, yielding 4.2 mg of a slightly yellow solid (16.1 µmol, 12%).

**^1^H-NMR** (600 MHz, CDCl_3_)**:** δ [ppm] = 7.78 (dd, *J* = 7.9, 1.6 Hz, 1 H), 7.38–7.29 (m, 2 H), 7.21 (t, *J* = 7.6 Hz, 0.8 H), 7.16 (t, *J* = 7.7 Hz, 0.2 H), 4.09 (t, *J* = 7.4 Hz, 0.2 H), 4.02 (t, *J* = 7.4 Hz, 0.8 H), 3.32 (s, 0.8 H), 3.09 (s, 2.2 H), 2.33 (s, 2.2 H), 2.32 (s, 0.8 H), 1.93–1.87 (m, 0.8 H), 1.86–1.81 (m, 0.8 H), 1.67–1.61 (m)*, 1.56–1.52 (m, 0.2 H), 1.32–1.24 (m, 0.8 H), 0.90 (d, *J* = 6.7 Hz, 2.2 H), 0.89 (d, *J* = 6.7 Hz, 2.2 H), 0.85 (d, *J* = 6.7 Hz, 0.8 H), 0.81 (d, *J* = 6.7 Hz, 0.8 H).

**^13^C-NMR** (151 MHz, CDCl_3_)**:** δ [ppm] = 170.2, 169.4, 134.0, 133.8, 129.3, 128.4, 128.3, 125.7, 53.7, 34.8, 29.3, 25.1, 22.8, 22.5, 18.2.

*Signal overlaps with water signal.

NMR spectra showed a mixture of conformers. Only the ^13^C-signals of the major conformer are given.

**HR-MS** (APCI+)**:** *m/z* calcd. for C_15_H_20_N_2_O_2_ [M+H]^+^: 261.1598, found: 261.1598.

**HPLC:** *t*_R_ = 10.1 min.

**LC-MS:** *t*_R_ = 11.7 min.

**Specific rotation:** [α]_D_ = +40.5 °·mL·dm^–1^·g^–1^ (ρ = 0.23; MeOH).

(*S*)-3-isobutyl-4-methyl-3,4-dihydro-1*H*-naphtho[2,3-*e*][1,4]diazepine-2,5-dione (**9l**)

This compound was obtained after purification by MPLC, yielding 13.2 mg of a yellowish solid (44.5 µmol, 33%).

**^1^H-NMR** (600 MHz, CDCl_3_)**:** δ [ppm] = 8.56 (s, 0.4 H), 8.54 (bs, 0.3 H), 8.52 (s, 0.6 H), 8.30 (bs, 0.6 H), 7.94 (bt, *J* = 7.2 Hz, 1 H), 7.79 (bt, *J* = 7.1 Hz, 1 H), 7.59–7.54 (m, 1 H), 7.52–7.47 (m, 1 H), 7.44 (s, 0.6 H), 7.38 (s, 0.4 H), 4.14 (bt, *J* = 7.3 Hz, 1 H), 3.38 (s, 1 H), 3.15 (s, 2 H), 1.98–1.91 (m, 0.7 H), 1.85–1.79 (m, 0.7 H), 1.67–1.61 (m, 0.6 H), 1.61–1.54 (m, 0.4 H), 0.87 (d, *J* = 6.7 Hz, 1.9 H), 0.86 (d, *J* = 6.7 Hz, 1.9 H), 0.83 (d, *J* = 6.5 Hz, 1.1 H), 0.79 (d, *J* = 6.7 Hz, 1.1 H).

**^13^C-NMR** (151 MHz, CDCl_3_)**:** δ [ppm] = 171.2, 169.1, 134.9, 132.7, 132.5, 130.7, 129.1, 127.9, 127.4, 127.1, 126.5, 118.0, 53.7, 34.9, 29.3, 25.1, 22.7, 22.5.

NMR spectra showed a mixture of conformers. Only the ^13^C-signals of the major conformer are given.

**HR-MS** (APCI+)**:** *m/z* calcd. for C_18_H_20_N_2_O_2_ [M+H]^+^: 297.1598, found: 297.1597.

**HPLC:** *t*_R_ = 12.1 min.

**LC-MS:** *t*_R_ = 13.5 min.

**Specific rotation:** [α]_D_ = +55.7 °·mL·dm^–1^·g^–1^ (ρ = 0.36; MeOH).

(*S*)-4-ethyl-3-isobutyl-3,4-dihydro-1*H*-benzo[1,4]diazepine-2,5-dione (**9m**)

This compound was obtained after purification by preparative HPLC, yielding 4.0 mg (15 µmol, 6%) of a white solid.

**^1^H-NMR** (600 MHz, CDCl_3_)**:** δ [ppm] = 8.15 (bs, 0.6 H), 8.04 (bs, 0.4 H), 7.96–7.93 (m, 1 H), 7.49–7.44 (m, 1 H), 7.29 (td, *J* = 7.6, 1.1 Hz, 0.4 H), 7.24 (td, *J* = 7.6, 1.1 Hz, 0.6 H), 6.96 (dd, *J* = 8.0, 1.1 Hz, 0.4 H), 6.91 (dd, *J* = 8.0, 1.1 Hz, 0.6 H), 4.12–4.00 (m, 2 H), 3.46 (dq, *J* = 14.1, 7.1 Hz, 0.6 H), 3.27 (dq, *J* = 14.1, 7.0 Hz, 0.4 H), 2.05–1.98 (m, 0.4 H), 1.81–1.75 (m, 0.4 H), 1.64 (h, *J* = 6.7 Hz, 0.4 H), 1.57 (h, *J* = 6.7 Hz, 0.6 H), 1.44–1.38 (m, 0.6 H), 1.35–1.31 (m, 0.6 H), 1.28 (t, *J* = 7.2 Hz, 1.8 H), 1.21 (t, *J* = 7.1 Hz, 1.2 H), 0.90 (d, *J* = 6.6 Hz, 1 H), 0.88 (d, *J* = 6.6 Hz, 1 H), 0.86 (d, *J* = 6.6 Hz, 2 H), 0.83 (d, *J* = 6.6 Hz, 2 H).

**^13^C-NMR** (151 MHz, CDCl_3_)**:** δ [ppm] = 172.6, 165.8, 134.5, 132.6, 131.7, 127.4, 125.2, 119.8, 63.3, 46.6, 38.7, 25.6, 22.6, 22.5, 13.5.

NMR spectra showed a mixture of conformers. Only the ^13^C-signals of the major conformer are given.

**HR-MS** (APCI+)**:** *m/z* calcd. for C_15_H_20_N_2_O_2_ [M+H]^+^: 261.1598, found: 261.1599.

**HPLC:** *t*_R_ = 10.3 min.

**LC-MS:** *t*_R_ = 11.5 min.

**Specific rotation:** [α]_D_ = –44.5 °·mL·dm^–1^·g^–1^ (ρ = 0.23; MeOH).

(*R*)-3-benzyl-4-methyl-3,4-dihydro-1*H*-benzo[1,4]diazepine-2,5-dione ((*R*)-**10**)

This compound was synthesized starting from d-Fmoc-phenylalanine, yielding 29.8 mg of a beige solid (0.11 mmol, 34%).

**^1^H-NMR** (600 MHz, CDCl_3_)**:** δ [ppm] = 9.37 (s, 0.4 H), 9.06 (s, 0.6 H), 8.10 (dd, *J* = 7.9, 1.6 Hz, 0.4 H), 7.95 (dd, *J* = 7.9, 1.6 Hz, 0.6 H), 7.54 (td, *J* = 7.6, 1.6 Hz, 0.4 H), 7.47 (td, *J* = 7.7, 1.6 Hz, 0.6 H), 7.35–7.20 (m, 5 H)*, 7.10–6.96 (m, 2 H), 4.35 (dd, *J* = 8.3, 6.5 Hz, 0.6 H), 4.29–4.25 (m, 0.4 H), 3.49 (dd, *J* = 14.5, 8.4 Hz, 0.6 H), 3.22 (dd, *J* = 14.5, 6.5 Hz, 0.6 H), 3.15 (s, 1.7 H), 2.92 (s, 1.3 H), 2.84 (dd, *J* = 13.8, 6.2 Hz, 0.4 H), 2.71 (dd, *J* = 13.7, 11.1 Hz, 0.4 H).

**^13^C-NMR** (151 MHz, CDCl_3_)**:** δ [ppm] = 170.2, 168.8, 136.4, 135.6, 132.7, 131.5, 129.1 (2C), 128.9 (2C), 127.5, 127.2, 125.6, 120.9, 56.5, 32.3, 29.5.

*Signal overlaps with solvent residual peak.

NMR spectra showed a mixture of conformers. Only the ^13^C signals of the major conformer are given.

**HR-MS** (APCI+)**:** *m/z* calcd. for C_17_H_16_N_2_O_2_ [M+H]^+^: 281.1285, found: 281.1285.

**HPLC:** *t*_R_ = 10.0 min.

**LC-MS:** *t*_R_ = 11.5 min.

**Specific rotation:** [α]_D_ = +20.4 °·mL·dm^–1^·g^–1^ (ρ = 0.89; MeOH).

(*S*)-3-benzyl-6-fluoro-4-methyl-3,4-dihydro-1*H*-benzo[1,4]diazepine-2,5-dione (**10a**)

This compound was obtained after purification by MPLC, yielding 10.9 mg of a white solid (36.5 µmol, 27%).

**^1^H-NMR** (600 MHz, CDCl_3_)**:** δ [ppm] = 8.90 (bs, 0.2 H), 8.64 (bs, 0.8 H), 7.49–7.44 (m, 0.2 H), 7.42–7.37 (m, 0.8 H), 7.30–7.20 (m, 4.5 H)*, 7.08–6.98 (m, 1.5 H), 6.88 (bd, *J* = 8.1 Hz, 0.2 H), 6.78 (bd, *J* = 8.1 Hz, 0.8 H), 4.43 (dd, *J* = 8.5, 6.3 Hz, 0.8 H), 4.23 (dd, *J* = 11.3, 6.2 Hz, 0.2 H), 3.47 (dd, *J* = 14.4, 8.5 Hz, 0.8 H), 3.20 (dd, *J* = 14.2, 6.1 Hz, 0.8 H), 3.12 (s, 2.3 H), 2.92 (s, 0.7 H), 2.85 (dd, *J* = 13.8, 6.0 Hz, 0.2 H), 2.72 (dd, *J* = 13.7, 11.2 Hz, 0.2 H).

**^13^C-NMR** (151 MHz, CDCl_3_)**:** δ [ppm] = 170.0, 164.4, 161.9 (d, *J* = 257 Hz), 137.0 (d, *J* = 4.6 Hz), 136.2, 132.8 (d, *J* = 10.8 Hz), 129.2 (2C), 128.9 (2C), 127.3, 116.7 (d, *J* = 15.0 Hz), 116.6 (d, *J* = 3.5 Hz), 113.5 (d, *J* = 22.0 Hz), 56.5, 32.2, 28.8.

**^19^F-NMR** (282 MHz, CDCl_3_)**:** δ [ppm] = –108.39 (s, 0.2 F), –108.90 (s, 0.8 F).

*Signal overlaps with solvent residual peak.

NMR spectra showed a mixture of conformers. Only the ^13^C-signals of the major conformer are given.

**HR-MS** (APCI+)**:** *m/z* calcd. for C_17_H_15_FN_2_O_2_ [M+H]^+^: 2299.1190, found: 299.1189.

**HPLC:** *t*_R_ = 10.0 min.

**LC-MS:** *t*_R_ = 11.7 min.

**Specific rotation:** [α]_D_ = +32.3 °·mL·dm^–1^·g^–1^ (ρ = 0.36; MeOH).

(*S*)-3-benzyl-7-fluoro-4-methyl-3,4-dihydro-1*H*-benzo[1,4]diazepine-2,5-dione (**10b**)

This compound was obtained after purification by preparative HPLC, yielding 5.2 mg of a white solid (17.4 µmol, 13%).

**^1^H-NMR** (600 MHz, CDCl_3_)**:** δ [ppm] = 9.15 (bs, 0.4 H), 8.77 (bs, 0.6 H), 7.82 (dd, *J* = 9.0, 3.0 Hz, 0.4 H), 7.65 (dd, *J* = 8.7, 3.0 Hz, 0.6 H), 7.31–7.17 (m, 5.2 H)*, 7.05 (dd, *J* = 8.8, 4.5 Hz, 0.4 H), 7.01 (bd, *J* = 7.1 Hz, 0.8 H), 6.95 (dd, *J* = 8.8, 4.5 Hz, 0.6 H), 4.33 (dd, *J* = 8.4, 6.4 Hz, 0.6 H), 4.27 (dd, *J* = 11.2, 6.1 Hz, 0.4 H), 3.49 (dd, *J* = 14.5, 8.4 Hz, 0.6 H), 3.21 (dd, *J* = 14.4, 6.4 Hz, 0.6 H), 3.15 (s, 1.7 H), 2.92 (s, 1.3 H), 2.86 (dd, *J* = 13.8, 6.1 Hz, 0.4 H), 2.72 (dd, *J* = 13.8, 6.1 Hz).

**^13^C-NMR** (151 MHz, CDCl_3_)**:** δ [ppm] = 169.9, 167.3, 159.9 (d, *J* = 248 Hz), 136.3, 131.8, 130.9, 129.1 (2C), 129.0 (2C), 127.3, 122.8 (d, *J* = 7.8 Hz), 120.1 (d, *J* = 23.1 Hz), 117.8 (d, *J* = 24.3 Hz), 56.5, 32.3, 29.6.

**^19^F-NMR** (282 MHz, CDCl_3_)**:** δ [ppm] = –115.25 (s, 0.6 F), –115.67 (s, 0.4 F).

*Signal overlaps with solvent residual peak.

NMR spectra showed a mixture of conformers. Only the ^13^C-signals of the major conformer are given.

**HR-MS** (APCI+)**:** *m/z* calcd. for C_17_H_15_FN_2_O_2_ [M+H]^+^: 299.1190, found: 299.1188.

**HPLC:** *t*_R_ = 10.4 min.

**LC-MS:** *t*_R_ = 12.1 min.

**Specific rotation:** [α]_D_ = –51.5 °·mL·dm^–1^·g^–1^ (ρ = 0.26; MeOH).

(*S*)-3-benzyl-8-fluoro-4-methyl-3,4-dihydro-1*H*-benzo[1,4]diazepine-2,5-dione (**10c**)

This compound was obtained after purification by preparative HPLC, yielding 17.5 mg of a white solid (58.7 µmol, 49%).

**^1^H-NMR** (600 MHz, CDCl_3_)**:** δ [ppm] = 9.44 (bs, 0.4 H), 9.15 (bs, 0.5 H), 8.14 (dd, *J* = 8.9, 6.2 Hz, 0.4 H), 7.98 (dd, *J* = 8.8, 6.1 Hz, 0.6 H), 7.31–7.20 (m, 4.2 H)*, 7.07–6.98 (m, 1.8 H), 6.80 (dd, *J* = 9.1, 2.4 Hz, 0.4 H), 6.70 (dd, *J* = 9.1, 2.4 Hz, 0.6 H), 4.33 (dd, *J* = 8.5, 6.2 Hz, 0.6 H), 4.28 (dd, *J* = 11.0, 6.3 Hz, 0.4 H), 3.49 (dd, *J* = 14.4, 8.6 Hz, 0.6 H), 3.22 (dd, *J* = 14.4, 6.3 Hz, 0.6 H), 3.15 (s, 1.7 H), 2.92 (s, 1.3 H), 2.87 (dd, *J* = 13.7, 6.2 Hz, 0.4 H), 2.75 (dd, *J* = 13.7, 11.0 Hz, 0.4 H).

**^13^C-NMR** (151 MHz, CDCl_3_)**:** δ [ppm] = 170.1, 168.0, 164.8 (d, *J* = 224 Hz), 137.5, 136.2, 134.2 (d, *J* = 10.4 Hz), 129.1 (2C), 129.0 (2C), 127.3, 123.0, 113.3 (d, *J* = 23.0 Hz), 107.8 (d, *J* = 25.2 Hz), 56.7, 32.3, 29.7.

**^19^F-NMR** (282 MHz, CDCl_3_)**:** δ [ppm] = –105.34 (s, 0.4 F), –105.45 (s, 0.6 F).

*Signal overlaps with solvent residual peak.

NMR spectra showed a mixture of conformers. Only the ^13^C-signals of the major conformer are given.

**HR-MS** (APCI+)**:** *m/z* calcd. for C_17_H_15_FN_2_O_2_ [M+H]^+^: 299.1190, found: 299.1190.

**HPLC:** *t*_R_ = 10.3 min.

**LC-MS:** *t*_R_ = 12.0 min.

**Specific rotation:** [α]_D_ = –13.9 °·mL·dm^–1^·g^–1^ (ρ = 0.54; MeOH).

(*S*)-3-benzyl-9-fluoro-4-methyl-3,4-dihydro-1*H*-benzo[1,4]diazepine-2,5-dione (**10d**)

This compound was obtained after purification by preparative HPLC, yielding 8.3 mg of a white solid (27.8 µmol, 21%).

**^1^H-NMR** (600 MHz, CDCl_3_)**:** δ [ppm] = 8.10 (bs, 0.4 H), 8.00 (bs, 0.6 H), 7.91 (bd, *J* = 7.9 Hz), 7.75 (bd, *J* = 7.9 Hz, 0.6 H), 7.37–7.21 (m, 6.2 H)*, 7.01 (bd, *J* = 7.4 Hz, 0.8 H), 4.35 (dd, *J* = 8.2, 6.6 Hz, 0.6 H), 4.32–4.28 (m, 0.4 H), 3.51 (dd, *J* = 14.6, 8.2 Hz, 0.6 H), 3.23 (dd, *J* = 14.6, 6.6 Hz, 0.6 H), 3.14 (s, 1.7 H), 2.93–2.85 (m, 1.7 H), 2.70 (dd, *J* = 13.8, 11.3 Hz, 0.4 H).

**^13^C-NMR** (151 MHz, CDCl_3_)**:** δ [ppm] = 170.3, 168.9, 167.5, 165.1, 152.2 (d, *J* = 247 Hz), 136.2, 135.4, 129.2 (2C), 129.1 (2C), 129.00, 128.96 (2C), 128.93 (2C), 128.5, 127.7, 127.3, 126.9, 125.7 (d, *J* = 8.1 Hz), 125.5 (d, *J* = 8.1 Hz), 124.7 (d, *J* = 12.7 Hz), 118.7, 118.5 (d, *J* = 19.7 Hz), 68.6, 56.6, 40.0, 35.0, 32.3, 29.6.

**^19^F-NMR** (282 MHz, CDCl_3_)**:** δ [ppm] = –127.16 (s, 0.6 F), –127.74 (s, 0.4 F).

*Signal overlaps with solvent residual peak.

NMR spectra showed a mixture of conformers. Due to ^13^C-signal overlapping in the aromatic region, it was not possible to completely assign the major conformer. Therefore, all signals are given.

**HR-MS** (APCI+)**:** *m/z* calcd. for C_17_H_15_FN_2_O_2_ [M+H]^+^: 299.1190, found: 299.1191.

**HPLC:** *t*_R_ = 10.1 min.

**LC-MS:** *t*_R_ = 11.7 min.

**Specific rotation:** [α]_D_ = –13.6 °·mL·dm^–1^·g^–1^ (ρ = 0.42; MeOH).

(*S*)-3-benzyl-6-chloro-4-methyl-3,4-dihydro-1*H*-benzo[1,4]diazepine-2,5-dione (**10e**)

This compound was obtained after purification by preparative HPLC, yielding 3.0 mg of a white solid (9.5 µmol, 7%).

**^1^H-NMR** (600 MHz, CDCl_3_)**:** δ [ppm] = 7.78 (bs, 0.2 H), 7.63 (bs, 0.6 H), 7.34–7.21 (m, 6.6 H)*, 7.10–7.08 (m, 0.4 H), 6.93 (dd, *J* = 7.1, 2.0 Hz, 0.3 H), 6.85 (dd, *J* = 6.4, 2.8 Hz, 0.7 H), 4.44 (dd, *J* = 8.4, 6.4 Hz, 0.7 H), 4.23 (dd, *J* = 11.4, 5.4 Hz, 0.3 H), 3.46 (dd, *J* = 14.5, 8.4 Hz, 0.7 Hz), 3.20 (dd, *J* = 14.5, 6.4 Hz, 0.7 H), 3.12 (s, 2.3 H), 2.94 (s, 0.7 H), 2.82 (dd, *J* = 13.9, 5.5 Hz, 0.3 H), 2.64 (dd, *J* = 13.9, 12.1 Hz, 0.3 H).

**^13^C-NMR** (151 MHz, CDCl_3_)**:** δ [ppm] = 170.0, 165.6, 136.8, 136.0, 135.1, 131.7, 129.0 (2C), 128.8 (2C), 128.0, 127.1, 126.5, 119.4, 56.2, 32.0, 28.6.

*Signal overlaps with solvent residual peak.

NMR spectra showed a mixture of conformers. Only the ^13^C-signals of the major conformer are given.

**HR-MS** (APCI+)**:** *m/z* calcd. for C_17_H_15_ClN_2_O_2_ [M+H]^+^: 315.0895, 317.0866; found: 315.0899, 317.0878.

**HPLC:** *t*_R_ = 10.9 min.

**LC-MS:** *t*_R_ = 12.5 min.

**Specific rotation:** [α]_D_ = +7.8 °·mL·dm^–1^·g^–1^ (ρ = 0.2; MeOH).

(*S*)-3-benzyl-7-chloro-4-methyl-3,4-dihydro-1*H*-benzo[1,4]diazepine-2,5-dione (**10f**)

This compound was obtained after purification by preparative HPLC, yielding 10.0 mg of a white solid (31.8 µmol, 24%).

**^1^H-NMR** (600 MHz, CDCl_3_)**:** δ [ppm] = 9.31 (bs, 0.4 H), 8.94 (bs, 0.5 H), 8.09 (d, *J* = 2.5 Hz, 0.4 H), 7.92 (d, *J* = 2.5 Hz, 0.6 H), 7.48 (dd, *J* = 8.5, 2.5 Hz, 0.4 H), 7.42 (dd, *J* = 8.5, 2.5 Hz, 0.6 H), 7.31 (m, 1.9 H), 7.26–7.20 (m, 2.4 H), 7.02 (bd, *J* = 8.3 Hz, 1,2 H), 6.91 (d, *J* = 8.6 Hz, 0.4 H), 4.31 (dd, *J* = 8.4, 6.4 Hz, 0.6 H), 4.26 (dd, *J* = 11.1, 6.3 Hz, 0.4 H), 3.48 (dd, *J* = 14.5, 8.4 Hz, 0.6 H), 3.21 (dd, *J* = 14.5, 6.4 Hz, 0.6 H), 3.14 (s, 1.7 H), 2.91 (s, 1.3 H), 2.86 (dd, *J* = 13.8, 6.2 Hz, 0.4 H), 2.73 (dd, *J* = 13.7, 11.0 Hz, 0.4 H).

**^13^C-NMR** (151 MHz, CDCl_3_)**:** δ [ppm] = 169.9, 167.3, 136.2, 134.1, 132.7, 131.3, 131.2, 129.1 (2C), 128.9 (2C), 128.8, 127.3, 122.4, 56.5, 32.3, 29.6.

NMR spectra showed a mixture of conformers. Only the ^13^C-signals of the major conformer are given.

**HR-MS** (APCI+)**:** *m/z* calcd. for C_17_H_15_ClN_2_O_2_ [M+H]^+^: 315.0895, 317.0866; found: 315.0897, 317.0880.

**HPLC:** *t*_R_ = 11.5 min.

**LC-MS:** *t*_R_ = 13.1 min.

**Specific rotation:** [α]_D_ = –41.5 °·mL·dm^–1^·g^–1^ (ρ = 0.5; MeOH).

(*S*)-3-benzyl-8-chloro-4-methyl-3,4-dihydro-1*H*-benzo[1,4]diazepine-2,5-dione (**10g**)

This compound was obtained after purification by preparative HPLC, yielding 14.7 mg of a white solid (46.7 µmol, 35%).

**^1^H-NMR** (600 MHz, CDCl_3_)**:** δ [ppm] = 9.37 (bs, 0.4 H), 9.09 (bs, 0.5 H), 8.07 (d, *J* = 8.5 Hz, 0.4 H), 7.90 (d, *J* = 8.5 Hz, 0.6 H), 7.32–7.20 (m, 5.2 H)*, 7.09 (d, *J* = 2.0 Hz, 0.4 H), 7.01 (bd, *J* = 7.1 Hz, 0.8 H), 6.98 (d, *J* = 1.9 Hz, 0.6 H), 4.33–4.25 (m, 1 H), 3.50 (dd, *J* = 14.3, 8.6 Hz, 0.6 H), 3.21 (dd, *J* = 14.3, 6.1 Hz, 0.6 H), 3.15 (s, 1.7 H), 2.92 (s, 1.3 H), 2.87 (dd, *J* = 13.8, 6.4 Hz, 0.4 H), 2.74 (dd, *J* = 13.7, 10.9 Hz, 0.4 H).

**^13^C-NMR** (151 MHz, CDCl_3_)**:** δ [ppm] = 170.1, 167.8, 138.5, 136.7, 136.3, 133.0, 129.2 (2C), 128.9 (2C), 127.3, 125.9, 125.7, 120.8, 56.7, 32.3, 29.6.

*Signal overlaps with solvent residual peak.

NMR spectra showed a mixture of conformers. Only the ^13^C-signals of the major conformer are given.

**HR-MS** (APCI+)**:** *m/z* calcd. for C_17_H_15_ClN_2_O_2_ [M+H]^+^: 315.0895, 317.0866; found: 317.0899, 315.0879.

**HPLC:** *t*_R_ = 11.5 min.

**LC-MS:** *t*_R_ = 13.1 min.

**Specific rotation:** [α]_D_ = –41.0 °·mL·dm^–1^·g^–1^ (ρ = 0.63; MeOH).

(S)-3-benzyl-4,6-dimethyl-3,4-dihydro-1H-benzo[1,4]diazepine-2,5-dione (**10i**)

This compound was obtained after purification by preparative HPLC, yielding 1.6 mg of a white solid (5.4 µmol, 4%).

**^1^H-NMR** (600 MHz, CDCl_3_)**:** δ [ppm] = 8.09 (bs, 0.2 H), 7.95 (bs, 0.7 H), 7.33–7.26 (m, 3 H), 7.25–7.17 (m, 3 H), 7.13 (d, *J* = 7.5 Hz, 0.8 H), 6.97 (bd, *J* = 6.9 Hz, 0.3 H), 6.87 (d, *J* = 7.9 Hz, 0.2 H), 6.79 (d, *J* = 7.9 Hz, 0.7 H), 4.44 (dd, *J* = 8.1, 6.8 Hz, 0.8 H), 4.25 (dd, *J* = 11.7, 5.6 Hz, 0.2 H), 3.44 (dd, *J* = 14.7, 8.1 Hz, 0.8 H), 3.19 (dd, *J* = 14.7, 6.8 Hz, 0.8 H), 3.11 (s, 2.3 H), 2.98 (s, 0.7 H), 2.81 (dd, *J* = 13.9, 6.0 Hz, 0.2 H), 2.61–2.56 (m, 0.9 H), 2.53 (s, 2.3 H).

**^13^C-NMR** (151 MHz, CDCl_3_)**:** δ [ppm] = 170.5, 168.3, 140.7, 136.4, 135.5, 131.1, 129.0 (2C), 128.9 (2C), 128.6, 127.2, 127.1, 118.6, 56.2, 32.2, 28.6, 21.1.

^1^H-NMR spectrum showed a mixture of conformers.

**HR-MS** (ESI+)**:** *m/z* calcd. for C_18_H_18_N_2_O_2_ [M+H]^+^: 295.1441, found: 295.1446.

**HPLC:** *t*_R_ = 10.9 min.

**LC-MS:** *t*_R_ = 12.5 min.

**Specific rotation:** [α]_D_ = +7.5 °·mL·dm^–1^·g^–1^ (ρ = 0.16; MeOH).

(*S*)-3-benzyl-4,7-dimethyl-3,4-dihydro-1*H*-benzo[1,4]diazepine-2,5-dione (**10j**)

This compound was obtained after purification by MPLC, yielding 21.0 mg of a white solid (71.3 µmol, 55%).

**^1^H-NMR** (600 MHz, CDCl_3_)**:** δ [ppm] = 9.23 (bs, 0.4 H), 8.86 (bs, 0.6 H), 7.90 (s, 0.4 H), 7.74 (s, 0.6 H), 7.34–7.18 (m, 5 H)*, 7.04–6.99 (m, 1 H), 6.97–6.93 (m, 0.4 H), 6.87–6.83 (m, 0.6 H), 4.35 (dd, *J* = 8.3, 6.6 Hz, 0.6 H), 4.25 (dd, *J* = 11.1, 6.2 Hz, 0.4 H), 3.48 (dd, *J* = 14.5, 8.3 Hz, 0.6 H), 3.21 (dd, *J* = 14.6, 6.6 Hz, 0.6 H), 3.14 (s, 1.7 H), 2.91 (s, 1.3 H), 2.83 (dd, *J* = 13.7, 6.1 Hz, 0.4 H), 2.73 (dd, *J* = 13.7, 11.2 Hz, 0.4 H).

**^13^C-NMR** (151 MHz, CDCl_3_)**:** δ [ppm] = 170.2, 168.8, 136.5, 135.5, 133.5, 133.2, 131.5, 129.1 (2C), 128.8 (2C), 127.5, 127.1, 120.8, 56.5, 32.3, 29.5, 20.9.

*Signal overlaps with solvent residual peak.

NMR spectra showed a mixture of conformers. Only the ^13^C-signals of the major conformer are given.

**HR-MS** (APCI+)**:** *m/z* calcd. for C_18_H_18_N_2_O_2_ [M+H]^+^: 295.1441, found: 295.1446.

**HPLC:** *t*_R_ = 11.2 min.

**LC-MS:** *t*_R_ = 12.5 min.

**Specific rotation:** [α]_D_ = –41.4 °·mL·dm^–1^·g^–1^ (ρ = 0.62; MeOH).

(*S*)-3-benzyl-4,9-dimethyl-3,4-dihydro-1*H*-benzo[1,4]diazepine-2,5-dione (**10k**)

This compound was obtained after purification by preparative HPLC, yielding 4.0 mg of a white solid (13.5 µmol, 10%).

**^1^H-NMR** (600 MHz, CDCl_3_)**:** δ [ppm] = 7.92 (dd, *J* = 8.0, 1.7 Hz, 0.3 H), 7.82–7.76 (m, 1.6 H), 7.42 (bd, *J* = 7.4 Hz, 0.3 H), 7.36 (bd, *J* = 7.4 Hz, 0.7 H), 7.30–7.19 (m, 5.3 H)*, 6.97 (bd, *J* = 6.9 Hz, 0.7 H), 4.37 (bt, *J* = 7.5 Hz, 0.7 H), 4.28 (dd, *J* = 10.8, 5.0 Hz, 0.3 H), 3.47 (dd, *J* = 14.7, 8.0 Hz, 0.7 H), 3.23 (dd, *J* = 14.7, 7.0 Hz, 0.7 H), 3.14 (s, 2 H), 2.97 (s, 1 H), 2.77 (dd, *J* = 13.7, 6.6 Hz, 0.3 H), 2.67 (dd, *J* = 13.8, 10.8 Hz, 0.3 H), 2.38 (s, 1 H), 2.30 (s, 2 H).

**^13^C-NMR** (151 MHz, CDCl_3_)**:** δ [ppm] = 170.4, 169.4, 168.9, 136.2, 135.4, 134.2, 133.9, 133.6, 132.5, 129.8, 129.3, 128.94 (2C), 128.85 (2C), 128.79 (2C), 128.75 (2C), 128.3, 128.0, 127.4, 127.0, 125.54, 125.46, 68.3, 56.1, 39.6, 34.4, 32.1, 29.2, 18.2, 18.1.

NMR spectra showed a mixture of conformers. Due to ^13^C-signal overlapping in the aromatic region, it was not possible to completely assign the major conformer. Therefore, all signals are given.

**HR-MS** (ESI+)**:** *m/z* calcd. for C_18_H_18_N_2_O_2_ [M+H]^+^: 295.1441, found: 295.1444.

**HPLC:** *t*_R_ = 10.9 min.

**LC-MS:** *t*_R_ = 12.3 min.

**Specific rotation:** [α]_D_ = +25.8 °·mL·dm^–1^·g^–1^ (ρ = 0.27; MeOH).

(*S*)-3-benzyl-4-methyl-3,4-dihydro-1*H*-naphtho[2,3-*e*][1,4]diazepine-2,5-dione (**10l**)

This compound was obtained after purification by preparative HPLC, yielding 20.0 mg of a white solid (60.5 µmol, 45%).

**^1^H-NMR** (600 MHz, CDCl_3_)**:** δ [ppm] = 9.29 (bs, 0.4 H), 8.86 (bs, 0.5 H), 8.71 (s, 0.4 H), 8.52 (s, 0.6 H), 8.00 (d, *J* = 8.2 Hz, 0.4 H), 7.93 (d, *J* = 8.2 Hz, 0.6 H), 7.82 (d, *J* = 8.2 Hz, 0.4 H), 7.75 (d, *J* = 8.1 Hz, 0.6 H), 7.62 –7.48 (m, 3 H), 7.41–7.27 (m, 1.1 H), 7.26–7.18 (m, 3.1 H), 7.02 (bd, *J* = 7.1 Hz, 0.8 H), 4.46 (dd, *J* = 8.3, 6.5 Hz, 0.6 H), 4.31 (dd, *J* = 9.5, 6.2 Hz, 0.4 H), 3.51 (dd, *J* = 14.5, 8.3 Hz, 0.6 H), 3.23 (dd, *J* = 14.4, 6.4 Hz), 3.21 (s, 1.6 H), 2.99 (s, 1.4 H), 2.86 (dd, *J* = 13.7, 6.1 Hz, 0.4 H), 2.71 (dd, *J* = 13.8, 11.0 Hz, 0.4 H).

**^13^C-NMR** (151 MHz, CDCl_3_)**:** δ [ppm] = 172.0, 170.6, 168.8, 166.3, 136.4, 135.7, 135.2, 134.9, 133.5, 132.8, 132.3, 131.6, 130.6, 130.6, 129.3, 129.08 (2C), 129.04 (2C), 128.95 (2C), 128.88 (2C), 128.8, 127.5, 127.2, 127.1, 127.1, 127.0, 126.6, 126.6, 126.5, 118.3, 117.3, 68.8, 56.5, 40.0, 35.3, 32.4, 29.5.

NMR spectra showed a mixture of conformers. Due to ^13^C-signal overlapping in the aromatic region, it was not possible to completely assign the major conformer. Therefore, all signals are given.

**HR-MS** (ESI+)**:** *m/z* calcd. for C_21_H_18_N_2_O_2_ [M+H]^+^: 331.1441, found: 331.1441.

**HPLC:** *t*_R_ = 12.5 min.

**LC-MS:** *t*_R_ = 13.9 min.

**Specific rotation:** [α]_D_ = –26.4 °·mL·dm^–1^·g^–1^ (ρ = 0.57; MeOH).

(*S*)-3-benzyl-4-ethyl-3,4-dihydro-1*H*-benzo[1,4]diazepine-2,5-dione (**10m**)

This compound was obtained after purification by preparative HPLC, yielding 6.5 mg of a white solid (22.1 µmol, 5%).

**^1^H-NMR** (600 MHz, CDCl_3_)**:** δ [ppm] = 8.87 (bs, 0.7 H), 8.53 (bs, 0.3 H), 8.09 (dd, *J* = 7.9, 1.5 Hz, 0.7 H), 7.95 (dd, *J* = 7.9, 1.6 Hz, 0.3 H), 7.52 (td, *J* = 7.6, 1.6 Hz, 0.7 H), 7.45 (td, *J* = 7.7, 1.7 Hz, 0.3 H), 7.33 (td, *J* = 7.6, 1.1 Hz, 1.2 H), 7.30–7.26 (m, 1.5 H), 7.26–7.19 (m, 2.3 H), 7.03–6.97 (m, 2.2 H), 6.92–6.86 (m, 0.3 H), 4.37 (dd, *J* = 8.7, 6.1 Hz, 0.3 H), 4.26 (dd, *J* = 9.5, 7.6 Hz, 0.7 H), 4.08 (dq, *J* = 14.4, 7.2 Hz, 0.3 H), 3.74 (dq, *J* = 14.3, 7.2 Hz, 0.7 H), 3.56 (dd, *J* = 14.3, 8.8 Hz, 0.3 H), 3.43–3.34 (m, 0.3 H), 3.24 (dd, *J* = 14.4, 6.1 Hz, 0.3 H), 3.09 (dq, *J* = 14.2, 7.2 Hz, 0.7 H), 2.83 (dd, *J* = 13.7, 6.8 Hz, 0.7 H), 2.72 (dd, *J* = 13.8, 10.2 Hz, 0.7 H), 1.28 (t, *J* = 7.1 Hz, 0.9 H), 0.99 (t, *J* = 7.2 Hz, 2.1 H).

**^13^C-NMR** (151 MHz, CDCl_3_)**:** δ [ppm] = 172.0, 165.8, 135.8, 134.6, 132.9, 131.9, 129.1 (2C), 129.0 (2C), 127.54, 127.46, 125.5, 120.2, 66.7, 46.9, 35.1, 12.9.

NMR spectra showed a mixture of conformers. Only the ^13^C-signals of the major conformer are given.

**HR-MS** (ESI+)**:** *m/z* calcd. for C_18_H_18_N_2_O_2_ [M+Na]^+^: 317.1260, found: 317.1262.

**HPLC:** *t*_R_ = 10.6 min.

**LC-MS:** *t*_R_ = 12.2 min.

**Specific rotation:** [α]_D_ = –168.5 °·mL·dm^–1^·g^–1^ (ρ = 0.22; MeOH).

(*S*)-3-(cyclohexylmethyl)-4-ethyl-3,4-dihydro-1*H*-benzo[1,4]diazepine-2,5-dione (**33**)

This compound was obtained after purification by preparative HPLC, yielding 56.8 mg of an orange oil (0.19 mmol, 50%).

**^1^H-NMR** (600 MHz, CDCl_3_)**:** δ [ppm] = 9.37 (s, 0.6 H), 9.20 (s, 0.4 H), 7.92 (d, *J* = 7.8 Hz, 1 H), 7.46–7.39 (m, 1 H), 7.25 (t, *J* = 7.4 Hz, 0.4 H), 7.20 (t, *J* = 7.6 Hz, 0.6 H), 7.02 (d, *J* = 8.0 Hz, 0.4 H), 6.98 (d, *J* = 7.9 Hz, 0.6 H), 4.14–4.10 (m, 0.6 H), 4.08–4.03 (m, 0.7 H), 4.02–3.95 (m, 0.6 H), 3.45 (dq, *J* = 14.1, 7.1 Hz, 0.6 H), 3.25 (dq, *J* = 13.9, 7.0 Hz, 0.4 H), 2.01–1.96 (m, 0.4 H), 1.80–1.74 (m, 0.4 H), 1.66–1.55 (m, 5 H), 1.42–1.36 (m, 0.6 H), 1.34–1.03 (m, 8 H), 0.94–0.74 (m, 2 H).

**^13^C-NMR** (151 MHz, CDCl_3_)**:** δ [ppm] = 173.2, 165.9, 134.8, 132.5, 131.5, 127.3, 124.9, 120.0, 62.7, 52.7, 46.5, 34.8, 33.2, 26.3, 26.0, 13.4.

NMR spectra showed a mixture of conformers. Only the ^13^C-signals of the major conformer are given.

**HR-MS** (ESI+)**:** *m/z* calcd. for C_18_H_24_N_2_O_2_ [M+Na]^+^: 323.1730, found: 323.1725.

**HPLC:** *t*_R_ = 14.2 min.

**LC-MS:** *t*_R_ = 17.0 min.

**Specific rotation:** [α]_D_ = –40.0 °·mL·dm^–1^·g^–1^ (ρ = 1.30; MeOH).

(*S*)-4-ethyl-3-(4-methoxybenzyl)-3,4-dihydro-1*H*-benzo[1,4]diazepine-2,5-dione (**34**)

This compound was obtained after purification by preparative HPLC, yielding 21.9 mg of a white solid (0.07 mmol, 19%).

**^1^H-NMR** (600 MHz, CDCl_3_)**:** δ [ppm] = 9.02 (s, 0.7 H), 8.67 (s, 0.2 H), 8.07 (d, *J* = 6.8 Hz, 0.7 H), 7.94 (d, *J* = 7.3 Hz, 0.3 H), 7.50 (t, *J* = 7.1 Hz, 0.7 H), 7.44 (t, *J* = 7.8 Hz, 0.3 H), 7.31 (t, *J* = 7.4 Hz, 0.7 H), 7.27 (t, *J* = 7.4 Hz, 0.3 H), 7.14 (d, *J* = 8.4 Hz, 0.4 H), 6.99 (d, *J* = 8.1 Hz, 0.6 H), 6.94–6.76 (m, 4 H), 4.29 (dd, *J* = 8.9, 5.9 Hz, 0.3 H), 4.23–4.19 (m, 0.7 H), 4.06 (dt, *J* = 14.2, 7.2 Hz, 0.5 H), 3.79–3.75 (m, 0.5 H), 3.75 (s, 2.6 H), 3.74 (s, 0.4 H), 3.50 (dd, *J* = 14.2, 8.9 Hz, 0.3 H), 3.39 (dq, *J* = 14.0, 7.0 Hz, 0.3 H), 3.16 (dd, *J* = 14.2, 5.9 Hz, 0.3 H), 3.09 (dq, *J* = 14.2, 7.1 Hz, 0.7 H), 2.77 (dd, *J* = 13.9, 6.9 Hz, 0.7 H), 2.66 (dd, *J* = 13.9, 10.0 Hz, 0.7 H).

**^13^C-NMR** (151 MHz, CDCl_3_)**:** δ [ppm] = 172.3, 165.8, 159.0, 134.7, 132.8, 131.8, 130.1 (2C), 127.7, 127.4, 125.4, 120.2, 114.4 (2C), 66.8, 55.3, 46.8, 34.2, 12.9.

NMR spectra showed a mixture of conformers. Only the ^13^C-signals of the major conformer are given.

**HR-MS** (APCI+)**:** *m/z* calcd. for C_19_H_20_N_2_O_3_ [M+H]^+^: 325.1547, found: 325.1547.

**HPLC:** *t*_R_ = 12.8 min.

**LC-MS:** *t*_R_ = 14.9 min.

**Specific rotation:** [α]_D_ = –94.0 °·mL·dm^–1^·g^–1^ (ρ = 1.43; MeOH).

(*S*)-3-benzyl-4-ethyl-1-hydroxy-3,4-dihydro-1*H*-benzo[1,4]diazepine-2,5-dione (**40**)

This compound was synthesized starting from (*S*)-Fmoc-phenylalanine and isolated as a side product during synthesis of **10m**, yielding 5.0 mg of a white solid (17 µmol, 4%).

**^1^H-NMR** (600 MHz, CDCl_3_)**:** δ [ppm] = 8.05–8.00 (m, 0.8 H), 7.91–7.87 (m, 0.2 H), 7.68–7.60 (m, 1.5 H), 7.55–7.51 (m, 0.5 H), 7.41–7.37 (m, 0.8 H), 7.34–7.31 (m, 0.2 H), 7.29–7.26 (m, 1.5 H), 7.26–7.19 (m, 2 H), 6.97–6.93 (m, 1.5 H), 4.44 (dd, *J* = 10.6, 6.6 Hz, 0.8 H), 4.32 (dd, *J* = 8.4, 6.5 Hz, 0.2 H), 4.06–3.99 (m, 0.2 H), 3.75–3.66 (m, 0.8 H), 3.57 (dd, *J* = 14.5, 8.4 Hz, 0.2 H), 3.37–3.31 (m, 0.2 H), 3.28 (dd, *J* = 14.5, 6.5 Hz, 0.2 H), 3.05–2.97 (m, 0.8 H), 2.74 (dd, *J* = 13.8, 6.6 Hz, 0.8 H), 2.60–2.54 (m, 0.8 H), 1.22 (t, *J* = 7.1 Hz, 0.7 H), 0.96 (t, *J* = 7.2 Hz, 2.3 H).

**^13^C-NMR** (151 MHz, CDCl_3_)**:** δ [ppm] = 168.0, 165.9, 165.7, 165.5, 136.0, 135.3, 135.2, 133.0, 132.6, 131.2, 130.6, 129.11 (2C), 129.05 (2C), 128.9, 127.7, 127.3, 126.6, 126.3, 125.8, 119.2, 118.7, 64.7, 55.9, 46.9, 38.2, 35.0, 32.4, 14.2, 12.8.

NMR spectra showed a mixture of conformers. Due to ^13^C-signal overlapping in the aromatic region, it was not possible to completely assign the major conformer. Therefore, all signals are given.

**HR-MS** (ESI+)**:** *m/z* calcd. for C_18_H_18_N_2_O_3_ [M+H]^+^: 311.1391, found: 311.1400.

**HPLC:** *t*_R_ = 10.2 min.

**LC-MS:** *t*_R_ = 11.8 min.

**Specific rotation:** [α]_D_ = –128.4 °·mL·dm^–1^·g^–1^ (ρ = 0.42; MeOH).

(*S*)-1,2,3,11a-tetrahydro-5*H*-benzopyrrolo[1,2-*a*][1,4]diazepine-5,11(10*H*)-dione (**54**)

This compound was synthesized starting from (*S*)-Fmoc-proline, yielding 36.5 mg (0.17 mmol, 44%) of a white solid.

**^1^H-NMR** (600 MHz, CDCl_3_)**:** δ [ppm] = 9.12 (s, 0.3 H), 9.08 (s, 0.7 H), 7.98 (dt, *J* = 7.9, 1.5 Hz, 1 H), 7.45–7.48 (m, 1 H), 7.23–7.27 (m, 1 H), 7.04–7.07 (m, 1 H), 4.07 (d, *J* = 6.3 Hz, 1 H), 3.78–3.84 (m, 1 H), 3.57–3.64 (m, 1 H), 2.71–2.79 (m, 1 H), 1.99–2.05 (m, 3 H).

**^13^C-NMR** (151 MHz, CDCl_3_)**:** δ [ppm] = 171.5, 165.7, 135.5, 132.6, 131.2, 127.1, 125.2, 121.3, 56.9, 47.5, 24.4, 23.6.

NMR spectra showed a mixture of conformers. Only the ^13^C-signals of the major conformer are given.

**HR-MS** (APCI+)**:** *m/z* calcd. for C_12_H_12_N_2_O_2_ [M+H]^+^: 217.0972, found: 217.0973.

**HPLC:** *t*_R_ = 7.8 min.

**LC-MS:** *t*_R_ = 9.7 min.

**Specific rotation:** [α]_D_ = +161.1 °·mL·dm^–1^·g^–1^ (ρ = 1.80; MeOH).

(*S*)-7,8,9,10-tetrahydrobenzopyrido[1,2-*a*][1,4]diazepine-6,12(5*H*,6a*H*)-dione (**55**)

This compound was synthesized starting from (*S*)-Fmoc-pipecolic acid, yielding 58.0 mg (0.23 mmol, 83%) of a white solid.

**^1^H-NMR** (600 MHz, CDCl_3_)**:** δ [ppm] = 9.05 (bs, 1 H), 7.89 (dd, *J* = 7.9, 1.6 Hz, 1 H), 7.43 (td, *J* = 7.7, 1.6 Hz, 1 H), 7.23 (td, *J* = 7.6, 1.1 Hz, 1 H), 7.01 (dd, *J* = 7.9, 1.1 Hz, 1 H), 4.54–4.47 (m, 1 H), 4.14 (dd, *J* = 6.4, 3.0 Hz, 1 H), 3.01–2.94 (m, 1 H), 2.27–2.21 (m, 1 H), 1.97–1.89 (m, 1 H), 1.84–1.79 (m, 1 H), 1.73–1.65 (m, 2 H), 1.62–1.54 (m, 1 H).

**^13^C-NMR** (151 MHz, CDCl_3_)**:** δ [ppm] = 172.2, 168.7, 136.0, 132.3, 131.3, 127.6, 125.2, 120.6, 51.3, 40.5, 23.2, 22.8, 19.2.

**HR-MS** (APCI+)**:** *m/z* calcd. for C_13_H_14_N_2_O_2_ [M+H]^+^: 231.1128, found: 231.1127.

**HPLC:** *t*_R_ = 7.3 min.

**LC-MS:** *t*_R_ = 8.7 min.

**Specific rotation:** [α]_D_ = +225.0 °·mL·dm^–1^·g^–1^ (ρ = 1.11; MeOH).

(2*R*,11a*S*)-2-hydroxy-1,2,3,11a-tetrahydro-5*H*-benzopyrrolo[1,2-*a*][1,4]diazepine-5,11(10*H*)-dione (**69**)

This compound was synthesized from (2*S*,4*R*)-1-(((9*H*-fluoren-9-yl)methoxy)carbonyl)-4-((*tert*-butyldimethylsilyl)oxy)pyrrolidine-2-carboxylic acid, following the general SPPS and a slightly different cyclization protocol: After the cyclization reaction, the resin was filtered and washed with EtOAc (10 mL). The volatiles were removed *in vacuo* and the residue dissolved in EtOAc (15 mL), before being washed with diluted NH_4_Cl solution (10 mL). The aqueous phase was extracted with EtOAc (15 mL). Combined organic extracts were dried over Na_2_SO_4_, filtered, and the solvent removed under reduced pressure to yield 50 mg of crude **SI-18**.

**HR-MS** (ESI+)**:** *m/z* calcd. for C_18_H_26_N_2_O_3_Si [M+H]^+^: 347.1786, found: 347.1788.

For TBS-deprotection, the residue was dissolved in THF (4 mL), TBAF was added (1 m in THF; 0.36 mL, 0.36 mmol), and the mixture was stirred at room temperature for 2 hours. Afterwards, the volatiles were removed under reduced pressure and the residue purified by preparative HPLC to yield 26.9 mg of a white solid (116 µmol, 64%).

**^1^H-NMR** (600 MHz, DMSO-d_6_)**:** δ [ppm] = 10.55 (bs, 1 H), 7.79 (dd, *J* = 7.9, 1.6 Hz, 1 H), 7.52 (ddd, *J* = 8.1, 7.2, 1.6 Hz, 1 H), 7.23 (ddd, *J* = 7.9, 7.2, 1.2 Hz, 1 H), 7.13 (dd, *J* = 8.2, 1.1 Hz, 1 H), 5.16 (d, *J* = 4.1 Hz, 1 H), 4.33–4.28 (m, 1 H), 4.19 (dd, *J* = 8.0, 5.9 Hz, 1 H), 3.61 (ddd, *J* = 12.1, 3.8, 1.5 Hz, 1 H), 3.47 (dd, *J* = 12.1, 4.8 Hz, 1 H), 2.64–2.59 (m, 1 H), 1.96–1.90 (m, 1 H).

**^13^C-NMR** (151 MHz, DMSO-d_6_)**:** δ [ppm] = 170.4, 165.1, 136.3, 132.2, 130.5, 126.0, 124.0, 121.3, 67.4, 55.2, 54.0, 34.4.

**HR-MS** (ESI+)**:** *m/z* calcd. for C_12_H_12_N_2_O_3_ [M+H]^+^: 233.0921, found: 233.0925.

**HPLC:** *t*_R_ = 7.1 min.

**LC-MS:** *t*_R_ = 7.2 min.

**Specific rotation:** [α]_D_ = +318.3 °·mL·dm^–1^·g^–1^ (ρ = 0.35; MeOH).

**2.2.2 By Acid-induced Cyclization**

For the nitro compounds, acid-induced cyclization was used to obtain the cyclic final products due to base-sensitivity of the nitro group.

The resin was treated with 35% TFA in DCM for 1 hour, filtered, washed with DCM and the procedure repeated one time. Combined filtrates were concentrated under reduced pressure and the residue subjected to preparative HPLC to yield the product.

(*S*)-3-isobutyl-4-methyl-8-nitro-3,4-dihydro-1*H*-benzo[1,4]diazepine-2,5-dione (**9h**)

This compound was obtained as 2.2 mg of a yellow solid (7.6 µmol, 6%).

**^1^H-NMR** (600 MHz, CDCl_3_)**:** δ [ppm] = 8.65 (bs, 0.3 H), 8.49 (bs, 0.6 H), 8.19–8.14 (m, 1 H), 8.10 (dd, *J* = 8.6, 2.1 Hz, 0.6 H), 8.05 (dd, *J* = 8.6, 2.1 Hz, 0.4 H), 7.91 (d, *J* = 2.1 Hz, 0.6 H), 7.86 (d, *J* = 2.1 Hz, 0.4 H), 4.17 (t, *J* = 6.6 Hz, 0.4 H), 3.98 (t, *J* = 6.6 Hz, 0.6 H), 3.35 (s, 1.1 H), 3.11 (s, 1.9 H), 2.02–1.95 (m, 0.6 H), 1.85–1.79 (m, 0.6 H), 1.67–1.55 (m, 1 H), 1.37 (t, *J* = 7.3 Hz, 0.7 H), 0.91 (d, *J* = 6.6 Hz, 3.6 H), 0.89 (d, *J* = 6.6 Hz, 1.2 H), 0.85 (d, *J* = 6.6 Hz, 1.2 H).

**^13^C-NMR** (151 MHz, CDCl_3_)**:** δ [ppm] = 169.9, 167.0, 150.1, 136.5, 133.4, 133.1, 119.7, 115.9, 53.6, 34.8, 29.5, 25.1, 22.6, 22.6.

NMR spectra showed a mixture of conformers.

**HR-MS** (APCI+)**:** *m/z* calcd. for C_14_H_17_N_3_O_4_ [M+H]^+^: 292.1292, found: 292.1294.

**HPLC:** *t*_R_ = 10.1 min.

**LC-MS:** *t*_R_ = 11.9 min.

**Specific rotation:** [α]_D_ = +72.5 °·mL·dm^–1^·g^–1^ (ρ = 0.15; MeOH).

(*S*)-3-benzyl-4-methyl-8-nitro-3,4-dihydro-1*H*-benzo[1,4]diazepine-2,5-dione (**10h**)

This compound was obtained as 13.2 mg of a yellow solid (40.5 µmol, 34%).

**^1^H-NMR** (600 MHz, CDCl_3_)**:** δ [ppm] = 9.62 (bs, 0.3 H), 9.41 (bs, 0.5 H), 8.32 (d, *J* = 8.7 Hz, 0.4 H), 8.19–8.11 (m, 1 H), 8.08 (d, *J* = 8.5 Hz, 0.6 H), 8.00 (d, *J* = 2.2 Hz, 0.4 H), 7.88 (d, *J* = 2.2 Hz, 0.6 H), 7.32–7.20 (m, 4.2 H)*, 7.00 (d, *J* = 7.2 Hz, 0.8 H), 4.36 (dd, *J* = 11.2, 6.1 Hz, 0.4 H), 4.30 (dd, *J* = 8.7, 6.0 Hz, 0.6 H), 3.53 (dd, *J* = 14.2, 8.7 Hz, 0.6 H), 3.24 (dd, *J* = 14.3, 5.9 Hz, 0.6 H), 3.19 (s, 1.8 H), 2.95 (s, 1.2 H), 2.90 (dd, *J* = 13.8, 6.1 Hz, 0.4 H), 2.68 (dd, *J* = 13.8, 11.1 Hz, 0.4 H).

**^13^C-NMR** (151 MHz, CDCl_3_)**:** δ [ppm] = 169.9, 166.8, 150.0, 135.8, 134.9, 133.4, 132.4, 129.2 (2C), 129.0 (2C), 127.4, 119.7, 116.3, 56.7, 32.3, 29.8.

*Signal overlaps with solvent residual peak.

NMR spectra showed a mixture of conformers. Only the ^13^C-signals of the major conformer are given.

**HR-MS** (APCI+)**:** *m/z* calcd. for C_17_H_15_N_3_O_4_ [M+H]^+^: 326.1135, found: 326.1138.

**HPLC:** *t*_R_ = 10.9 min.

**LC-MS:** *t*_R_ = 12.5 min.

**Specific rotation:** [α]_D_ = –5.5 °·mL·dm^–1^·g^–1^ (ρ = 0.15; MeOH).

### **2.2.3 By Final *N^1^*-Methylation**

In a flame-dried 10 mL Schlenk flask, the corresponding benzo[1.4]diazepine-2,5-dione (1.0 eq.) was dissolved in dry THF before addition of sodium hydride (1.8 eq.). After stirring at room temperature for 5 minutes, methyl iodide was added and the mixture stirred at room temperature for 2 hours. The reaction was stopped by addition of 15 mL water. After extraction with EtOAc (3 × 20 mL), the combined organic phases were dried over Na_2_SO_4_, filtered and the solvents removed under reduced pressure. The residue was purified by MPLC or preparative HPLC to yield the *N^1^*-methylated product.

1,4-dimethyl-3,4-dihydro-1*H*-benzo[1,4]diazepine-2,5-dione (**49n**)

This compound was synthesized from 4-methyl-3,4-dihydro-1*H*-benzo[1,4]diazepin-2,5-dione and purified by MPLC, yielding 63.0 mg of a colourless solid (0.31 mmol, 38%).

**^1^H-NMR** (300 MHz, CDCl_3_)**:** δ [ppm] = 7.84 (dd, *J* = 7.8, 1.7 Hz, 1 H), 7.50 (ddd, *J* = 9.1, 7.4, 1.7 Hz, 1 H), 7.27 (td, *J* = 7.5, 1.1 Hz, 1 H), 7.18 (dd, *J* = 8.2, 1.1 Hz, 1 H), 4.05 (d, *J* = 14.5 Hz, 1 H), 3.67 (d, *J* = 14.6 Hz, 1 H), 3.36 (s, 3 H), 3.25 (s, 3 H).

**^13^C-NMR** (75 MHz, CDCl_3_)**:** δ [ppm] = 168.6, 167.5, 141.0, 132.2, 130.8, 128.6, 125.8, 121.0, 53.1, 36.2, 35.0.

**HR-MS** (ESI+)**:** *m/z* calcd. for C_11_H_11_N_2_O_2_ [M+Na]^+^: 227.0791, found: 227.0788.

**HPLC:** *t*_R_ = 6.0 min.

**LC-MS:** *t*_R_ = 7.0 min.

(*S*)-3-isobutyl-1,4-dimethyl-3,4-dihydro-1*H*-benzo[1,4]diazepine-2,5-dione (**9n**)

This compound was synthesized from (S)-3-isobutyl-4-methyl-3,4-dihydro-1*H*-benzo[1,4]diazepin-2,5-dione and purified by preparative HPLC, yielding 46.0 mg of a white solid (0.18 mmol, 67%, quant. brsm).

**^1^H-NMR** (600 MHz, CDCl_3_)**:** δ [ppm] = 7.86 (dd, *J* = 7.8, 1.7 Hz, 0.8 H), 7.82 (dd, *J* = 7.8, 1.7 Hz, 0.2 H)0, 7.52–7.57 (m, 1 H), 7.27–7.32 (m, 1 H), 7.24 (bd, *J* = 7.2 Hz, 0.8 H), 7.17 (d, *J* = 8.2 Hz, 0.2 H), 4.21 (t, *J* = 8.2 Hz, 0.2 H), 4.04 (t, *J* = 7.4 Hz, 0.8 H), 3.41 (s, 2.5 H), 3.40 (s, 0.5 H), 3.08 (s, 2.5 H), 1.90–1.95 (m, 0.8 H), 1.74–1.80 (m, 0.8 H), 1.53–1.61 (m, 0.8 H), 1.41–1.48 (m, 0.2 H), 1.13 (dd, *J* = 8.2, 6.7 Hz, 0.4 H), 0.84 (d, *J* = 6.6 Hz, 5 H), 0.78 (d, *J* = 6.6 Hz, 0.5 H), 0.76 (d, *J* = 6.6 Hz, 0.5 H).

**^13^C-NMR** (151 MHz, CDCl_3_)**:** δ [ppm] = 169.4, 169.1, 141.0, 132.6, 130.5, 128.7, 125.9, 121.2, 54.2, 35.4, 35.1, 29.3, 25.0, 22.6, 22.6.

NMR spectra showed a mixture of conformers. Only the ^13^C-signals of the major conformer are given.

**HR-MS** (ESI+)**:** *m/z* calcd. for C_15_H_20_N_2_O_2_ [M+Na]^+^: 283.1417, found: 283.1415.

**HPLC:** *t*_R_ = 10.3 min.

**LC-MS:** *t*_R_ = 11.6 min.

**Specific rotation:** [α]_D_ = +112.5 °·mL·dm^–1^·g^–1^ (ρ = 1.80; MeOH).

(*S*)-3-benzyl-1,4-dimethyl-3,4-dihydro-1*H*-benzo[1,4]diazepine-2,5-dione (**10n**)

This compound was synthesized from (S)-3-benzyl-4-methyl-3,4-dihydro-1*H*-benzo[1,4]diazepin-2,5-dione and purified by MPLC, yielding 42.4 mg of a white solid (0.14 mmol, 78%).

**^1^H-NMR** (600 MHz, CDCl_3_)**:** δ [ppm] = 7.99 (dd, *J* = 7.8, 1.6 Hz, 0.2 H), 7.88 (dd, *J* = 7.8, 1.6 Hz, 0.8 H), 7.64 (ddd, *J* = 8.4, 7.3, 1.6 Hz, 0.2 H), 7.55 (ddd, *J* = 8.5, 7.3, 1.5 Hz, 0.8 H), 7.41 (td, *J* = 7.6, 1.0 Hz, 0.2 H), 7.33 (td, *J* = 7.6, 1.0 Hz, 0.8 H), 7.16–7.30 (m, 5.6 H), 6.92–6.94 (m, 0.4 H), 4.43 (dd, *J* = 11.2 Hz, 6.2 Hz, 0.2 H), 4.39 (dd, *J* = 8.2, 6.7 Hz, 0.8 H), 3.48 (dd, *J* = 14.6, 8.2 Hz, 0.8 H), 3.45 (s, 0.7 H), 3.41 (s, 2.7 H), 3.22 (dd, *J* = 14.6, 6.7 Hz, 0.8 H), 3.15 (s, 2.3 H), 2.93 (s, 0.7 H), 2.70 (dd, *J* = 13.8, 6.2 Hz, 0.2 H), 2.42 (dd, *J* = 13.8, 11.2 Hz, 0.2 H).

**^13^C-NMR** (151 MHz, CDCl_3_)**:** δ [ppm] = 169.3, 168.4, 140.9, 136.3, 132.8, 130.6, 129.0 (2C), 128.9 (2C), 128.4, 127.1, 126.0, 121.4, 56.9, 35.3, 32.8, 29.5.

**HR-MS** (ESI+)**:** *m/z* calcd. for C_18_H_18_N_2_O_2_ [M+Na]^+^: 317.1261, found: 317.1257.

**HPLC:** *t*_R_ = 11.0 min.

**LC-MS:** *t*_R_ = 12.3 min.

**Specific rotation:** [α]_D_ = +32.8 °·mL·dm^–1^·g^–1^ (ρ = 2.05; MeOH).

**2.2.4 By Macrocyclization**

In a flame-dried 10 mL Schlenk flask, the starting material (1.0 eq.) was dissolved in DCM (50 mL/mmol) and DIPEA (3.0 eq.) was added. Afterwards, HATU (1.8 eq.) was added in small portions and the solution stirred under argon at room temperature for 18 hours. The mixture was poured into 0.1 m HCl_aq_ and extracted with DCM (3 × 10 mL). The combined organic phases were dried over Na_2_SO_4_, filtered and the solvents removed under reduced pressure. The residue was purified by preparative HPLC to yield the desired product.

(*S*)-3-isobutylbenzo[1,4]oxazepine-2,5(1*H*,3*H*)-dione (**79a**)

This compound was synthesized from (*S*)-2-((2-aminobenzoyl)oxy)-4-methylpentanoic acid, yielding 8.7 mg of a white solid (37.3 µmol, 47%).

**^1^H-NMR** (600 MHz, CDCl_3_)**:** δ [ppm] = 8.81 (s, 1 H), 8.01 (dd, *J* = 7.9, 1.6 Hz, 1 H), 7.61 (ddd, *J* = 8.1, 7.4, 1.6 Hz, 1 H), 7.33 (ddd, *J* = 8.0, 7.5, 1.1 Hz, 1 H), 7.08 (dd, *J* = 8.1, 1.1 Hz, 1 H), 4.67 (dd, *J* = 9.4, 4.4 Hz, 1 H), 2.02 (ddd, *J* = 14.4, 9.3, 5.2 Hz, 1 H), 1.98–1.88 (m, 1 H), 1.80 (ddd, *J* = 14.3, 8.6, 4.4 Hz, 1 H), 0.99 (d, *J* = 6.7 Hz, 3 H), 0.90 (d, *J* = 6.6 Hz, 3 H).

**^13^C-NMR** (151 MHz, CDCl_3_)**:** δ [ppm] = 169.8, 167.7, 136.3, 134.7, 133.4, 125.7, 121.9, 120.9, 73.1, 37.5, 23.9, 23.3, 21.9.

**HR-MS** (APCI+)**:** *m/z* calcd. for C_13_H_15_NO_3_ [M+H]^+^: 234.1130, found: 234.1127.

**HPLC:** *t*_R_ = 9.4 min.

**LC-MS:** *t*_R_ = 11.5 min.

**Specific rotation:** [α]_D_ = +266.7 °·mL·dm^–1^·g^–1^ (ρ = 0.44; MeOH).

(*S*)-3-benzylbenzo[1,4]oxazepine-2,5(1*H*,3*H*)-dione (**79b**)

This compound was synthesized from (*S*)-2-((2-aminobenzoyl)oxy)-3-phenylpropanoic acid, yielding 18 mg of a white solid (67 µmol, 71%).

**^1^H-NMR** (600 MHz, CDCl_3_)**:** δ [ppm] = 8.54 (s, 1 H), 7.94 (dd, *J* = 7.9, 1.6 Hz, 1 H), 7.57 (ddd, *J* = 8.1, 7.3, 1.6 Hz, 1 H), 7.34–7.28 (m, 5 H), 7.26–7.23 (m, 1 H), 7.01 (dd, *J* = 8.1, 1.1 Hz, 1 H), 4.79 (dd, *J* = 7.9, 5.5 Hz, 1 H), 3.43 (dd, *J* = 14.6, 5.5 Hz, 1 H), 3.28 (dd, *J* = 14.6, 7.9 Hz, 1 H).

**^13^C-NMR** (151 MHz, CDCl_3_)**:** δ [ppm] = 169.2, 167.3, 136.2, 136.1, 134.7, 133.4, 129.9 (2C), 128.7 (2C), 127.2, 125.6, 121.9, 121.0, 75.6, 35.4.

**HR-MS** (APCI+)**:** *m/z* calcd. for C_16_H_13_NO_3_ [M+H]^+^: 268.0968, found: 268.0970.

**HPLC:** *t*_R_ = 10.2 min.

**LC-MS:** *t*_R_ = 12.2 min.

**Specific rotation:** [α]_D_ = +325.5 °·mL·dm^–1^·g^–1^ (ρ = 0.26; MeOH).

(*S*)-3-isobutyl-4-methyl-3,4-dihydrobenzo[1,4]oxazepine-2,5-dione (**85a**)

This compound was synthesized from *N*-(2-hydroxybenzoyl)-*N*-methyl-*l*-leucine and directly used without further purification due to its instability.

**HR-MS** (ESI–)**:** *m/z* calcd. for C_14_H_17_NO_3_ [M–H]^+^: 246.1136, found: 246.1138.

**HPLC:** *t*_R_ = 10.1 min.

**LC-MS:** *t*_R_ = 12.4 min.

(*S*)-3-benzyl-4-methyl-3,4-dihydrobenzo[1,4]oxazepine-2,5-dione (**85b**)

This compound was synthesized from *N*-(2-hydroxybenzoyl)-*N*-methyl-*l*-phenylalanine and directly used without further purification due to its instability.

**HR-MS** (ESI–)**:** *m/z* calcd. for C_17_H_15_NO_3_ [M–H]^+^: 280.0979, found: 280.0975.

**HPLC:** *t*_R_ = 10.9 min.

**LC-MS:** *t*_R_ = 13.1 min.

**Supplementary Table 1.** Overview over synthesized substrates.

| # | Structure | Yield | # | Structure | Yield |
| --- | --- | --- | --- | --- | --- |
| (*R*)-**9** |  | 30%  (over 7 SPPS steps) | **10i** |  | 3%  (over 4 SPPS steps) |
| **9a** |  | 51%  (over 4 SPPS steps) | **10j** |  | 55%  (over 4 SPPS steps) |
| **9b** |  | 18%  (over 4 SPPS steps) | **10k** |  | 10%  (over 4 SPPS steps) |
| **9c** |  | 26%  (over 4 SPPS steps) | **10l** |  | 45%  (over 4 SPPS steps) |
| **9d** |  | 36%  (over 4 SPPS steps) | **10m** |  | 5%  (over 7 SPPS steps) |
| **9e** |  | 18%  (over 4 SPPS steps) | **33** |  | 50%  (over 7 SPPS steps) |
| **9f** |  | 24%  (over 4 SPPS steps) | **34** |  | 19%  (over 7 SPPS steps) |
| **9g** |  | 33%  (over 4 SPPS steps) | **40** |  | 4%  (over 7 SPPS steps) |
| **9i** |  | 5%  (over 4 SPPS steps) | **54** |  | 44%  (over 4 SPPS steps) |
| **9j** |  | 68%  (over 4 SPPS steps) | **55** |  | 83%  (over 4 SPPS steps) |
| **9k** |  | 12%  (over 4 SPPS steps) | **69** |  | 64%  (over 4 SPPS and 1 deprotection step) |
| **9l** |  | 33%  (over 4 SPPS steps) | **9h** |  | 6%  (over 4 SPPS steps) |
| **9m** |  | 6%  (over 7 SPPS steps) | **10h** |  | 34%  (over 4 SPPS steps) |
| (*R*)-**10** |  | 34%  (over 7 SPPS steps) | **49n** |  | 38%  (1 step) |
| **10a** |  | 27%  (over 4 SPPS steps) | **9n** |  | 67%  (1 step) |
| **10b** |  | 18%  (over 4 SPPS steps) | **10n** |  | 78%  (1 step) |
| **10c** |  | 49%  (over 4 SPPS steps) | **79a** |  | 4%  (over 5 steps) |
| **10d** |  | 21%  (over 4 SPPS steps) | **79b** |  | 18%  (over 5 steps) |
| **10e** |  | 7%  (over 4 SPPS steps) | **85a** |  | n/a  (generated *in situ*) |
| **10f** |  | 24%  (over 4 SPPS steps) | **85b** |  | n/a  (generated *in situ*) |
| **10g** |  | 35%  (over 4 SPPS steps) |  |  |  |

**2.3 Synthesis of Standards**

2-amino-4-fluoro-*N*-methylbenzamide (**SI-19**)

In a 100 mL round-bottomed flask, 0.5 g 7-fluoroisatoic anhydride (2.8 mmol, 1.0 eq.) were suspended in water (7 mL), then 0.68 mL methylamine (33% solution in EtOH, 5.5 mmol, 2.0 eq.) were added slowly over 5 minutes. The resulting mixture was stirred at room temperature for 5 hours, before being extracted with EtOAc (2 × 30 mL). Combined organic extracts were washed with water and brine (20 mL each), dried over Na_2_SO_4_, filtered and the solvent removed under reduced pressure. The desired product was obtained as 0.44 g of a yellowish oil (2.6 mmol, 94%), which solidified upon standing and was used without further purification.

**^1^H-NMR** (600 MHz, CDCl_3_)**:** δ [ppm] = 7.27 (dd, *J* = 8.6, 6.2 Hz, 1 H), 6.36–6.30 (m, 2 H), 5.98 (bs, 1 H), 5.72 (bs, 2 H), 2.95 (d, *J* = 4.8 Hz, 3 H).

**^13^C-NMR** (151 MHz, CDCl_3_)**:** δ [ppm] = 169.5, 165.4 (d, *J* = 249 Hz), 151.1 (d, *J* = 12.1 Hz), 129.3 (d, *J* = 11.0 Hz), 112.6 (d, *J* = 2.3 Hz), 104.1 (d, *J* = 22.6 Hz), 103.1 (d, *J* = 24.3 Hz), 26.7.

**^19^F-NMR** (282 MHz, CDCl_3_)**:** δ [ppm] = –108.34 (s).

**HR-MS** (ESI+)**:** *m/z* calcd. for C_8_H_9_FN_2_O [M+H]^+^: 169.0772; found: 169.0771.

7-fluoro-3-methylquinazoline-2,4(1*H*,3*H*)-dione (**22c**)

In a 100 mL round-bottomed flask, 0.30 g 2-amino-4-fluoro-*N*-methylbenzamide (1.78 mmol, 1.0 eq.) was dissolved in 20 mL DCM, and 1.56 mL DIPEA (1.15 g, 8.92 mmol, 5.0 eq.) were added. After stirring the solution for 10 minutes, 0.87 g 1,1'-carbonyldiimidazole (5.34 mmol, 3.0 eq.) were added and the mixture stirred at 50 °C for 14 hours. After dilution with DCM (10 mL) the reaction was quenched by addition of water (15 mL) and the organic phase was separated. The aqueous phase was extracted with EtOAc (2 × 15 mL) and combined organic extracts were washed with 20 mL brine, dried over Na_2_SO_4_, filtered and the solvents removed under reduced pressure. The residue was purified by column chromatography (pentane/EtOAc = 2/1), yielding 136 mg of a white solid (700 µmol, 39%).

**^1^H-NMR** (600 MHz, DMSO-d_6_)**:** δ [ppm] = 11.56 (s, 1 H), 7.98 (dd, *J* = 8.8, 6.1 Hz, 1 H), 7.05 (td, *J* = 8.7, 2.5 Hz, 1 H), 6.90 (dd, *J* = 9.8, 2.4 Hz, 1 H), 3.23 (s, 3 H).

**^13^C-NMR** (151 MHz, DMSO-d_6_)**:** δ [ppm] = 165.7 (d, *J* = 251 Hz), 161.4, 150.4, 141.3 (d, *J* = 12.7 Hz), 130.6 (d, *J* = 11.6 Hz), 110.8 (d, *J* = 2.0 Hz), 110.5 (d, *J* = 23.1 Hz), 101.2 (d, *J* = 26.0 Hz), 27.0.

**^19^F-NMR** (282 MHz, DMSO-d_6_)**:** δ [ppm] = –104.06 (s).

**HR-MS** (ESI+)**:** *m/z* calcd. for C_9_H_7_FN_2_O_2_ [M–H]^–^: 193.0418, found: 193.0418.

***R*_f_:** 0.38 (pentane/EtOAc = 2/1).

2-amino-*N*-(4-hydroxybutyl)benzamide (**SI-22**)

In a 100 mL round-bottomed flask, 3.0 g isatoic anhydride (18.4 mmol, 1.0 eq.) were suspended in water (25 mL), then 3.38 mL 4-aminobutan-1-ol (3.28 g, 36.8 mmol, 2.0 eq.) were added slowly over 20 minutes. The resulting mixture was stirred at room temperature for 5 hours, before being extracted with EtOAc (2 × 30 mL). Combined organic extracts were washed with water and brine (20 mL each), dried over Na_2_SO_4_, filtered and the solvent removed under reduced pressure. The desired product was obtained as 2.96 g of a beige solid (14.2 mmol, 77%), which was used without further purification.

**^1^H-NMR** (600 MHz, DMSO-d_6_)**:** δ [ppm] = 8.18 (t, *J* = 5.7 Hz, 1 H), 7.44 (dd, *J* = 7.9, 1.5 Hz, 1 H), 7.11 (ddd, *J* = 8.4, 7.1, 1.5 Hz, 1 H), 6.67 (dd, *J* = 8.4, 1.2 Hz, 1 H), 6.49 (ddd, *J* = 8.0, 7.1, 1.2 Hz, 1 H), 6.36 (s, 2 H), 4.41 (t, *J* = 5.1 Hz, 1 H), 3.41 (td, *J* = 6.4, 5.1 Hz, 2 H), 3.19 (td, *J* = 7.0, 5.7 Hz, 2 H), 1.55–1.49 (m, 2 H), 1.47–1.41 (m, 2 H).

**^13^C-NMR** (151 MHz, DMSO-d_6_)**:** δ [ppm] = 168.8, 149.6, 131.5, 128.0, 116.3, 115.1, 114.5, 60.5, 38.7, 30.1, 25.9.

**HR-MS** (ESI+)**:** *m/z* calcd. for C_11_H_16_N_2_O_2_ [M+H]^+^: 209.1285, found: 209.1281.

2-amino-*N*-(4-((*tert*-butyldimethylsilyl)oxy)butyl)benzamide (**SI-23**)

In a flame-dried 100 mL Schlenk flask, 1.0 g 2-amino-*N*-(4-hydroxybutyl)benzamide (4.8 mmol, 1.0 eq.) was dissolved in 18 mL DCM. Afterwards, 0.94 g TBSCl (6.2 mmol, 1.3 eq.) and 0.98 g imidazole (14.4 mmol, 3.0 eq.) were added and the resulting suspension was stirred at room temperature for 17 hours. The mixture was diluted with DCM (15 mL) and quenched by addition of water (15 mL). The organic phase was separated, and the aqueous phase was extracted with DCM (1 × 20 mL). Combined organic phases were washed with 20 mL brine, dried over Na_2_SO_4_, filtered and the solvents removed under reduced pressure. The residue was purified by column chromatography (pentane/EtOAc = 4/1), yielding 1.39 g of a yellowish oil (4.3 mmol, 90%) that solidified upon standing.

**^1^H-NMR** (600 MHz, CDCl_3_)**:** δ [ppm] = 7.31 (dd, *J* = 7.9, 1.5 Hz, 1 H), 7.21 (ddd, *J* = 8.4, 7.2, 1.5 Hz, 1 H), 6.72 (dd, *J* = 8.2, 1.2 Hz, 1 H), 6.67 (ddd, *J* = 8.0, 7.1, 1.2 Hz, 1 H), 6.28 (bs, 1 H), 5.81 (bs, 2 H), 3.68 (t, *J* = 6.0 Hz, 2 H), 3.44 (td, *J* = 6.8, 5.6 Hz, 2 H), 1.71–1.65 (m, 2 H), 1.65–1.59 (m, 2 H), 0.89 (s, 9 H), 0.06 (s, 6 H).

**^13^C-NMR** (151 MHz, CDCl_3_)**:** δ [ppm] = 169.3, 148.1, 132.3, 127.2, 117.8, 117.2, 116.9, 62.9, 39.6, 30.4, 26.3, 26.1 (3C), 18.5, –5.1 (2C).

**HR-MS** (ESI+)**:** *m/z* calcd. for C_17_H_30_N_2_O_2_Si [M+H]^+^: 323.2150, found: 323.2144.

***R*_f_:** 0.40 (pentane/EtOAc = 4/1).

3-(4-((*tert*-butyldimethylsilyl)oxy)butyl)quinazoline-2,4(1*H*,3*H*)-dione (**SI-24**)

In a 50 mL round-bottomed flask, 0.50 g 2-amino-*N*-(4-((*tert*-butyldimethylsilyl)oxy)butyl)benzamide (1.55 mmol, 1.0 eq.) was dissolved in 18 mL DCM, and 1.37 mL DIPEA (1.00 g, 7.75 mmol, 5.0 eq.) were added. After stirring the solution for 10 minutes, 0.75 g 1,1'-carbonyldiimidazole (4.63 mmol, 3.0 eq.) were added and the mixture stirred at 50 °C for 14 hours. After dilution with DCM (10 mL) the reaction was quenched by addition of water (15 mL) and the organic phase was separated. The aqueous phase was extracted with DCM (1 × 15 mL) and combined organic extracts were washed with 15 mL brine, dried over Na_2_SO_4_, filtered and the solvent removed under reduced pressure. The residue was purified by column chromatography (pentane/EtOAc = 3/1), yielding 0.49 g of a white solid (1.40 mmol, 91%).

**^1^H-NMR** (300 MHz, CDCl_3_)**:** δ [ppm] = 11.09 (s, 1 H), 8.11 (dd, *J* = 8.0, 1.5 Hz, 1 H), 7.59 (ddd, *J* = 8.5, 7.4, 1.5 Hz, 1 H), 7.25–7.13 (m, 2 H), 4.13 (bt, *J* = 7.3 Hz, 2 H), 3.66 (t, *J* = 6.3 Hz, 2 H), 1.86–1.72 (m, 2 H), 1.70–1.56 (m, 2 H), 0.86 (s, 9 H), 0.03 (s, 6 H).

**^13^C-NMR** (75 MHz, CDCl_3_)**:** δ [ppm] = 162.5, 152.7, 138.9, 135.0, 128.4, 123.4, 115.3, 114.7, 62.9, 41.0, 30.3, 26.1 (3C), 24.6, 18.4, –5.2 (2C).

**HR-MS** (ESI+)**:** *m/z* calcd. for C_18_H_28_N_2_O_3_Si [M+Na]^+^: 371.1762, found: 371.1757.

***R*_f_:** 0.52 (pentane/EtOAc = 3/1).

3-(4-hydroxybutyl)quinazoline-2,4(1*H*,3*H*)-dione (**SI-25**)

In a 50 mL round-bottomed flask, 0.49 g 3-(4-((*tert*-butyldimethylsilyl)oxy)butyl)quinazoline-2,4(1*H*,3*H*)-dione (1.37 mmol, 1.0 eq.) was dissolved in 4 mL THF, then 4.13 mL TBAF (1 m in THF, 4.13 mmol, 3.0 eq.) were added and the solution was stirred at room temperature for 4 hours. The mixture was diluted with DCM (30 mL) and quenched by addition of water (10 mL). After separation of the organic phase, the aqueous phase was extracted with DCM (1 × 20 mL) and combined organic extracts were washed with brine (20 mL), dried over Na_2_SO_4_, filtered and the solvents removed under reduced pressure. The residue was purified by column chromatography (EtOAc), yielding 0.20 g of a white solid (0.85 mmol, 62%).

**^1^H-NMR** (600 MHz, DMSO-d_6_)**:** δ [ppm] = 11.42 (s, 1 H), 7.92 (dd, *J* = 7.9, 1.5 Hz, 1 H), 7.64 (ddd, *J* = 8.4, 7.2, 1.5 Hz, 1 H), 7.19 (ddd, *J* = 8.1, 7.3, 1.0 Hz, 1 H), 7.17 (bd, *J* = 8.2 Hz, 1 H), 4.41 (t, *J* = 5.2 Hz, 1 H), 3.89 (bt, *J* = 7.4 Hz, 2 H), 3.40 (td, *J* = 6.4, 4.9 Hz, 2 H), 1.63–1.56 (m, 2 H), 1.45–1.39 (m, 2 H).

**^13^C-NMR** (151 MHz, DMSO-d_6_)**:** δ [ppm] = 161.9, 150.2, 139.4, 135.0, 127.4, 122.5, 115.1, 113.8, 60.5, 31.0, 30.0, 24.5.

**HR-MS** (ESI+)**:** *m/z* calcd. for C_12_H_14_N_2_O_3_ [M+Na]^+^: 257.0897, found: 257.0898.

***R*_f_:** 0.49 (EtOAc).

4-(2,4-dioxo-1,4-dihydroquinazolin-3(2*H*)-yl)butanal (**76**)

In a flame-dried 25 mL Schlenk flask, 100 mg 3-(4-hydroxybutyl)quinazoline-2,4(1*H*,3*H*)-dione (0.43 mmol, 1.0 eq.) were dissolved in 7 mL THF. 270 mg DMP (0.64 mmol, 1.5 eq.) were added at 0 °C and the mixture was stirred at room temperature for 75 minutes, before dilution with DCM (15 mL) and quenching of the reaction by addition of saturated NaHCO_3_ solution (15 mL). After separation of the organic phase, the aqueous phase was extracted with DCM (1 × 15 mL) and combined organic extracts were washed with brine (25 mL), dried over Na_2_SO_4_, filtered and the solvents removed under reduced pressure. The residue was purified by column chromatography (pentane/EtOAc = 2/3), yielding the desired aldehyde as 79 mg of a white solid (0.34 mmol, 79%). A small amount was purified by preparative HPLC to remove remaining traces of oxidant.

**^1^H-NMR** (600 MHz, DMSO-d_6_)**:** δ [ppm] = 11.43 (s, 1 H), 9.64 (t, *J* = 1.4 Hz, 1 H), 7.92 (dd, *J* = 7.8, 1.5 Hz, 1 H), 7.65 (ddd, 8.6, 7.2, 1.5 Hz, 1 H), 7.20 (ddd, *J* = 8.1, 7.1, 1.0 Hz, 1 H), 7.17 (bd, *J* = 8.2 Hz, 1 H), 3.91 (t, *J* = 7.0 Hz, 2 H), 2.51–2.47* (m, 2 H), 1.84 (p, *J* = 7.1 Hz, 2 H).

**^13^C-NMR** (151 MHz, DMSO-d_6_)**:** δ [ppm] = 202.6, 162.1, 150.2, 139.4, 135.0, 127.4, 122.5, 115.1, 113.8, 40.4, 39.3*, 20.1.

*: Signals overlap with solvent peaks.

**HR-MS** (ESI+)**:** *m/z* calcd. for C_12_H_12_N_2_O_3_ [M+Na]^+^: 255.0741, found: 255.0741. (not observed as main signal, this was corresponding to C_13_H_16_N_2_O_4_, hence a MeOH adduct).

***R*_f_:** 0.42 (pentane/EtOAc = 2/3).

# **3. Enzymatic Assays**

## **3.1 Procedure**

Analytical enzymatic assays were carried out in a total volume of 120–350 µL, containing 1 mm of the substrate (dissolved in DMF: 100 mm), 50 µm (5 mol-%) of purified AsqJ, 2.5 mm α-ketoglutarate, 4 mm ascorbic acid, 100 µm iron sulfate and 5% (v/v) DMF. The reaction buffer contained 50 mm TRIS HCl at a pH of 7.4. After incubation for 5–6 h (8 h max.) with shaking at 300 rpm, the enzyme was precipitated by addition of TFA (1 µL TFA / 40 µL assay) and removed by centrifugation for 5 min at 9700 × *g*. The supernatant (20 µL) was analysed by HPLC at 220 nm with coupled MS (cf. chapter 1.1). The chromatograms shown are not representing quantitative data/conversion. Retention times between different substrates may not be comparable because of the use of different columns.

## **3.2 HPLC Data of Qualitative Assays**

2-isobutyl-3-methylquinazolin-4(3*H*)-one (**6**)^2^

**6**

[min]


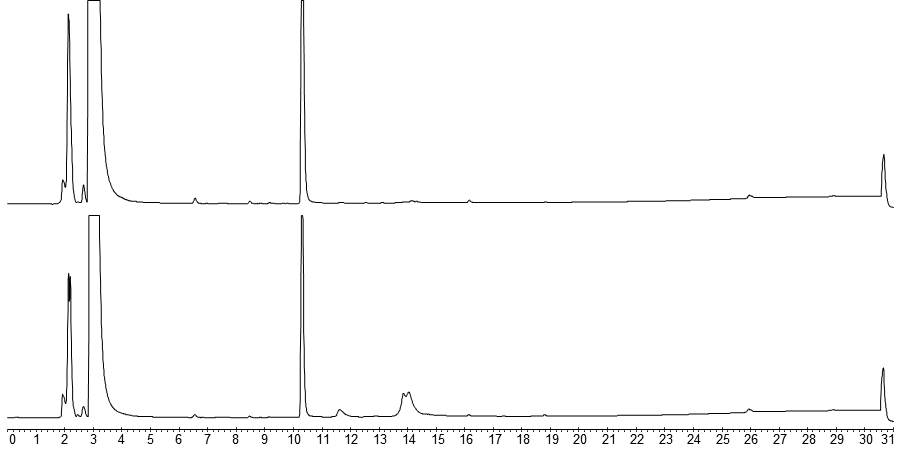


AsqJ

**Supplementary Figure 2.** HPLC chromatograms of negative control (top) and AsqJ-assay (bottom) with 2-isobutyl-3-methylquinazolin-4(3*H*)-one (**6**) as substrate.

(*R*)-3-isobutyl-4-methyl-3,4-dihydro-1*H*-benzo[1,4]diazepine-2,5-dione ((*R*)-**9**)

(*R*)-**9**

[min]


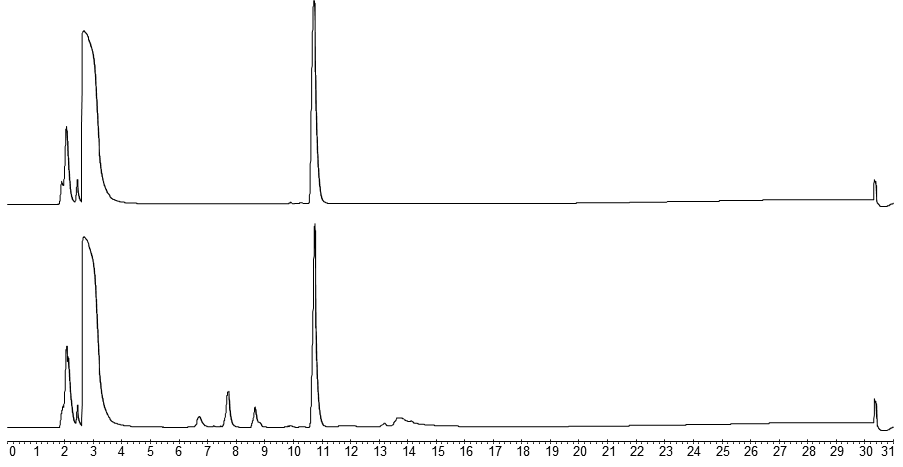


**22**

**6**

**Supplementary Figure 3**. HPLC chromatograms of negative control (top) and AsqJ-assay (bottom) with (*R*)-3-isobutyl-4-methyl-3,4-dihydro-1*H*-benzo[1,4]diazepine-2,5-dione ((*R*)-**9**) as substrate.

(*S*)-6-fluoro-3-isobutyl-4-methyl-3,4-dihydro-1*H*-benzo[1,4]diazepine-2,5-dione (**9a**)

**9a**

[min]


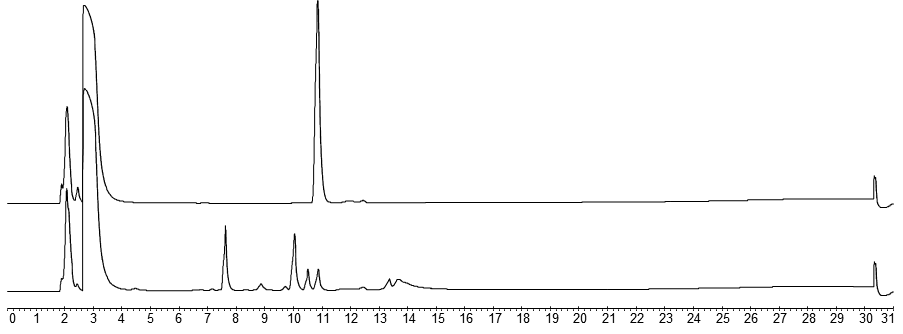


**21a**

**22a**

**SI-26**

**Supplementary Figure 4.** HPLC chromatograms of negative control (top) and AsqJ-assay (bottom) with (*S*)-6-fluoro-3-isobutyl-4-methyl-3,4-dihydro-1*H*-benzo[1,4]diazepine-2,5-dione (**9a**) as substrate.

(*S*)-7-fluoro-3-isobutyl-4-methyl-3,4-dihydro-1*H*-benzo[1,4]diazepine-2,5-dione (**9b**)

**22b**

[min]


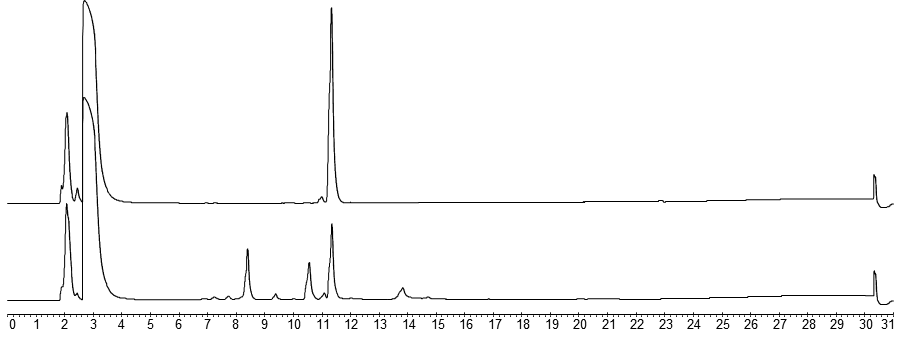


**9b**

**21b**

**Supplementary Figure 5.** HPLC chromatograms of negative control (top) and AsqJ-assay (bottom) with (*S*)-7-fluoro-3-isobutyl-4-methyl-3,4-dihydro-1*H*-benzo[1,4]diazepine-2,5-dione (**9b**) as substrate.

(*S*)-8-fluoro-3-isobutyl-4-methyl-3,4-dihydro-1*H*-benzo[1,4]diazepine-2,5-dione (**9c**)

**9c**


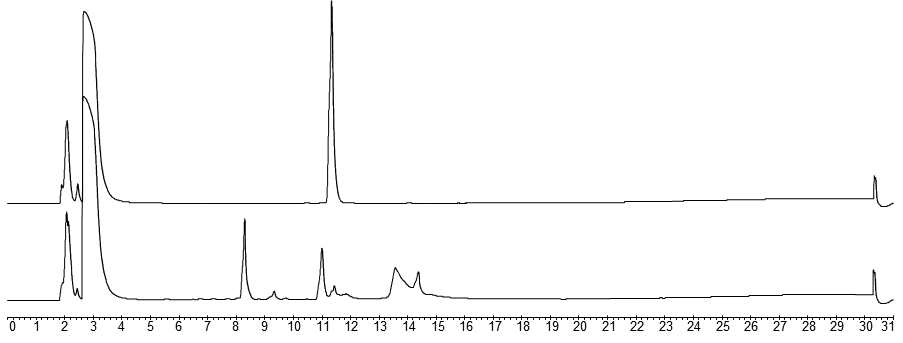


[min]

**22c**

**21c**

AsqJ

**Supplementary Figure 6.** HPLC chromatograms of negative control (top) and AsqJ-assay (bottom) with (*S*)-8-fluoro-3-isobutyl-4-methyl-3,4-dihydro-1*H*-benzo[1,4]diazepine-2,5-dione (**9c**) as substrate.

(*S*)-9-fluoro-3-isobutyl-4-methyl-3,4-dihydro-1*H*-benzo[1,4]diazepine-2,5-dione (**9d**)

**9d**

[min]


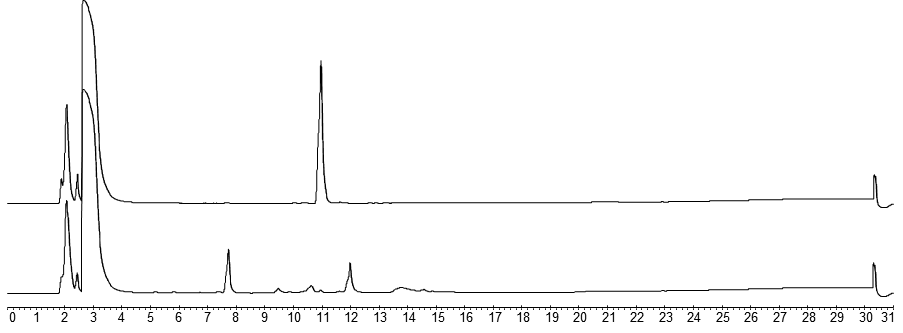


**22d**

**21d**

**Supplementary Figure 7.** HPLC chromatograms of negative control (top) and AsqJ-assay (bottom) with (*S*)-9-fluoro-3-isobutyl-4-methyl-3,4-dihydro-1*H*-benzo[1,4]diazepine-2,5-dione (**9d**) as substrate.

(*S*)-6-chloro-3-isobutyl-4-methyl-3,4-dihydro-1*H*-benzo[1,4]diazepine-2,5-dione (**9e**)

**9e**

[min]


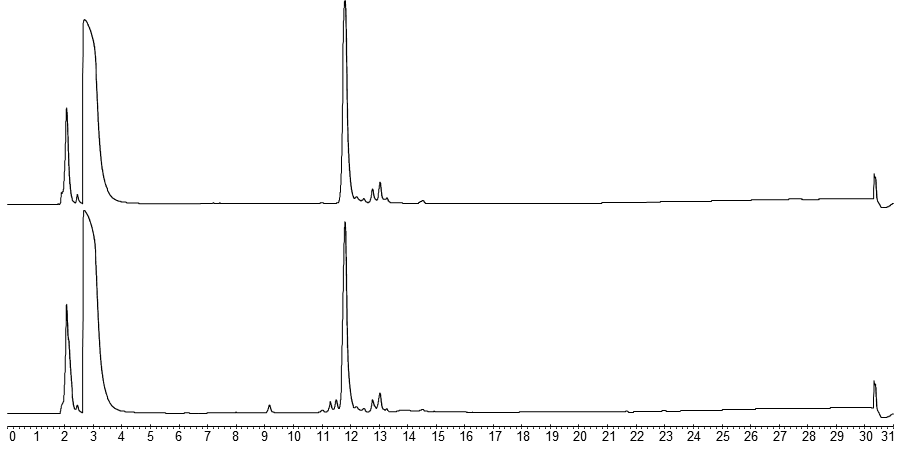


**21e**

**Supplementary Figure 8.** HPLC chromatograms of negative control (top) and AsqJ-assay (bottom) with (*S*)-6-chloro-3-isobutyl-4-methyl-3,4-dihydro-1*H*-benzo[1,4]diazepine-2,5-dione (**9e**) as substrate.

(*S*)-7-chloro-3-isobutyl-4-methyl-3,4-dihydro-1*H*-benzo[1,4]diazepine-2,5-dione (**9f**)

**9f**


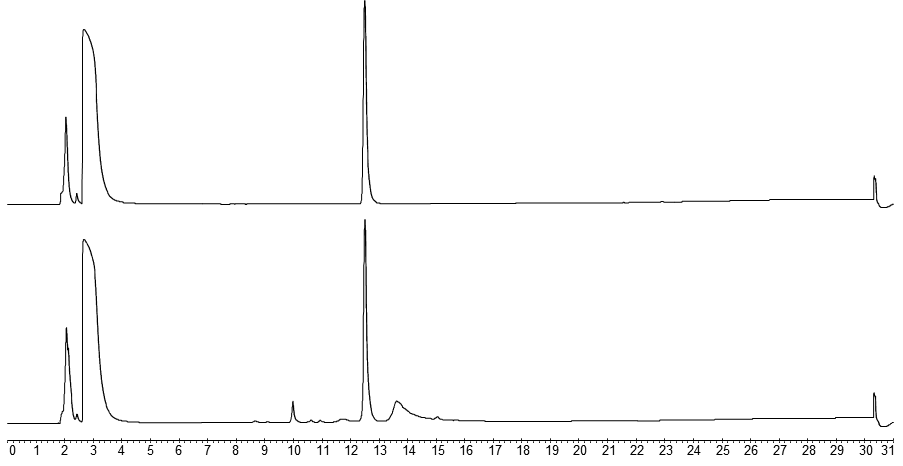


AsqJ

[min]

**Supplementary Figure 9.** HPLC chromatograms of negative control (top) and AsqJ-assay (bottom) with (*S*)-7-chloro-3-isobutyl-4-methyl-3,4-dihydro-1*H*-benzo[1,4]diazepine-2,5-dione (**9f**) as substrate.

(*S*)-8-chloro-3-isobutyl-4-methyl-3,4-dihydro-1*H*-benzo[1,4]diazepine-2,5-dione (**9g**)

**9g**


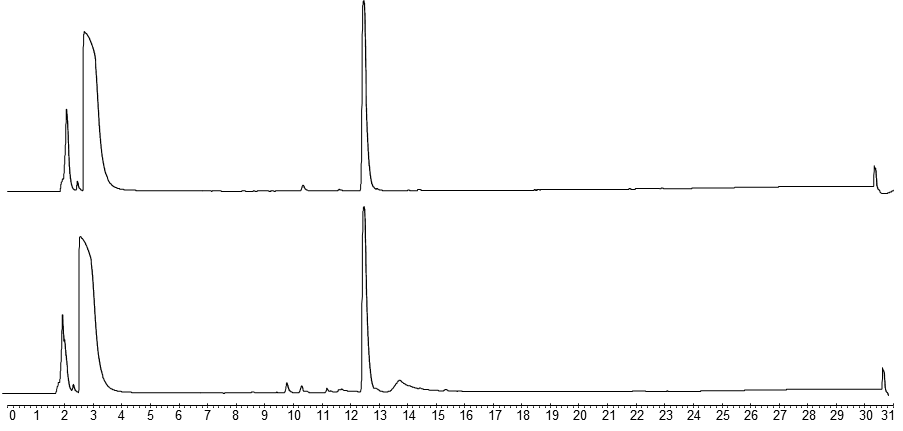


[min]

**Supplementary Figure 10.** HPLC chromatograms of negative control (top) and AsqJ-assay (bottom) with (*S*)-8-chloro-3-isobutyl-4-methyl-3,4-dihydro-1*H*-benzo[1,4]diazepine-2,5-dione (**9g**) as substrate.

(*S*)-3-isobutyl-4,6-dimethyl-3,4-dihydro-1*H*-benzo[1,4]diazepine-2,5-dione (**9i**)

[min]


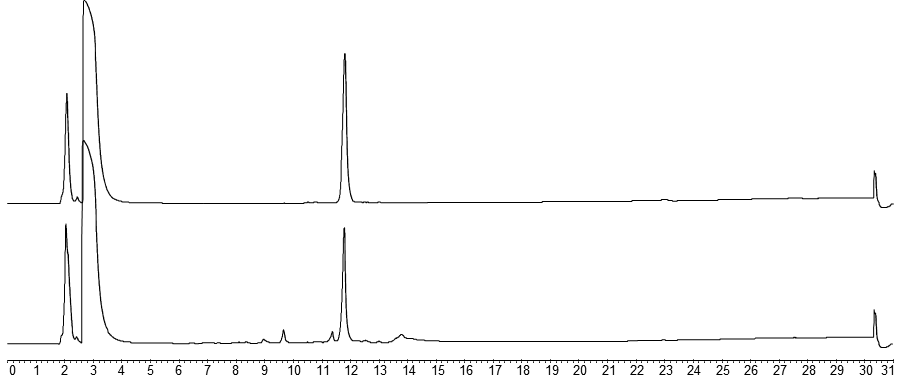


**9i**

**SI-27**

**21i**

**Supplementary Figure 11.** HPLC chromatograms of negative control (top) and AsqJ-assay (bottom) with (*S*)-3-isobutyl-4,6-dimethyl-3,4-dihydro-1*H*-benzo[1,4]diazepine-2,5-dione (**9i**) as substrate.

(*S*)-3-isobutyl-4,7-dimethyl-3,4-dihydro-1*H*-benzo[1,4]diazepine-2,5-dione (**9j**)

**9j**


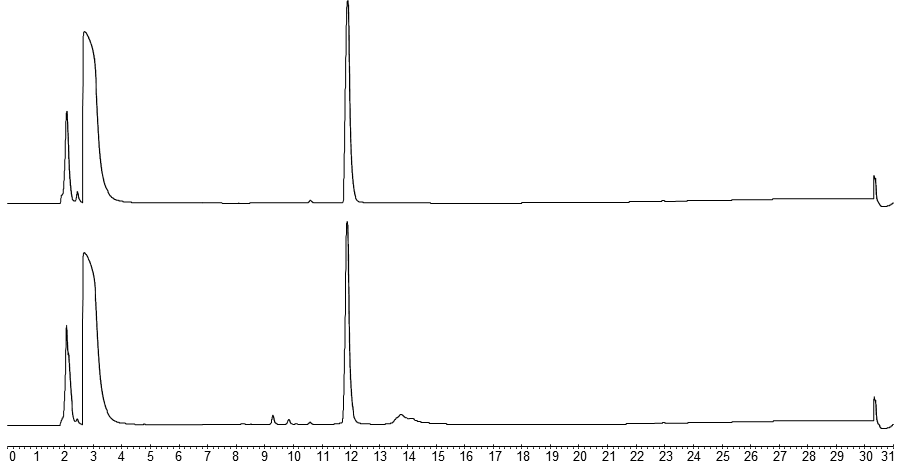


**22j**

**21j**

[min]

**Supplementary Figure 12.** HPLC chromatograms of negative control (top) and AsqJ-assay (bottom) with (*S*)-3-isobutyl-4,7-dimethyl-3,4-dihydro-1*H*-benzo[1,4]diazepine-2,5-dione (**9j**) as substrate.


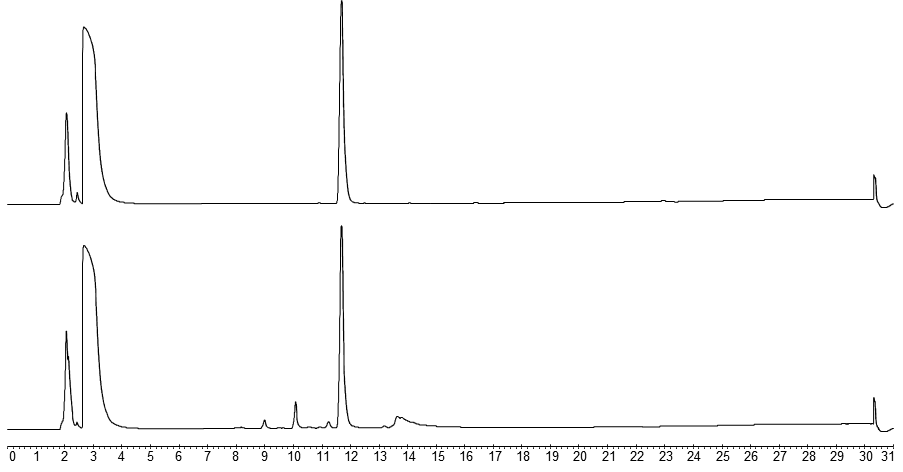
(*S*)-3-isobutyl-4,9-dimethyl-3,4-dihydro-1*H*-benzo[1,4]diazepine-2,5-dione (**9k**)

**9k**

**22k**

[min]

**Supplementary Figure 13.** HPLC chromatograms of negative control (top) and AsqJ-assay (bottom) with (*S*)-3-isobutyl-4,9-dimethyl-3,4-dihydro-1*H*-benzo[1,4]diazepine-2,5-dione (**9k**) as substrate.

(*S*)-3-isobutyl-4-methyl-3,4-dihydro-1*H*-naphtho[2,3-*e*][1,4]diazepine-2,5-dione (**9l**)

This assay was performed with 8% (v/v) DMF.

**9l**

[min]


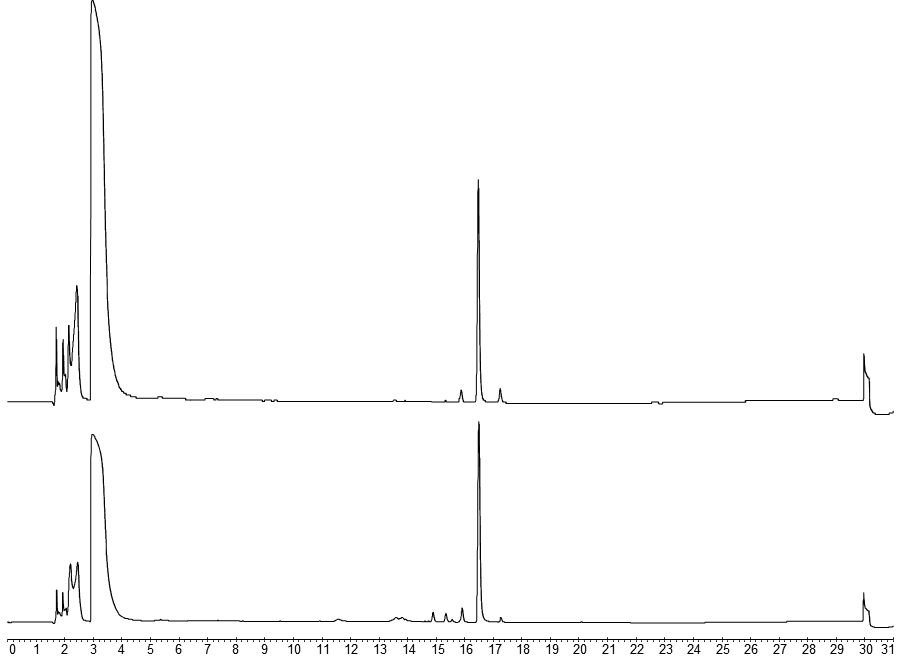


**Supplementary Figure 14.** HPLC chromatograms of negative control (top) and AsqJ-assay (bottom) with (*S*)-3-isobutyl-4-methyl-3,4-dihydro-1*H*-naphtho[2,3-*e*][1,4]diazepine-2,5-dione (**9l**) as substrate. Minor peaks in negative control result from inseparable impurities of substrate synthesis.

(*S*)-4-ethyl-3-isobutyl-3,4-dihydro-1*H*-benzo[1,4]diazepine-2,5-dione (**9m**)

**9m**


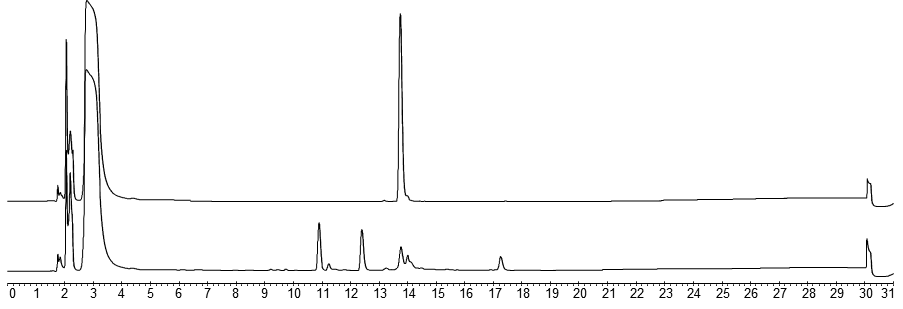


[min]

**41**

**43**

*

**Supplementary Figure 15.** HPLC chromatograms of negative control (top) and AsqJ-assay (bottom) with (*S*)-4-ethyl-3-isobutyl-3,4-dihydro-1*H*-benzo[1,4]diazepine-2,5-dione (**9m**) as substrate. *: Unidentified side-product.

(*R*)-3-benzyl-4-methyl-3,4-dihydro-1*H*-benzo[1,4]diazepine-2,5-dione ((*R*)-**10**)

(*R*)-**10**

[min]


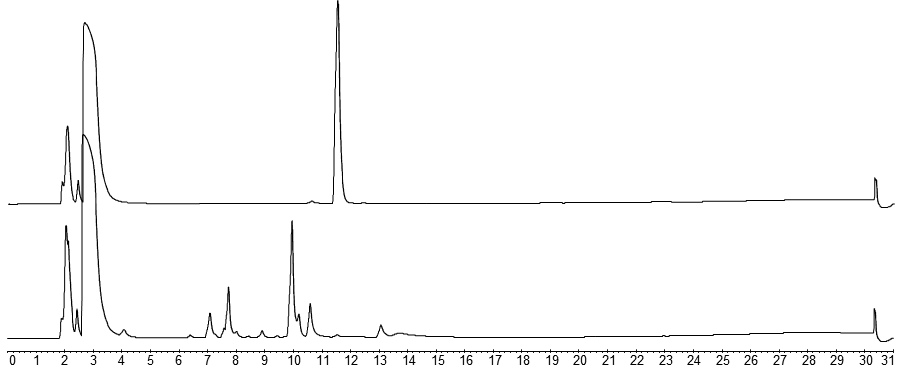


**

**30**

*

**28**

*

**Supplementary Figure 16.** HPLC chromatograms of negative control (top) and AsqJ-assay (bottom) with (*R*)-3-benzyl-4-methyl-3,4-dihydro-1*H*-benzo[1,4]diazepine-2,5-dione ((*R*)-**10**) as substrate. *: Unidentified side-products. **: Isolation and identification attempted, but not successful; ESI-HRMS-proposed formula: C_11_H_12_N_2_O_4_.

(*S*)-3-benzyl-6-fluoro-4-methyl-3,4-dihydro-1*H*-benzo[1,4]diazepine-2,5-dione (**10a**)

**10a**

[min]


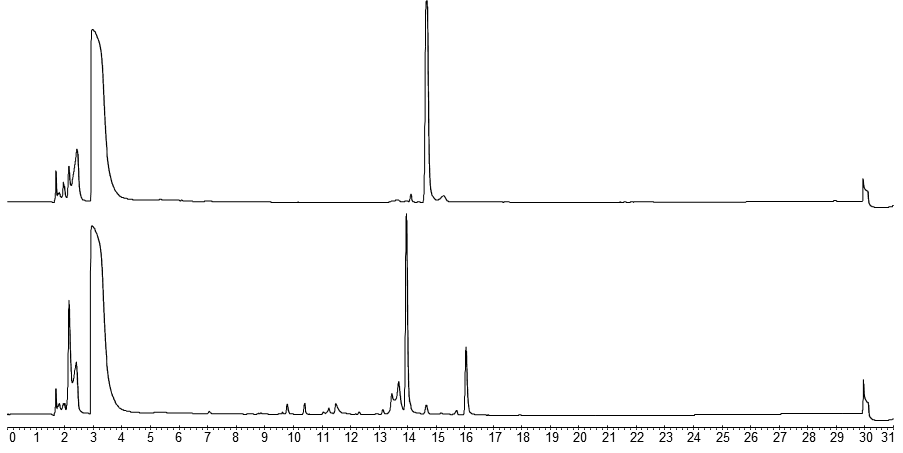


**30a**

**28a**

**Supplementary Figure 17.** HPLC chromatograms of negative control (top) and AsqJ-assay (bottom) with (*S*)-3-benzyl-6-fluoro-4-methyl-3,4-dihydro-1*H*-benzo[1,4]diazepine-2,5-dione (**10a**) as substrate.

(*S*)-3-benzyl-7-fluoro-4-methyl-3,4-dihydro-1*H*-benzo[1,4]diazepine-2,5-dione (**10b**)

**10b**

[min]


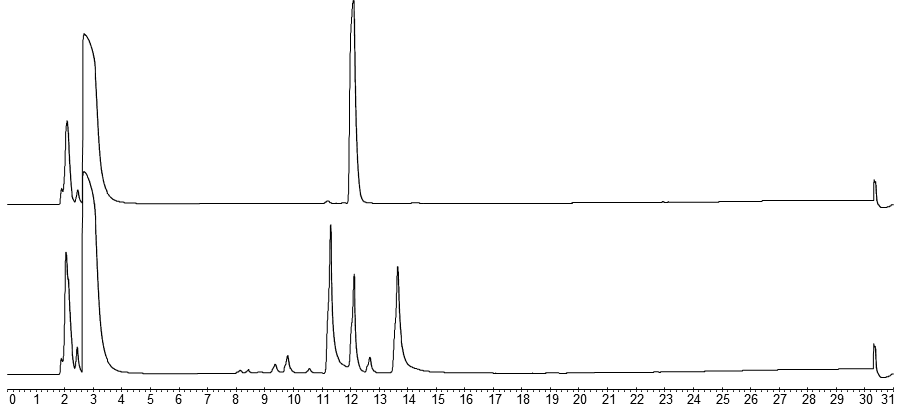


**30b**

**28b**

**29b**

**Supplementary Figure 18.** HPLC chromatograms of negative control (top) and AsqJ-assay (bottom) with (*S*)-3-benzyl-7-fluoro-4-methyl-3,4-dihydro-1*H*-benzo[1,4]diazepine-2,5-dione (**10b**) as substrate.

(*S*)-3-benzyl-8-fluoro-4-methyl-3,4-dihydro-1*H*-benzo[1,4]diazepine-2,5-dione (**10c**)

**10c**


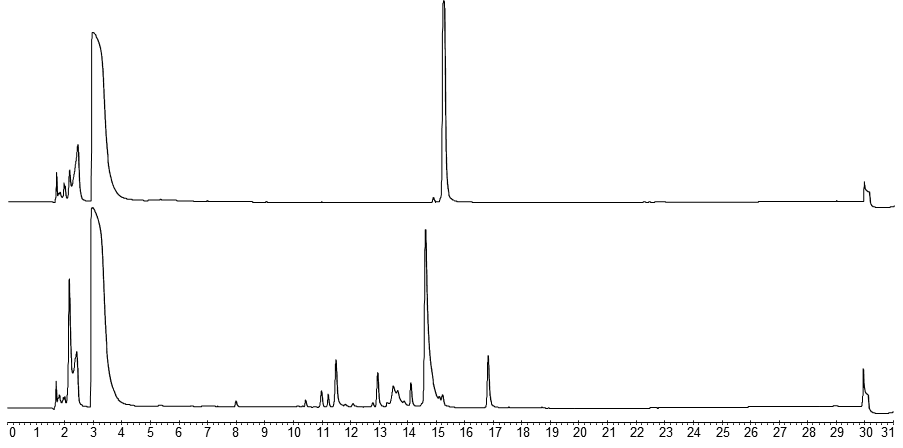


[min]

**30c**

**28c**

*

*

*

**Supplementary Figure 19.** HPLC chromatograms of negative control (top) and AsqJ-assay (bottom) with (*S*)-3-benzyl-8-fluoro-4-methyl-3,4-dihydro-1*H*-benzo[1,4]diazepine-2,5-dione (**10c**) as substrate. *: Unidentified side-products.

(*S*)-3-benzyl-9-fluoro-4-methyl-3,4-dihydro-1*H*-benzo[1,4]diazepine-2,5-dione (**10d**)

**10d**

[min]


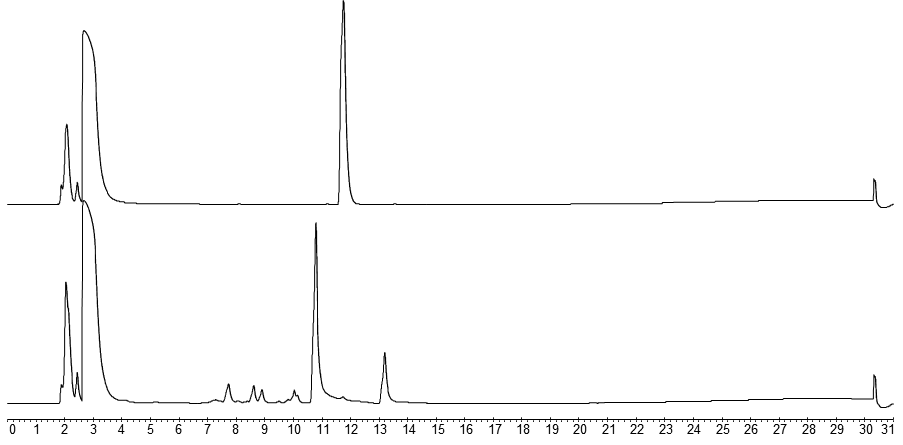


**30d**

**28d**

**Supplementary Figure 20.** HPLC chromatograms of negative control (top) and AsqJ-assay (bottom) with (*S*)-3-benzyl-9-fluoro-4-methyl-3,4-dihydro-1*H*-benzo[1,4]diazepine-2,5-dione (**10d**) as substrate.


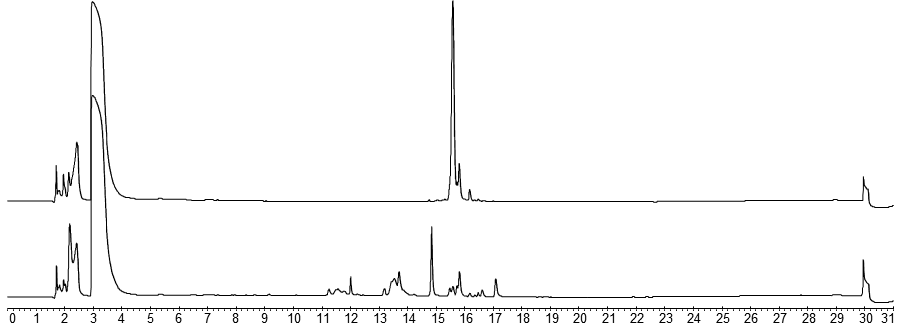
(*S*)-6-chloro-3-benzyl-4-methyl-3,4-dihydro-1*H*-benzo[1,4]diazepine-2,5-dione (**10e**)

**10e**

**30e**

**28e**

[min]

**Supplementary Figure 21.** HPLC chromatograms of negative control (top) and AsqJ-assay (bottom) with (*S*)-6-chloro-3-benzyl-4-methyl-3,4-dihydro-1*H*-benzo[1,4]diazepine-2,5-dione (**10e**) as substrate. Minor peaks in negative control result from inseparable impurities of substrate synthesis.

(*S*)-7-chloro-3-benzyl-4-methyl-3,4-dihydro-1*H*-benzo[1,4]diazepine-2,5-dione (**10f**)

**10f**

[min]


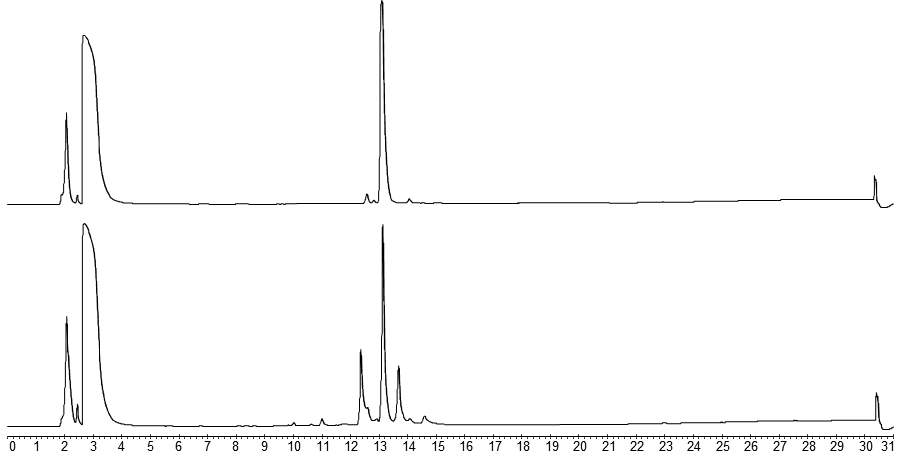


**29f**

**30f**

**28f**

**Supplementary Figure 22.** HPLC chromatograms of negative control (top) and AsqJ-assay (bottom) with (*S*)-7-chloro-3-benzyl-4-methyl-3,4-dihydro-1*H*-benzo[1,4]diazepine-2,5-dione (**10f**) as substrate.

(*S*)-8-chloro-3-benzyl-4-methyl-3,4-dihydro-1*H*-benzo[1,4]diazepine-2,5-dione (**10g**)

**10g**

[min]


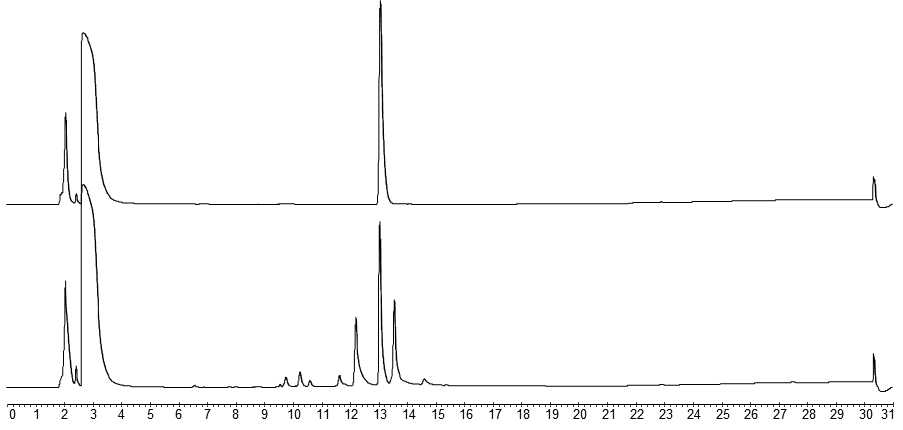


**30g**

**29g**

**28g**

**Supplementary Figure 23.** HPLC chromatograms of negative control (top) and AsqJ-assay (bottom) with (*S*)-8-chloro-3-benzyl-4-methyl-3,4-dihydro-1*H*-benzo[1,4]diazepine-2,5-dione (**10g**) as substrate.

(*S*)-3-benzyl-4,6-dimethyl-3,4-dihydro-1*H*-benzo[1,4]diazepine-2,5-dione (**10i**)

**10i**

[min]


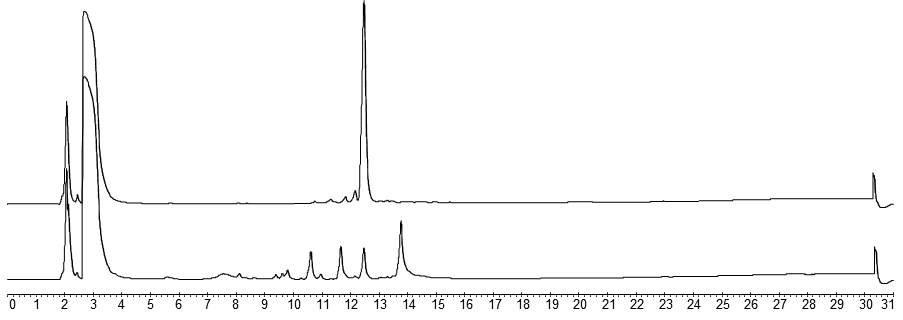


**32i**

**28i**

**30i**

**Supplementary Figure 24.** HPLC chromatograms of negative control (top) and AsqJ-assay (bottom) with (*S*)-3-benzyl-4,6-dimethyl-3,4-dihydro-1*H*-benzo[1,4]diazepine-2,5-dione (**10i**) as substrate. Minor peaks in negative control result from inseparable impurities of substrate synthesis.

(*S*)-3-benzyl-4,7-dimethyl-3,4-dihydro-1*H*-benzo[1,4]diazepine-2,5-dione (**10j**)

**10j**

[min]


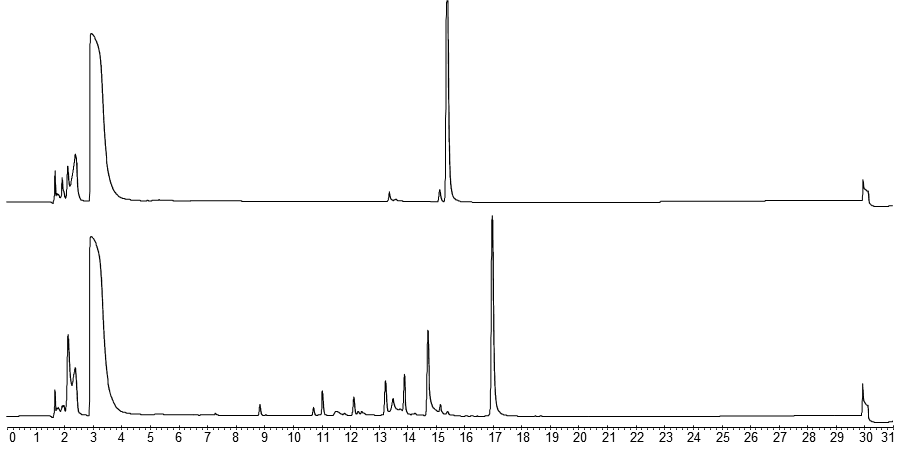


**28j**

**30j**

**32j**

*

**Supplementary Figure 25.** HPLC chromatograms of negative control (top) and AsqJ-assay (bottom) with (*S*)-3-benzyl-4,7-dimethyl-3,4-dihydro-1*H*-benzo[1,4]diazepine-2,5-dione (**10j**) as substrate. *: Unidentified side-product.

(*S*)-3-benzyl-4,9-dimethyl-3,4-dihydro-1*H*-benzo[1,4]diazepine-2,5-dione (**10k**)

**10k**


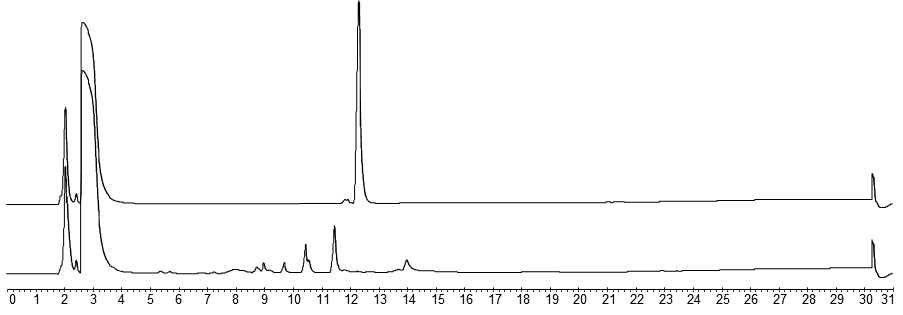


**32k**

**28k**

**30k**

[min]

**Supplementary Figure 26.** HPLC chromatograms of negative control (top) and AsqJ-assay (bottom) with (*S*)-3-benzyl-4,9-dimethyl-3,4-dihydro-1*H*-benzo[1,4]diazepine-2,5-dione (**10k**) as substrate.

(*S*)-3-benzyl-4-methyl-3,4-dihydro-1*H*-naphtho[2,3-*e*][1,4]diazepine-2,5-dione (**10l**)

This assay was performed with 8% (v/v) DMF.

**10l**

[min]


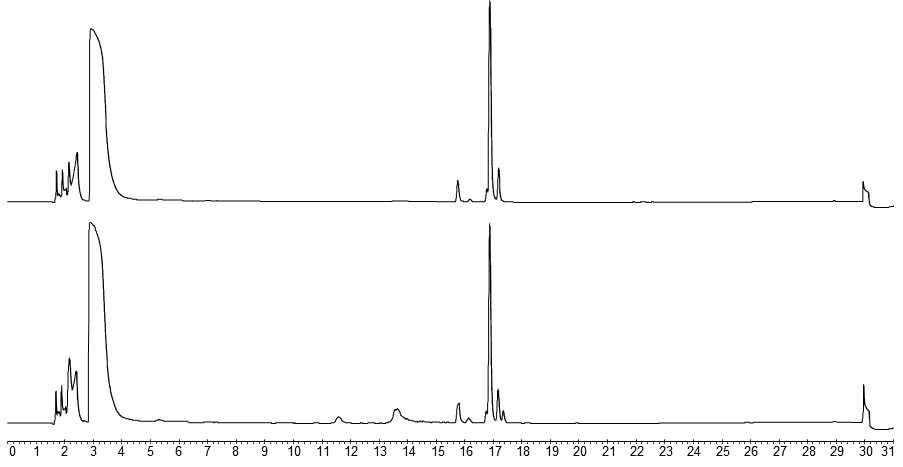


**Supplementary Figure 27.** HPLC chromatograms of negative control (top) and AsqJ-assay (bottom) with (*S*)-3-benzyl-4-methyl-3,4-dihydro-1*H*-naphtho[2,3-*e*][1,4]diazepine-2,5-dione (**10l**) as substrate. Minor peaks in negative control result from inseparable impurities of substrate synthesis.

(*S*)-3-benzyl-4-ethyl-3,4-dihydro-1*H*-benzo[1,4]diazepine-2,5-dione (**10m**)

**10m**

[min]


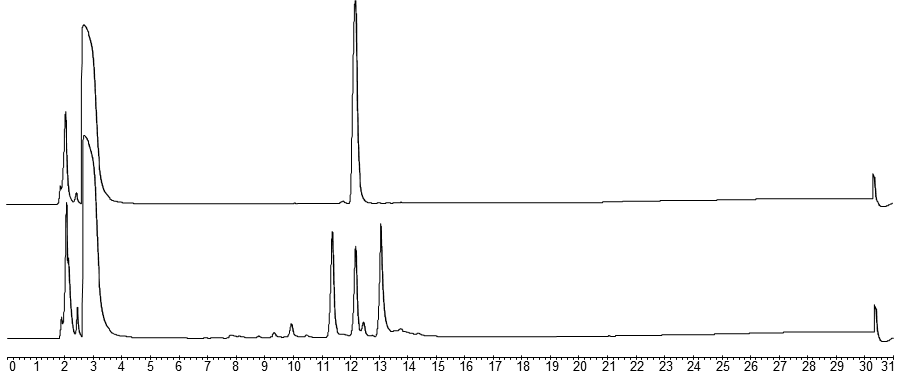


**29m**

**28**

**30m**

**Supplementary Figure 28.** HPLC chromatograms of negative control (top) and AsqJ-assay (bottom) with (*S*)-3-benzyl-4-ethyl-3,4-dihydro-1*H*-benzo[1,4]diazepine-2,5-dione (**10m**) as substrate.

(*S*)-3-(cyclohexylmethyl)-4-ethyl-3,4-dihydro-1*H*-benzo[1,4]diazepine-2,5-dione (**33**)

This assay was performed with 8 vol-% DMF.

**33**


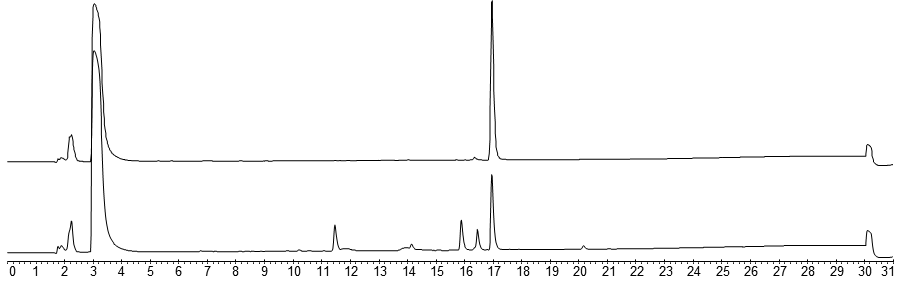


**44**

**43**

**45**

[min]

**Supplementary Figure 29.** HPLC chromatograms of negative control (top) and AsqJ-assay (bottom) with (*S*)-3-(cyclohexylmethyl)-4-ethyl-3,4-dihydro-1*H*-benzo[1,4]diazepine-2,5-dione (**33**) as substrate.

(*S*)-4-ethyl-3-(4-methoxybenzyl)-3,4-dihydro-1*H*-benzo[1,4]diazepine-2,5-dione (**34**)

[min]


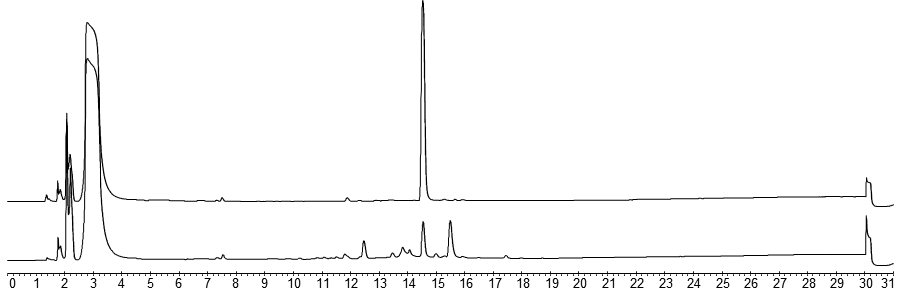


**34**

**SI-28**

**4**

*

**Supplementary Figure 30.** HPLC chromatograms of negative control (top) and AsqJ-assay (bottom) with (*S*)-4-ethyl-3-(4-methoxybenzyl)-3,4-dihydro-1*H*-benzo[1,4]diazepine-2,5-dione (**34**) as substrate. *: Unidentified side-product.

(*S*)-3-benzyl-4-ethyl-1-hydroxy-3,4-dihydro-1*H*-benzo[1,4]diazepine-2,5-dione (**40**)

**40**

[min]


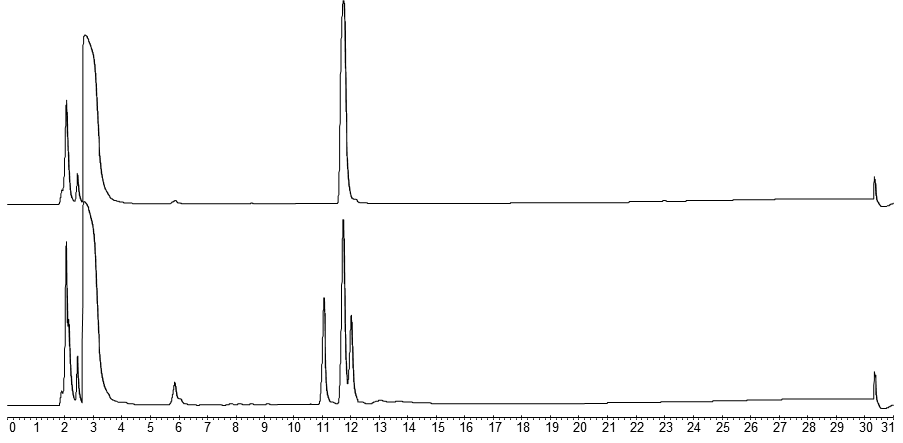


**46**

**47**

**Supplementary Figure 31.** HPLC chromatograms of negative control (top) and AsqJ-assay (bottom) with (*S*)-3-benzyl-4-ethyl-1-hydroxy-3,4-dihydro-1*H*-benzo[1,4]diazepine-2,5-dione (**39**) as substrate.

(*S*)-1,2,3,11a-tetrahydro-5*H*-benzopyrrolo[1,2-*a*][1,4]diazepine-5,11(10*H*)-dione (**54**)

**54**

[min]


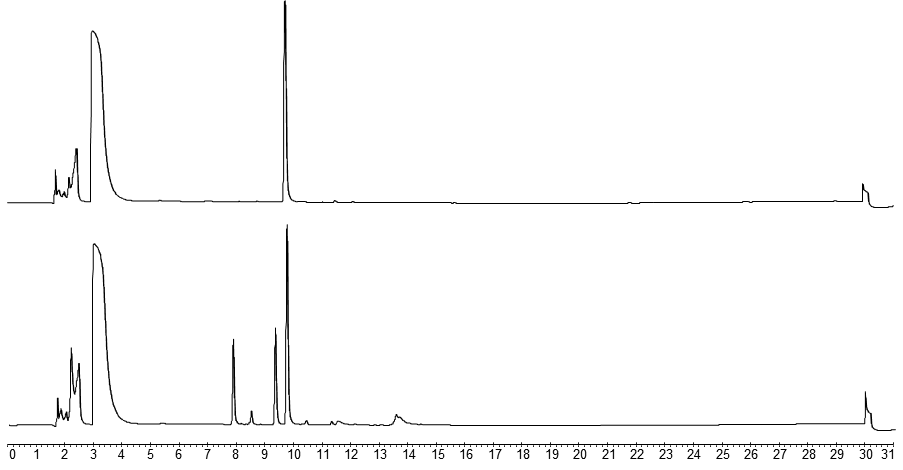


**64**

**74**

*

**Supplementary Figure 32.** HPLC chromatograms of negative control (top) and AsqJ-assay (bottom) with (*S*)-1,2,3,11a-tetrahydro-5*H*-benzopyrrolo[1,2-*a*][1,4]diazepine-5,11(10*H*)-dione (**54**) as substrate. *: Unidentified side-product.

(*S*)-7,8,9,10-tetrahydrobenzopyrido[1,2-*a*][1,4]diazepine-6,12(5*H*,6a*H*)-dione (**55**)

**55**


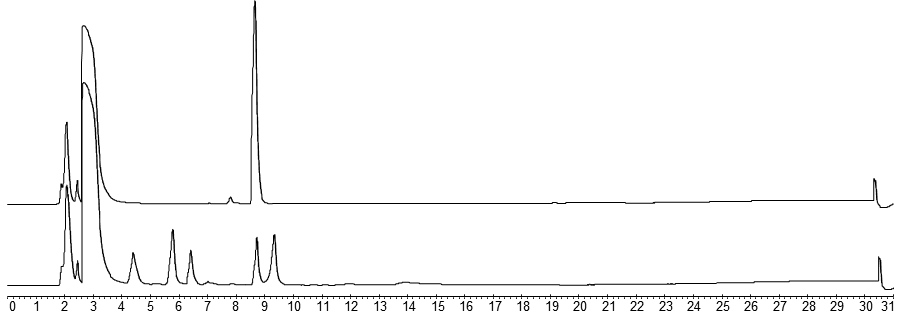


**73**

**72**

**75**

[min]

**Supplementary Figure 33.** HPLC chromatograms of negative control (top) and AsqJ-assay (bottom) with (*S*)-1,2,3,11a-tetrahydro-5*H*-benzopyrrolo[1,2-*a*][1,4]diazepine-5,11(10*H*)-dione (**55**) as substrate.

(2*R*,11a*S*)-2-hydroxy-1,2,3,11a-tetrahydro-5*H*-benzopyrrolo[1,2-*a*][1,4]diazepine-5,11(10*H*)-dione (**69**)

**69**

[min]


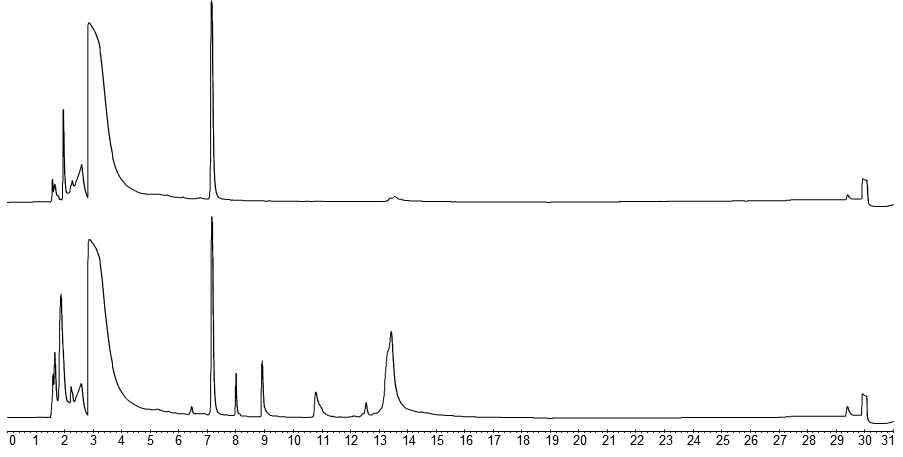


AsqJ

**66**

**70**

*

**Supplementary Figure 34.** HPLC chromatograms of negative control (top) and AsqJ-assay (bottom) with (2*R*,11a*S*)-2-hydroxy-1,2,3,11a-tetrahydro-5*H*-benzopyrrolo[1,2-*a*][1,4]diazepine-5,11(10*H*)-dione (**69**) as substrate. *: Unidentified side-product.

(*S*)-3-isobutyl-8-nitro-4-methyl-3,4-dihydro-1*H*-benzo[1,4]diazepine-2,5-dione (**9h**)

[min]


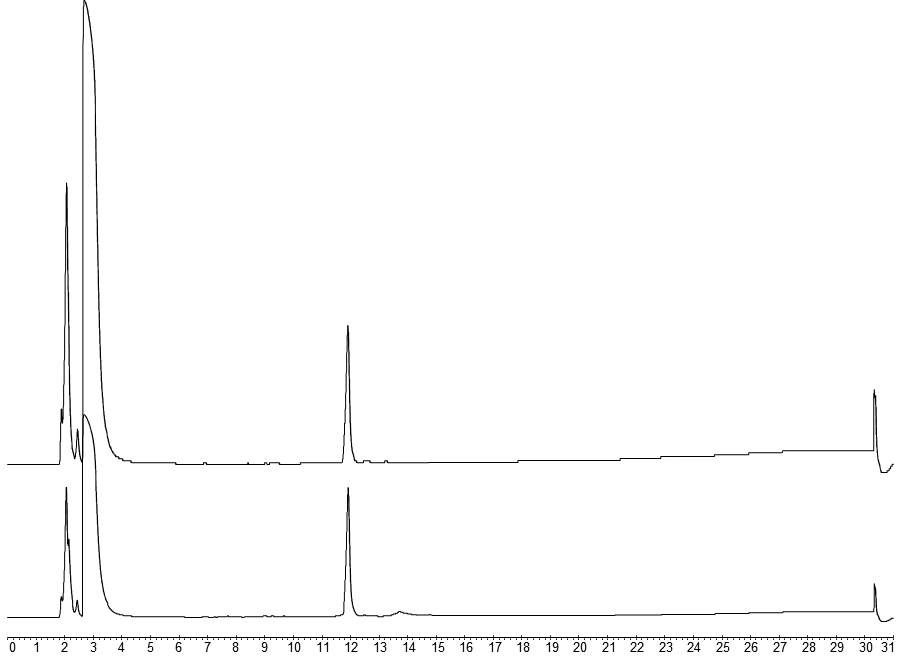


**9h**

**Supplementary Figure 35.** HPLC chromatograms of negative control (top) and AsqJ-assay (bottom) with (*S*)-3-isobutyl-8-nitro-4-methyl-3,4-dihydro-1*H*-benzo[1,4]diazepine-2,5-dione (**9h**) as substrate.

(*S*)-3-benzyl-8-nitro-4-methyl-3,4-dihydro-1*H*-benzo[1,4]diazepine-2,5-dione (**10h**)

**10h**

[min]


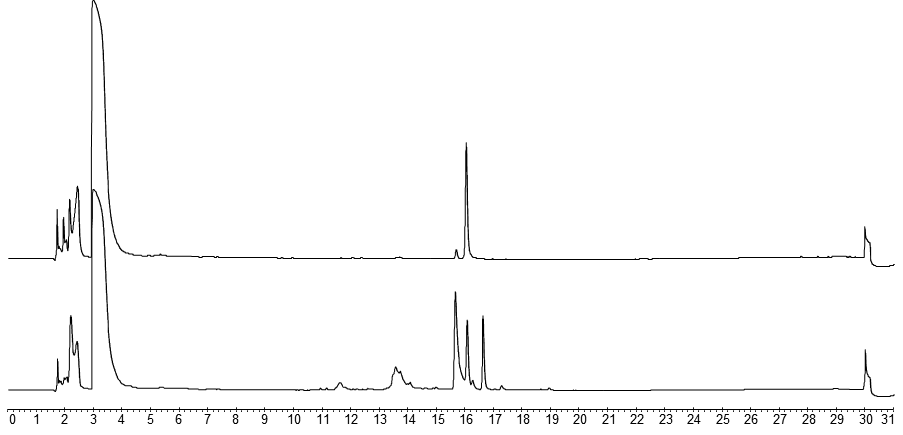


**29h**

**30h**

**Supplementary Figure 36.** HPLC chromatograms of negative control (top) and AsqJ-assay (bottom) with (*S*)-3-benzyl-8-nitro-4-methyl-3,4-dihydro-1*H*-benzo[1,4]diazepine-2,5-dione (**10h**) as substrate.

1,4-dimethyl-3,4-dihydro-1*H*-benzo[1,4]diazepine-2,5-dione (**49n**)

**49n**

[min]


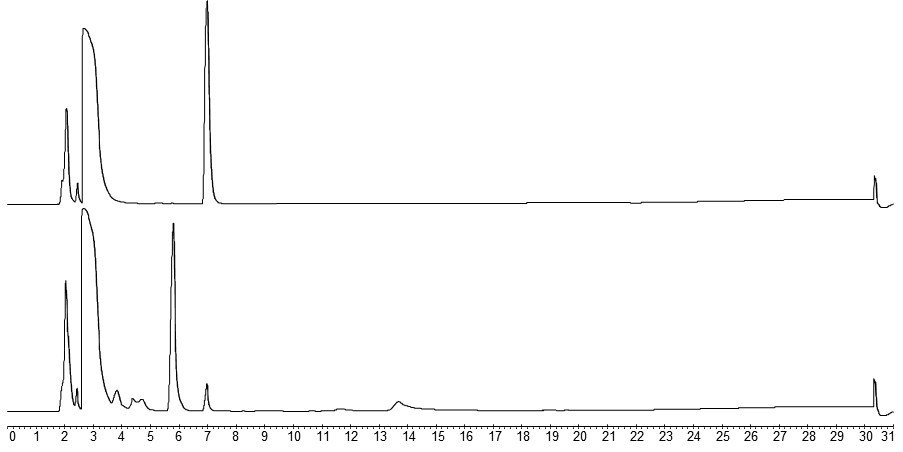


**50**

**Supplementary Figure 37.** HPLC chromatograms of negative control (top) and AsqJ-assay (bottom) with 1,4-dimethyl-3,4-dihydro-1*H*-benzo[1,4]diazepine-2,5-dione (**49n**) as substrate.


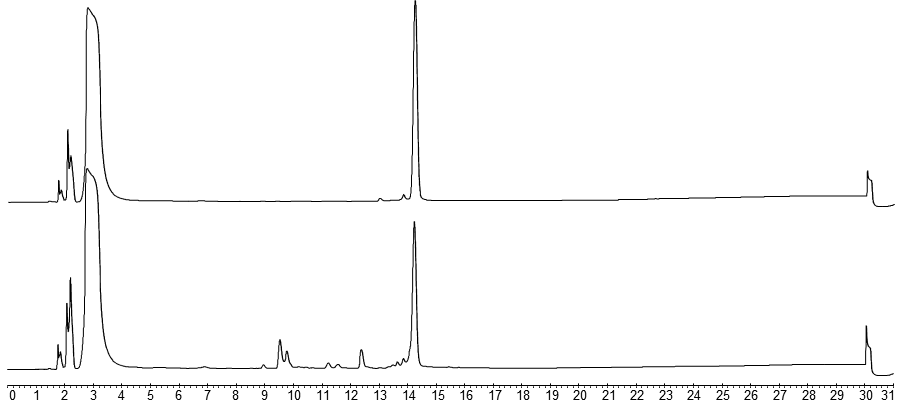
(*S*)-3-isobutyl-1,4-dimethyl-3,4-dihydro-1*H*-benzo[1,4]diazepine-2,5-dione (**9n**)

**9n**

*

*

[min]

**Supplementary Figure 38.** HPLC chromatograms of negative control (top) and AsqJ-assay (bottom) with (*S*)-3-isobutyl-1,4-dimethyl-3,4-dihydro-1*H*-benzo[1,4]diazepine-2,5-dione (**9n**) as substrate. *: Unidentified products.

(*S*)-3-benzyl-1,4-dimethyl-3,4-dihydro-1*H*-benzo[1,4]diazepine-2,5-dione (**10n**)

**10n**

[min]


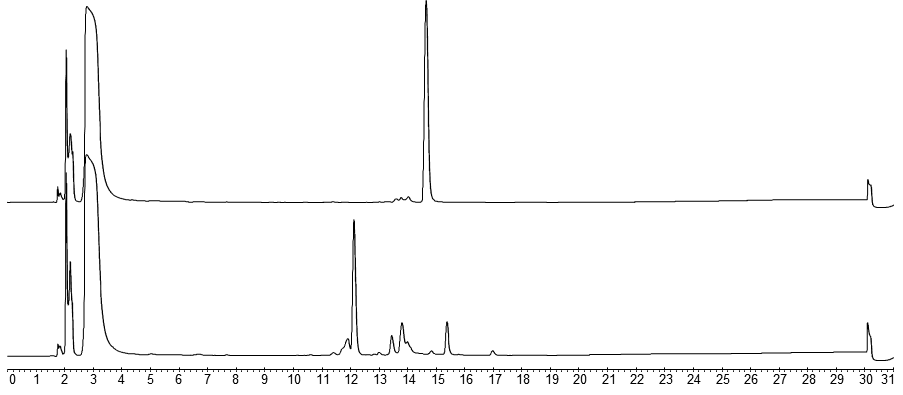


**

**28n**

**30n**

*

**Supplementary Figure 39.** HPLC chromatograms of negative control (top) and AsqJ-assay (bottom) with (*S*)-3-benzyl-1,4-dimethyl-3,4-dihydro-1*H*-benzo[1,4]diazepine-2,5-dione (**10n**) as substrate. *: Unidentified side-product. **: Isolation and identification attempted, but not successful (no pure product obtainable).

(*S*)-3-isobutylbenzo[1,4]oxazepine-2,5(1*H*,3*H*)-dione (**79a**)

**79a**

[min]


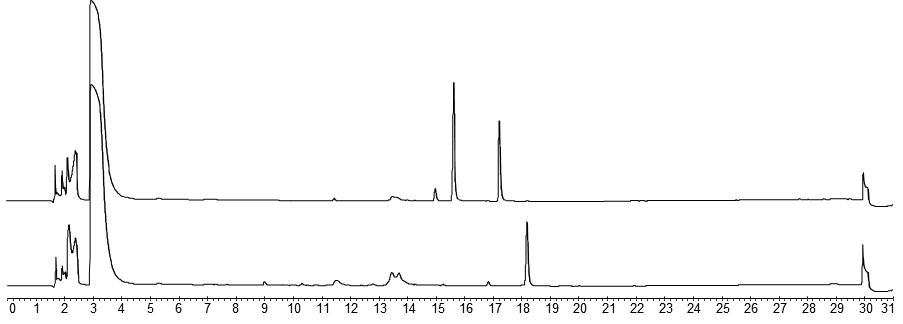


**Supplementary Figure 40.** HPLC chromatograms of negative control (top) and AsqJ-assay (bottom) with (*S*)-3-isobutylbenzo[1,4]oxazepine-2,5(1*H*,3*H*)-dione (**79a**) as substrate.

(*S*)-3-benzylbenzo[1,4]oxazepine-2,5(1*H*,3*H*)-dione (**79b**)

**79b**

[min]


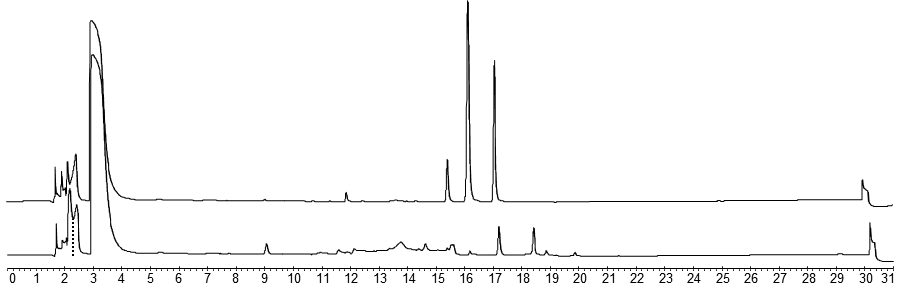


**Supplementary Figure 41.** HPLC chromatograms of negative control (top) and AsqJ-assay (bottom) with (*S*)-3-benzylbenzo[1,4]oxazepine-2,5(1*H*,3*H*)-dione (**79b**) as substrate.

(*S*)-3-isobutyl-4-methyl-3,4-dihydrobenzo[1,4]oxazepine-2,5-dione (**85a**)

**85a**

[min]


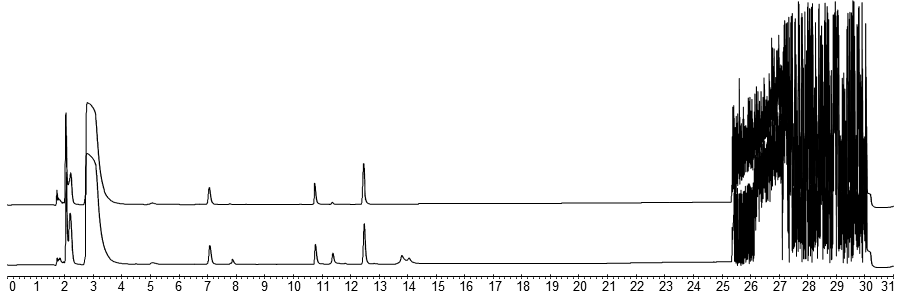


**Supplementary Figure 42.** HPLC chromatograms of negative control (top) and AsqJ-assay (bottom) with crude (*S*)-3-isobutyl-4-methyl-3,4-dihydrobenzo[1,4]oxazepine-2,5-dione (**85a**) as substrate. Other peaks represent impurities from the crude esterification mixture.

(*S*)-3-benzyl-4-methyl-3,4-dihydrobenzo[1,4]oxazepine-2,5-dione (**85b**)

**85b**

[min]


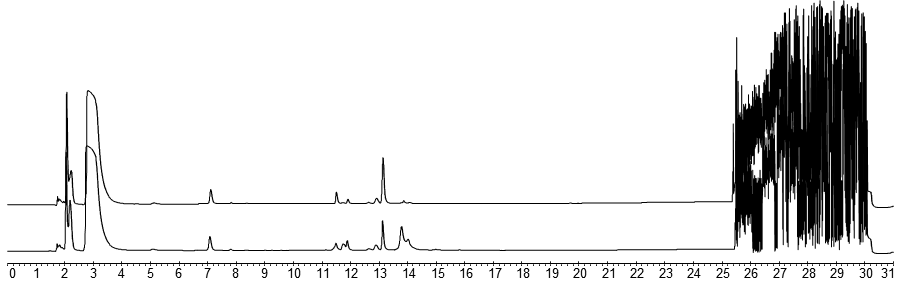


**Supplementary Figure 43.** HPLC chromatograms of negative control (top) and AsqJ-assay (bottom) with crude (*S*)-3-benzyl-4-methyl-3,4-dihydrobenzo[1,4]oxazepine-2,5-dione (**85b**) as substrate. Other peaks represent impurities from the crude esterification mixture.

**Supplementary Table 2.** Overview over products of enzymatic transformation by AsqJ.

| Substrate | Product(s) | Substrate | Product(s) |
| --- | --- | --- | --- |
|  |  |  |  |
|  |  |  |  |
|  |  |  |  |
|  |  |  | – |
|  |  |  |  |
|  |  |  |  |
|  | – |  |  |
|  | – |  |  |
|  |  |  |  |
|  |  |  |  |
|  |  |  |  |
|  | – |  | – |
|  |  |  |  |
|  | + unidentified side-products |  |  |
|  |  |  | Unidentified trace products |
|  |  |  | + unstable side-product |
|  | + unidentified side-products |  | Substrate unstable under assay conditions |
|  |  |  | Substrate unstable under assay conditions |
|  |  |  | – |
|  |  |  | – |
|  |  |  |  |

## **3.3 Upscaled Assays for Product Structure Elucidation**

(Semi-)preparative enzymatic assays were carried out as described for the analytical ones, but with an altered total volume and slight modifications. These are given for each substrate. Reactions were stopped by acidification with TFA (see above), and precipitants removed by centrifugation. Preparative HPLC of the supernatant (detection at 220 nm) yielded the products.

**(*S*)-8-fluoro-3-isobutyl-4-methyl-3,4-dihydro-1*H*-benzo[1,4]diazepine-2,5-dione (9c)**

1800 µL in total, 7 h at 28 °C; TRIS HCL buffer (50 mm, pH 7.4);

containing 1.5 mm of the substrate (dissolved in DMF: 100 mm), 50 µm (3.3 mol-%) of purified AsqJ, 2.5 mm α-ketoglutarate, 4 mm ascorbic acid, 100 µm iron sulfate and 5% (v/v) DMF

Fraction 1: 7-fluoro-3-methylquinazoline-2,4(1*H*,3*H*)-dione (**22c**)

**^1^H-NMR** (600 MHz, DMSO-d_6_)**:** δ [ppm] = 11.55 (bs, 1 H), 8.00 (dd, *J* = 8.8, 6.1 Hz, 1 H), 7.05 (td, *J* = 8.8, 2.4 Hz, 1 H), 6.91 (dd, *J* = 9.8, 2.5 Hz, 1 H), 3.24 (s, 3 H).

**^19^F-NMR** (282 MHz, DMSO-d_6_)**:** δ [ppm] = –105.02 (s).

NMR data matched those reported in literature.^8^

**HR-MS** (ESI–)**:** *m/z* calcd. for C_9_H_7_FN_2_O_2_ [M–H]^–^: 193.0419, found: 193.0424.

Fraction 2: 7-fluoro-2-isobutyl-3-methylquinazolin-4(3*H*)-one (**21c**)

**^1^H-NMR** (600 MHz, DMSO-d_6_)**:** δ [ppm] = 8.16 (dd, *J* = 8.8, 6.3 Hz, 1 H), 7.39–7.32 (m, 2 H), 3.53 (s, 3 H), 2.74 (d, *J* = 6.9 Hz, 2 H), 2.28 (h, *J* = 6.8 Hz, 1 H), 1.00 (d, *J* = 6.6 Hz, 6 H).

**^19^F-NMR** (282 MHz, DMSO-d_6_)**:** δ [ppm] = –106.46 (s).

**HR-MS** (ESI+)**:** *m/z* calcd. for C_13_H_15_FN_2_O [M+H]^+^: 235.1241, found: 235.1238.

**(*S*)-3,4-dimethyl-3,4-dihydro-1*H*-benzo[1,4]diazepine-2,5-dione-2-^13^*C* (23)^2^**

1800 µL in total (4 portions), 5 h at 28 °C; TRIS HCL buffer (50 mm, pH 7.4);

containing 1 mm of the substrate (dissolved in DMF: 100 mm), 50 µm (5 mol-%) of purified AsqJ, 2.5 mm α-ketoglutarate, 4 mm ascorbic acid, 100 µm iron sulfate and 5% (v/v) DMF

Fraction 1: 2,3-dimethylquinazolin-4(3*H*)-one (**24**)

**HR-MS** (ESI+)**:** *m/z* calcd. for C_10_H_10_N_2_O [M+H]^+^: 175.0866, found: 175.0873.

Fraction 2: 3-methylquinazoline-2,4(1*H*,3*H*)-dione-2-^13^*C* (**25**)

**^1^H-NMR** (600 MHz, DMSO-d_6_)**:** δ [ppm] = 11.45 (bs, 1 H), 7.93 (dd, *J* = 7.9, 1.5 Hz, 1 H), 7.67–7.63 (m, 1 H), 7.20 (ddd, *J* = 8.1, 7.2, 1.0 Hz, 1 H), 7.18 (bd, *J* = 8.2 Hz, 1 H), 3.25 (d, ^3^*J* (^1^H–^13^C) = 2.8 Hz, 3 H).

**^13^C-NMR** (151 MHz, DMSO-d_6_): isolated yield too low, but labelled carbon clearly visible: δ [ppm] = 150.4.

Spectroscopic data in accordance with literature data reported for unlabelled compound **25**.^9^

**HR-MS** (ESI+)**:** *m/z* calcd. for C_8_^13^CH_8_N_2_O_2_ [M+H]^+^: 178.0692, found: 178.0689.

**1,4-dimethyl-3,4-dihydro-1H-benzo[1,4]diazepine-2,5-dione (49n)**

1800 µL in total (3 portions), 7 h at 28 °C; TRIS HCL buffer (50 mm, pH 7.4);

containing 1.5 mm of the substrate (dissolved in DMF: 100 mm), 50 µm (3.3 mol-%) of purified AsqJ, 2.5 mm α-ketoglutarate, 4 mm ascorbic acid, 100 µm iron sulfate and 5% (v/v) DMF

3-hydroxy-1,4-dimethyl-3,4-dihydro-1*H*-benzo[1,4]diazepine-2,5-dione (**50**):

**^1^H-NMR** (600 MHz, ACN-d_3_)**:** δ [ppm] = 7.74 (dd, *J* = 7.8, 1.7 Hz, 1 H), 7.62–7.57 (m, 1 H), 7.38–7.32 (m, 2 H), 5.18 (d, *J* = 8.2 Hz, 1 H), 4.88 (d, *J* = 8.3 Hz, 1 H), 3.39 (s, 3 H), 2.94 (s, 3 H).

**HR-MS** (ESI+)**:** *m/z* calcd. for C_11_H_12_N_2_O_3_ [M+Na]^+^: 243.0740, found: 243.0738.

Investigation of optical purity of **50**:

600 µL in total (3 portions), 3.3 mol-% AsqJ, 9 h at 28 °C; TRIS HCL buffer (50 mm, pH 7.4);

containing 1.5 mm of the substrate (dissolved in DMF: 100 mm), 50 µm (3.3 mol-%) of purified AsqJ, 2.5 mm α-ketoglutarate, 4 mm ascorbic acid, 100 µm iron sulfate and 5% (v/v) DMF


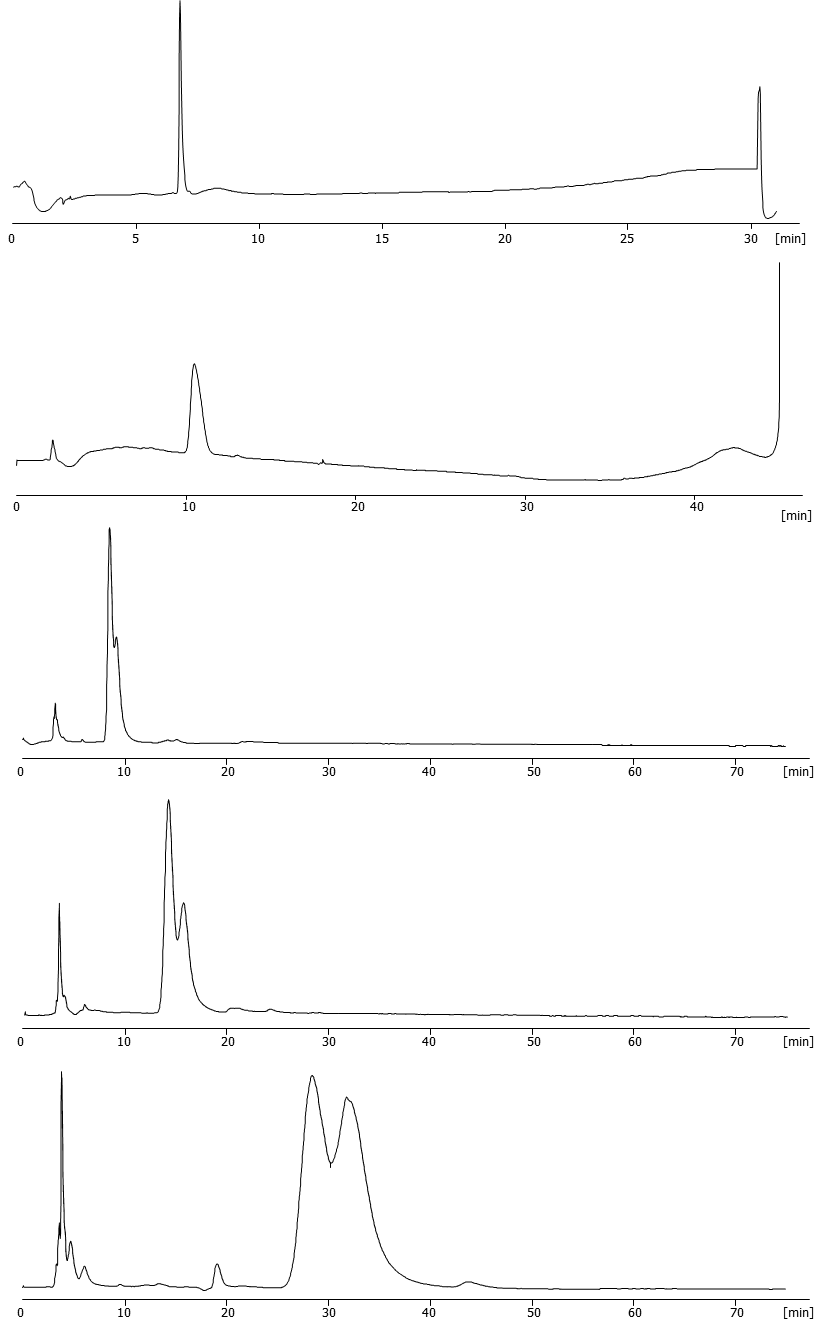


**b)**

**a)**

**d)**

**e)**

**c)**

**Supplementary Figure 44.** HPLC chromatograms of purified **50**. a) On C18 column with method described in chapter 1.1; b) to e): on a EC250/4.6 Nucleocell Delta-RP chiral column with different methods; flow 0.7 mL/min. b) Gradient as described in chapter 1.1, elongated to 45 minutes. c) 16% ACN, 84% H_2_O, isocratic over 75 minutes. d) 11% ACN, 89% H_2_O, isocratic over 75 minutes. e) 6% ACN, 94% H_2_O, isocratic over 75 minutes.

H_2_^18^O labelling experiment with **49n**

350 µL, 4 h at 28 °C; in H_2_^18^O;

containing 1 mm of the substrate (dissolved in DMF: 100 mm), 50 µm (5 mol-%) of purified AsqJ (2 × pre-concentrated with H_2_^18^O and then diluted with H_2_^18^O 1:1), 2.5 mm α-ketoglutarate, 4 mm ascorbic acid, 100 µm iron sulfate and 5% (v/v) DMF; approx. 80% (v/v) H_2_^18^O in total

Slow addition of enzyme to the assay mixture was necessary to avoid instant precipitation.

**Supplementary Figure 45.** Left: HPLC chromatogram of negative control (top) and AsqJ-assay (bottom) with 1,4-dimethyl-3,4-dihydro-1*H*-benzo[1,4]diazepine-2,5-dione (**49n**) as substrate and H_2_^18^O as main solvent. Right: Mass spectrum of product signal (retention time 8.2 minutes).

**50 /**

**50-^18^O**

Intens.

221.0911

223.0953

243.0728

245.0771

0

20

40

60

80

100

[%]

220

225

230

235

240

245

m/z

t [min]


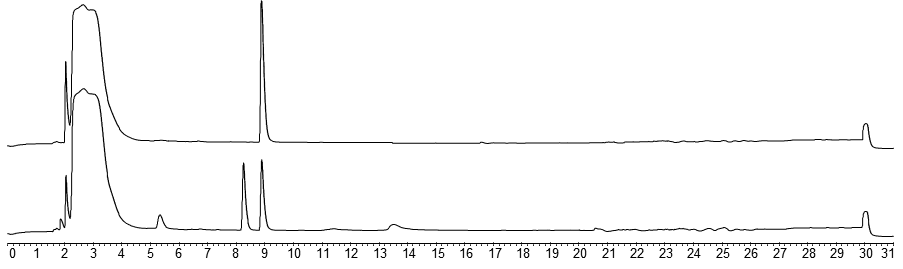


**49n**

56%

**(*S*)-1,2,3,11a-tetrahydro-5*H*-benzopyrrolo[1,2-*a*][1,4]diazepine-5,11(10*H*)-dione (54)**

2000 µL in total (4 portions), 17 h at 28 °C; TRIS HCL buffer (50 mm, pH 7.4);

containing 2 mm of the substrate (dissolved in DMF: 100 mm), 100 µm (5 mol-%) of purified AsqJ, 2.5 mm α-ketoglutarate, 4 mm ascorbic acid, 100 µm iron sulfate and 5% (v/v) DMF

Fraction 1: Presumably 9-oxo-2,3,4,9-tetrahydropyrrolo[2,1-*b*]quinazoline-3a(1*H*)-carboxylic acid (**74**)

**^1^H-NMR** (600 MHz, DMSO-d_6_)**:** δ [ppm] = 10.46 (bs, 1 H), 7.75 (dd, *J* = 7.9, 1.6 Hz, 1 H), 7.44 (ddd, *J* = 8.0, 7.2, 1.7 Hz, 1 H), 7.13 (ddd, *J* = 8.3, 7.2, 1.2 Hz, 1 H), 7.08 (dd, *J* = 8.1, 1.1 Hz, 1 H), 6.59 (s, 1 H), 3.70–3.65 (m, 1 H), 3.64–3.60 (m, 1 H), 2.00–1.87 (m, 3 H), 1.70–1.62 (m, 1 H).

**HR-MS** (ESI–)**:** *m/z* calcd. for C_12_H_12_N_2_O_3_ [M–H]^–^: 231.0775, found: 231.0772.

Fraction 2: 2,3-dihydropyrrolo[2,1-*b*]quinazolin-9(1*H*)-one / deoxyvasicinone (**64**)

**^1^H-NMR** (600 MHz, DMSO-d_6_)**:** δ [ppm] = 8.12 (dd, *J* = 7.9, 1.6 Hz, 1 H), 7.78 (ddd, *J* = 8.5, 7.1, 1.6 Hz, 1 H), 7.61 (dd, *J* = 8.5, 1.3 Hz, 1 H), 7.47 (ddd, *J* = 8.1, 7.1, 1.2 Hz, 1 H), 4.09–4.04 (m, 2 H), 3.08 (t, *J* = 7.9 Hz, 2 H), 2.17 (dt, *J* = 15.1, 7.8 Hz, 2 ).

**HR-MS** (ESI+)**:** *m/z* calcd. for C_11_H_10_N_2_O [M+H]^+^: 187.0866, found: 187.0866.

Analytical data correspond to those reported in literature.^10^

**(2*R*,11a*S*)-2-hydroxy-1,2,3,11a-tetrahydro-5*H*-benzopyrrolo[1,2-*a*][1,4]diazepine-5,11(10*H*)-dione (69)**

2500 µL in total (5 portions), 1 d at 28 °C; TRIS HCL buffer (50 mm, pH 7.4);

containing 1.5 mm of the substrate (dissolved in DMF: 100 mm), 50 µm (3.3 mol-%) of purified AsqJ, 2.5 mm α-ketoglutarate, 4 mm ascorbic acid, 100 µm iron sulfate and 3% (v/v) DMF

Fraction 1: (*R*)-2-hydroxy-2,3-dihydropyrrolo[2,1-*b*]quinazolin-9(1*H*)-one (isovasicinone, **66**)

**^1^H-NMR** (600 MHz, DMSO-d_6_)**:** δ [ppm] = 8.13 (dd, *J* = 7.9, 1.6 Hz, 1 H), 7.80 (ddd, *J* = 8.1, 7.1, 1.6 Hz, 1 H), 7.62 (bd, *J* = 8.1 Hz, 1 H), 7.49 (ddd, *J* = 8.1, 7.0, 1.2 Hz, 1 H), 5.48 (bs, 1 H), 4.57–4.53 (m, 1 H), 4.10 (dd, *J* = 12.5, 4.6 Hz, 1 H), 3.99 (dt, *J* = 12.4, 1.3 Hz, 1 H), 3.37 (dd, *J* = 17.2, 5.6 Hz, 1 H)*, 2.87 (dt, *J* = 17.2, 1.4 Hz).

*: Signal overlaps with water peak in DMSO; δ and *J* obtained from ^1^H with water suppression.

**HR-MS** (ESI+)**:** *m/z* calcd. for C_11_H_10_N_2_O_2_ [M+H]^+^: 203.0816, found: 203.0827.

Analytical data correspond to those reported in literature.^11^

Fraction 2: Presumably 5*H*-benzopyrrolo[1,2-*a*][1,4]diazepine-5,11(10*H*)-dione (**70**)

**HR-MS** (ESI+)**:** *m/z* calcd. for C_12_H_9_N_2_O_2_ [M+H]^+^: 213.0659, found: 213.0655.

**(*S*)-3-benzyl-8-fluoro-4-methyl-3,4-dihydro-1*H*-benzo[1,4]diazepine-2,5-dione (10c)**

**(*S*)-3-benzyl-4,7-dimethyl-3,4-dihydro-1*H*-benzo[1,4]diazepine-2,5-dione (10j)**

10 mL in total (1 portion), incubation at 38 °C, shaking at 60 rpm; TRIS HCL buffer (50 mm, pH 7.4);

containing 2 mm of the substrate (dissolved in DMF: 100 mm), 50 µm (2.5 mol-%) of purified AsqJ, 2.5 mm α-ketoglutarate, 4 mm ascorbic acid, 100 µm iron sulfate and 5% (v/v) DMF

For the large-scale enzymatic assays with substrates **10c** and **10j**, conversion was tracked by HPLC after 90/100 minutes. Quenching was conducted by addition of 1) ACN (50% v/v) or 2) TFA (2.5% v/v) and centrifugation (9700× *g*, 15 min). After incubation for 5 hours in total, the mixture was extracted with DCM (3 × 15 mL). Combined organic extracts were washed with brine (20 mL), dried over Na_2_SO_4_, filtered and the volatiles removed *in vacuo*. The residue was dissolved in DCM (4 mL) and 100 µL TFA was added. The mixture was stirred at room temperature overnight, before addition of saturated NH_4_Cl (10 mL) and extraction with DCM (3 × 15 mL). Combined organic phases were washed with brine (10 mL), dried over Na_2_SO_4_, filtered and concentrated under reduced pressure. The residue was purified by preparative HPLC at 220 nm.

[min]


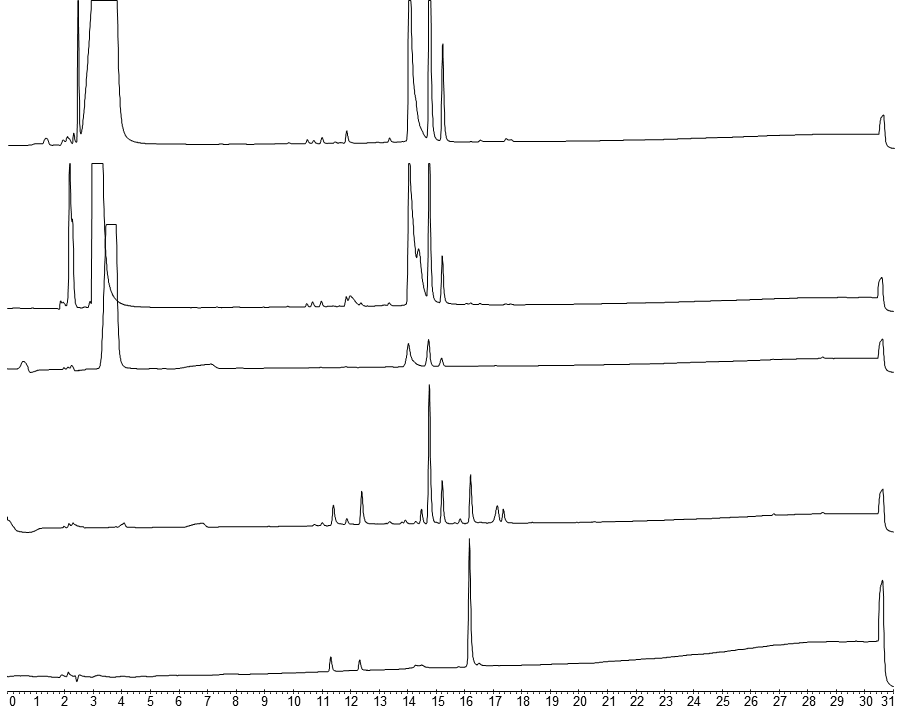


After 90 min,

quenched with ACN

After 90 min,

quenched with TFA

After first

work-up

After TFA-addition

and second work-up

After purification

[min]


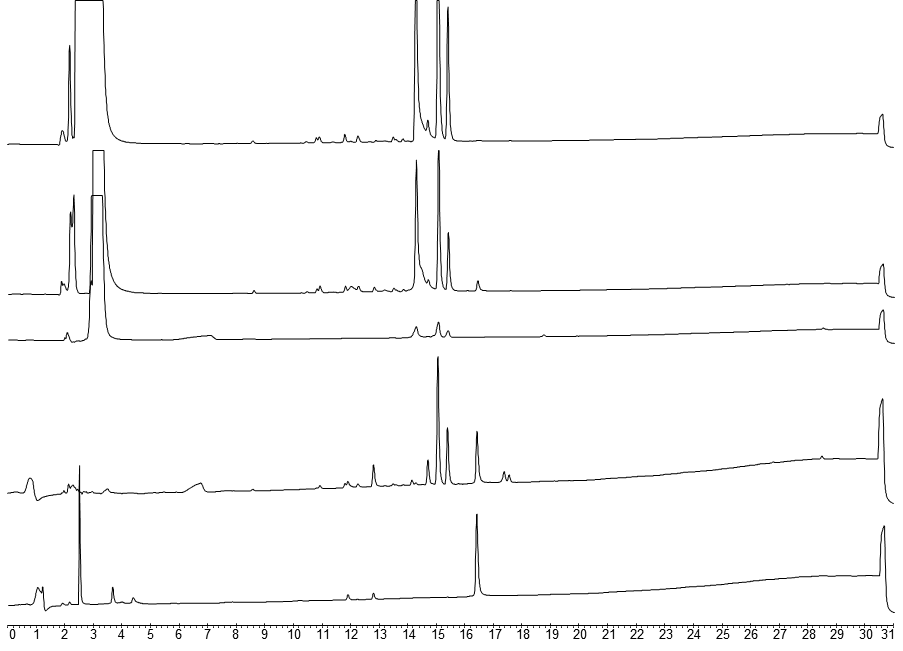


After 100 min,

quenched with ACN

After 100 min,

quenched with TFA

After first

work-up

After TFA-addition

and second work-up

After purification

**Supplementary Figure 46.** HPLC monitoring of preparative AsqJ assays with substrates **10c** (left) and **10j** (right).

7-fluoro-3-hydroxy-4-phenylquinolin-2(1*H*)-one (**28c**)

**HR-MS** (ESI–)**:** *m/z* calcd. for C_15_H_10_FNO_2_ [M–H]^–^: 254.0622, found: 254.0621.

3-hydroxy-6-methyl-4-phenylquinolin-2(1*H*)-one (**28j**)

**HR-MS** (ESI+)**:** *m/z* calcd. for C_16_H_13_NO_2_ [M+Na]^+^: 274.0838, found: 274.0835.

For the preparative isolation of **28j**, the assay was conducted as follows:

10 mL in total (2 portions), incubation at r.t., shaking at 60 rpm; TRIS HCL buffer (50 mm, pH 7.4);

containing 2 mm of the substrate (dissolved in DMF: 100 mm), 50 µm (2.5 mol-%) of purified AsqJ, 2.5 mm α-ketoglutarate, 4 mm ascorbic acid, 100 µm iron sulfate and 5% (v/v) DMF

After incubation for 5 hours, the mixture was extracted with DCM (3 × 10 mL). Combined organic extracts were dried over Na_2_SO_4_, filtered and the volatiles removed *in vacuo*. The residue was dissolved in DCM (4 mL) and 1.3 µL BF_3_•OEt_2_ (1.5 mg, 10.3 µmol, 0.5 eq.) was added at 0 °C. The mixture was stirred at room temperature for 2 hours, before addition of saturated NH_4_Cl (5 mL) and extraction with DCM (3 × 10 mL). Combined organic phases were dried over Na_2_SO_4_, filtered and concentrated under reduced pressure. The residue was purified by preparative HPLC at 220 nm.

Isolated as white solid (0.8 mg, 2.4 µmol, 23% brsm).

**^1^H-NMR** (500 MHz, CDCl_3_)**:** δ [ppm] = 10.43 (bs, 1 H), 7.58–7.53 (m, 2 H), 7.51–7.47 (m, 1 H), 7.46–7.42 (m, 2 H), 7.22 (bs, 2 H), 7.12 (bs, 1 H), 6.86 (bs, 1 H), 2.32 (s, 3 H).

**(*S*)-3-benzyl-9-fluoro-4-methyl-3,4-dihydro-1*H*-benzo[1,4]diazepine-2,5-dione (10d)**

5 mL in total (5 portions), incubation at 28 °C, shaking at 300 rpm;

containing 2 mm of the substrate (dissolved in DMF: 100 mm), 50 µm (2.5 mol-%) of purified AsqJ, 2.5 mm α-ketoglutarate, 4 mm ascorbic acid, 100 µm iron sulfate and 5% (v/v) DMF

After incubation for 7 hours in total, the assays were centrifuged (2 min, 11350 × *g*) and the supernatants combined, before being extracted with DCM (3 × 10 mL). Combined organic extracts were washed with brine (20 mL), dried over Na_2_SO_4_, filtered and the volatiles removed *in vacuo*. The residue was dissolved in DCM (5.5 mL), split into 16 equal portions and the solvent removed by evaporation in a Speedvac concentrator. The residues were dissolved in 400 µL of the corresponding solvent and 10 µL of TFA or 5 µL of BF_3_•OEt_2_ were added. The assays were incubated at room temperature or 50 °C for 75 minutes. Afterwards, the volatiles were removed by evaporation in a Speedvac concentrator and the residues dissolved in MeOH, before being analysed *via* HPLC at 220 nm.

[min]

31

30

29

28

27

26

25

24

23

22

21

20

19

18

17

16

15

14

13

12

11

10

9

8

7

6

5

4

3

2

1

0

After work-up

Acetone,

r.t., TFA

CHCl_3_,

r.t., TFA

MeOH,

r.t., TFA

THF,

r.t., TFA

Acetone,

50 °C, TFA

CHCl_3_,

50 °C, TFA

MeOH,

50 °C, TFA

THF,

50 °C, TFA

Negative control

**Supplementary Figure 47.** Screening of conditions for fragmentation reaction of epoxide **30d** to quinolone **28d**; part 1 (TFA).

31

30

29

28

27

26

25

24

23

22

21

20

19

18

17

16

15

14

13

12

11

10

9

8

7

6

5

4

3

2

1

0

After work-up

Acetone,

r.t., BF_3_OEt_2_

CHCl_3_,

r.t., BF_3_OEt_2_

MeOH,

r.t., BF_3_OEt_2_

THF,

r.t., BF_3_OEt_2_

Acetone,

50 °C, BF_3_OEt_2_

CHCl_3_,

50 °C, BF_3_OEt_2_

MeOH,

50 °C, BF_3_OEt_2_

THF,

50 °C, BF_3_OEt_2_

[min]

Negative control

**Supplementary Figure 48.** Screening of conditions for fragmentation reaction of epoxide **30d** to quinolone **28d**; part 2 (BF_3_•OEt_2_).

8-fluoro-3-hydroxy-4-phenylquinolin-2(1*H*)-one (**28d**)

**HR-MS** (ESI+)**:** *m/z* calcd. for C_15_H_10_FNO_2_ [M+H]^+^: 256.0769, found: 256.0771.

9-fluoro-3-hydroxy-3-(hydroxy(phenyl)methyl)-4-methyl-3,4-dihydro-1*H*-benzo[1,4]diazepine-2,5-dione (**31d**)

**HR-MS** (ESI+)**:** *m/z* calcd. for C_17_H_15_FN_2_O_4_ [M+H]^+^: 331.1089, found: 331.1085.

## **3.4 Quantification of Substrate Consumption**

Calibration curves were created from different dilutions of the corresponding substrates (dissolved in DMF: 100 mm) in buffer (50 mm TRIS HCl, pH 7.4) and DMF (5% v/v), which were acidified with TFA (25 µL TFA/1 mL mixture).

Assays were performed on a 1 mL scale (28 °C, 250 rpm), containing 1 mm of the substrate (dissolved in DMF: 100 mm), 25 µm (2.5 mol-%) of purified AsqJ, 2.5 mm α-ketoglutarate, 4 mm ascorbic acid, 100 µm iron sulfate and 5% (v/v) DMF. The reaction buffer contained 50 mm TRIS HCl at a pH of 7.4. To detect start concentrations, a sample was taken from the pre-mixed master mix solution. At the corresponding time points, 200 µL were separated, quenched by addition of 5 µL TFA, centrifuged at 11350 × *g* for 3 minutes to pelletise precipitated enzyme, and the supernatant used for HPLC analysis.

(*S*)-8-fluoro-3-isobutyl-4-methyl-3,4-dihydro-1*H*-benzo[1,4]diazepine-2,5-dione (**9c**)

**Calibration**

**Supplementary Table 3.** HPLC calibration values for substrate **9c**.

| Peak Area [mAU·s] | n [nmol] |
| --- | --- |
| 830 | 0.625 |
| 1648 | 1.25 |
| 3272 | 2.5 |
| 6423 | 5 |
| 11540 | 10 |
| 15027 | 15 |
| 16597 | 20 |
| 18672 | 25 |

**Supplementary Figure 49.** HPLC calibration curve for substrate **9c**.

**Assay**

**Supplementary Table 4.** HPLC-based analysis of substrate consumption (**9c**) by AsqJ.

| **9c** | Entry 1 | | | Entry 2 | | | Average Conversion [%] |
| --- | --- | --- | --- | --- | --- | --- | --- |
| t [min] | PA [mAU·s] | n [nmol] | Conversion [%] | PA [mAU·s] | n [nmol] | Conversion [%] |  |
| 0 | 18843 | 26.511 | − | 18880 | 26.632 | − | − |
| 15 | 18271 | 24.705 | 6.8 | 18531 | 25.513 | 4.2 | 5.5 |
| 60 | 16781 | 20.496 | 22.7 | 16391 | 19.506 | 26.8 | 24.7 |
| 180 | 8260 | 6.742 | 74.6 | 7233 | 5.839 | 78.1 | 76.3 |
| 360 | 3544 | 2.946 | 88.9 | 3888 | 3.215 | 87.9 | 88.4 |

**Supplementary Figure 50.** Substrate (**9c**) consumption by AsqJ over reaction time.

(*S*)-3-benzyl-8-fluoro-4-methyl-3,4-dihydro-1*H*-benzo[1,4]diazepine-2,5-dione (**10c**)

**Calibration**

**Supplementary Table 5.** HPLC calibration values for substrate **10c**.

| Peak Area [mAU·s] | n [nmol] |
| --- | --- |
| 914 | 0.625 |
| 1806 | 1.25 |
| 3629 | 2.5 |
| 6936 | 5 |
| 12092 | 10 |
| 15627 | 15 |
| 17156 | 20 |
| 18873 | 25 |

**Supplementary Figure 51.** HPLC calibration curve for substrate **10c**.

**Assay**

**Supplementary Table 6.** HPLC-based analysis of substrate consumption (**10c**) by AsqJ.

| **10c** | Entry 1 | | | Entry 2 | | | Average Conversion [%] |
| --- | --- | --- | --- | --- | --- | --- | --- |
| t [min] | PA [mAU·s] | n [nmol] | Conversion [%] | PA [mAU·s] | n [nmol] | Conversion [%] |  |
| 0 | 19191 | 26.396 | − | 19006 | 25.619 | − | − |
| 15 | 17555 | 20.320 | 23.0 | 17201 | 19.222 | 25.0 | 24.0 |
| 60 | 7824 | 5.325 | 79.8 | 8094 | 5.530 | 78.4 | 79.1 |
| 180 | 118* | 0.161 | 99.4 | 167* | 0.186 | 99.3 | 99.3 |
| 360 | n/a | n/a | >99.0 | n/a | n/a | >99.0 | >99.0 |

*HPLC peak overlap with signal of product **30c**.

**Supplementary Figure 52.** Substrate (**10c**) consumption by AsqJ over reaction time.

1,4-dimethyl-3,4-dihydro-1*H*-benzo[1,4]diazepine-2,5-dione (**49n**)

**Calibration**

**Supplementary Table 7.** HPLC calibration values for substrate **49n**.

| Peak Area [mAU·s] | n [nmol] |
| --- | --- |
| 846 | 0.625 |
| 1698 | 1.25 |
| 3354 | 2.5 |
| 6492 | 5 |
| 10875 | 10 |
| 13724 | 15 |
| 14999 | 20 |
| 16727 | 25 |

**Supplementary Figure 53.** HPLC calibration curve for substrate **49n**.

**Assay**

**Supplementary Table 8.** HPLC-based analysis of substrate consumption (**49n**) by AsqJ.

| **49n** | Entry 1 | | | Entry 2 | | | Average Conversion [%] |
| --- | --- | --- | --- | --- | --- | --- | --- |
| t [min] | PA [mAU·s] | n [nmol] | Conversion [%] | PA [mAU·s] | n [nmol] | Conversion [%] |  |
| 0 | 14477 | 17.436 | − | 14644 | 17.885 | − | − |
| 15 | 13906 | 15.982 | 8.3 | 13834 | 15.808 | 11.6 | 10.0 |
| 60 | 12254 | 12.410 | 28.8 | 11315 | 10.749 | 39.9 | 34.4 |
| 180 | 5654 | 4.477 | 74.3 | 2595 | 2.143 | 88.0 | 81.2 |
| 360 | 1188 | 0.926 | 94.7 | 843 | 0.596 | 96.7 | 95.7 |

**Supplementary Figure 54.** Substrate (**49n**) consumption by AsqJ over reaction time.

(*S*)-1,2,3,11a-tetrahydro-5*H*-benzo[e]pyrrolo[1,2-*a*][1,4]diazepine-5,11(10*H*)-dione (**54**)

**Calibration**

**Supplementary Table 9.** HPLC calibration values for substrate **54**.

| Peak Area [mAU·s] | n [nmol] |
| --- | --- |
| 496 | 0.625 |
| 995 | 1.25 |
| 1984 | 2.5 |
| 3869 | 5 |
| 7313 | 10 |
| 10312 | 15 |
| 11648 | 20 |
| 13593 | 25 |

**Supplementary Figure 55.** HPLC calibration curve for substrate **54**.

**Assay**

**Supplementary Table 10.** HPLC-based analysis of substrate consumption (**54**) by AsqJ.

| **54** | Entry 1 | | | Entry 2 | | | Average Conversion [%] |
| --- | --- | --- | --- | --- | --- | --- | --- |
| t [min] | PA [mAU·s] | n [nmol] | Conversion [%] | PA [mAU·s] | n [nmol] | Conversion [%] |  |
| 0 | 11943 | 17.417 | − | 12161 | 18.026 | − | − |
| 15 | 11931 | 17.384 | 0.2 | 12146 | 17.983 | 0.2 | 0.2 |
| 60 | 11208 | 15.485 | 11.1 | 11635 | 16.585 | 8.0 | 9.5 |
| 180 | 9265 | 11.250 | 35.4 | 10390 | 13.552 | 24.8 | 30.1 |
| 360 | 7989 | 9.094 | 47.8 | 8514 | 9.926 | 44.9 | 46.4 |

**Supplementary Figure 56.** Substrate (**54**) consumption by AsqJ over reaction time.

**Comparison of Substrate Consumptions**

**Supplementary Table 11.** Comparison of substrate consumption metrics.

| **Substrate** | **9c** | **10c** | **49n** | **54** |
| --- | --- | --- | --- | --- |
| Effective enzyme loading [mol-%] | 1.88 | 1.92 | 2.83 | 2.82 |
| *t*_(50% conversion)_ [min] | ~118 | ~36 | ~99 | n/a |
| Conversion after 6 hours [%] | 88 | >99 | 96 | 46 |

**Supplementary Figure 57.** Comparison of investigated substrate consumptions.

# **4. Additional Schemes**

**Established Mechanism towards Quinolones**

**Supplementary Figure 58.** Mechanism of desaturation of natural substrate **3**, catalysed by AsqJ.^3, 12^ The mechanism starts with radical formation at *C^10^*.

**Supplementary Figure 59.** Mechanisms of epoxidation^13^ and fragmentation^3^ of desaturated compound **SI-31** to quinolone **4**. For the epoxidation step, a carbocation intermediate after electrophilic attack of Fe^IV^ at the double bond (not shown here) has also been proposed.^13^ B: Basic residue in the AsqJ active site.

**Mechanism towards Quinazolinones**

**Supplementary Figure 60.** Top left: formation of quinazolin-2,4-dione **22**, see Figure S45; top right: mechanism of α-lactam formation starting with *C^3^–H*-abstraction. Bottom: mechanism of subsequent hydrolysis of **SI‑36** and oxidative decarboxylation towards **21**.^2, 14^ Succinate and aspartate residues are modelled as acetate groups and histidine residues are modelled as methyl imidazoles.

**Proposals for Novel Mechanism towards Quinazolindiones**

**Supplementary Figure 61.** Mechanism proposals of quinazolin-2,4-dione (**22**) formation, starting from radical **SI-35**. a) *Via* cyclic intermediates; concerted and sequential proposal converge in **SI-41**, rearrangement with elimination of an aldehyde yields ring-contracted heterocycle **22** (B: Basic residue in the AsqJ active site).
b) *Via* alcohol **SI-42** and linear intermediate **SI-43**; nucleophilic acyl substitution of the secondary amine over **SI‑44** (similar to **SI-41**) yields quinazolindione **22**.

# **5. Selected UV Spectra**

218

206


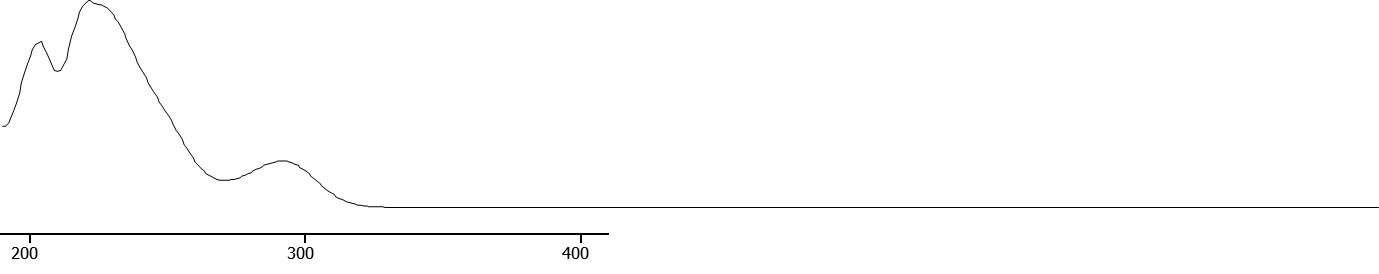


[nm]

292

**Supplementary Figure 62.** UV spectrum of (*S*)-9-fluoro-3-isobutyl-4-methyl-3,4-dihydro-1*H*-benzo[1,4]diazepine-2,5-dione (**9d**).

221


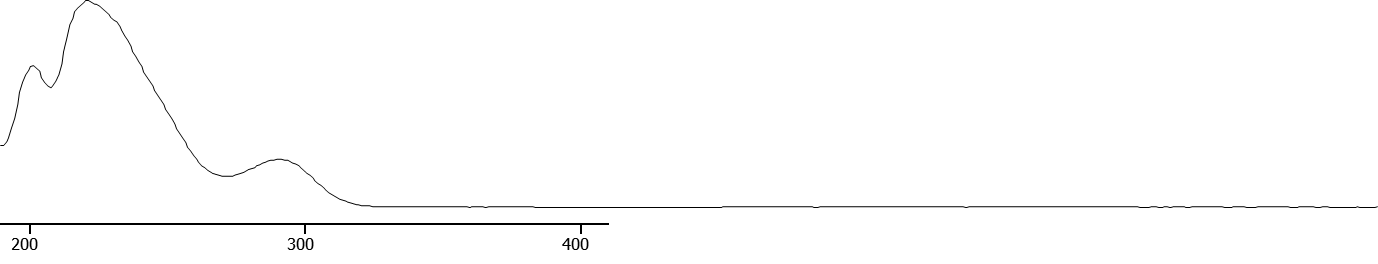


291

202

[nm]

**Supplementary Figure 63.** UV spectrum of (*S*)-3-benzyl-9-fluoro-4-methyl-3,4-dihydro-1*H*-benzo[1,4]diazepine-2,5-dione (**10d**).

201

228


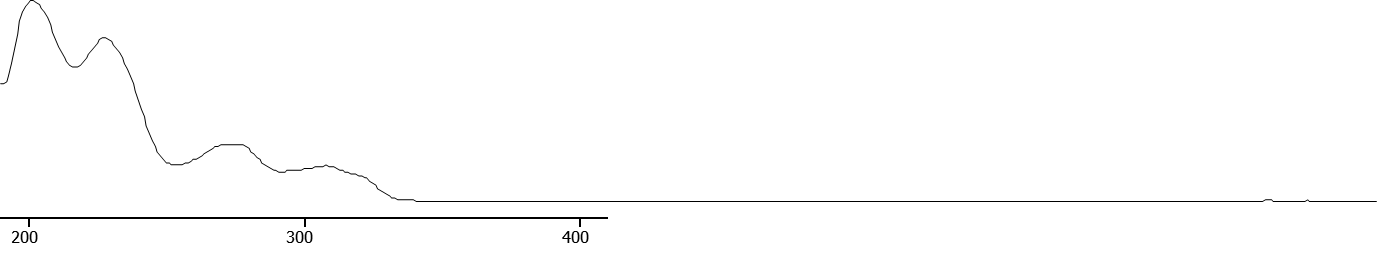


309

275

[nm]

**Supplementary Figure 64.** UV spectrum of 8-fluoro-2-isobutyl-3-methylquinazolin-4(3H)-one (**21d**).

217


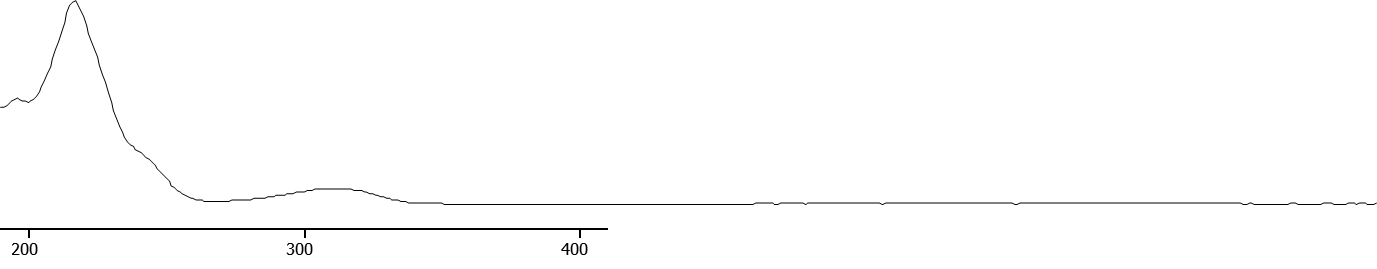


[nm]

311

244

**Supplementary Figure 65.** UV spectrum of 8-fluoro-3-methylquinazoline-2,4(1*H*,3*H*)-dione (**22d**).

222

241


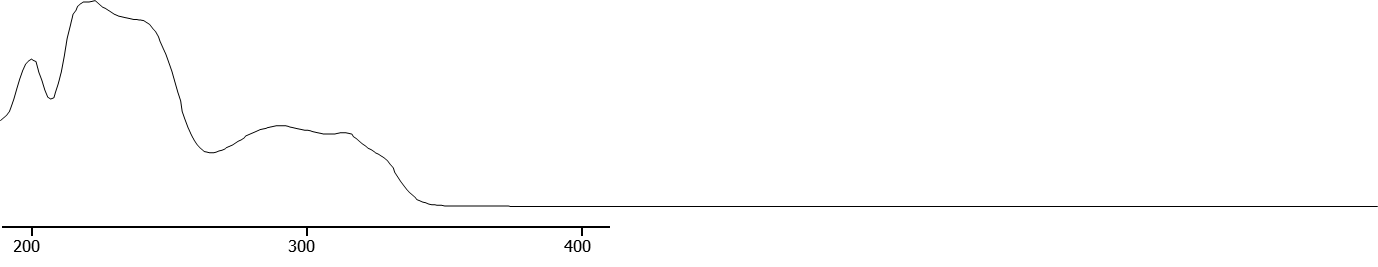


316

292

[nm]

201

**Supplementary Figure 66.** UV spectrum of 8-fluoro-3-hydroxy-4-phenylquinolin-2(1*H*)-one (**28d**).

215


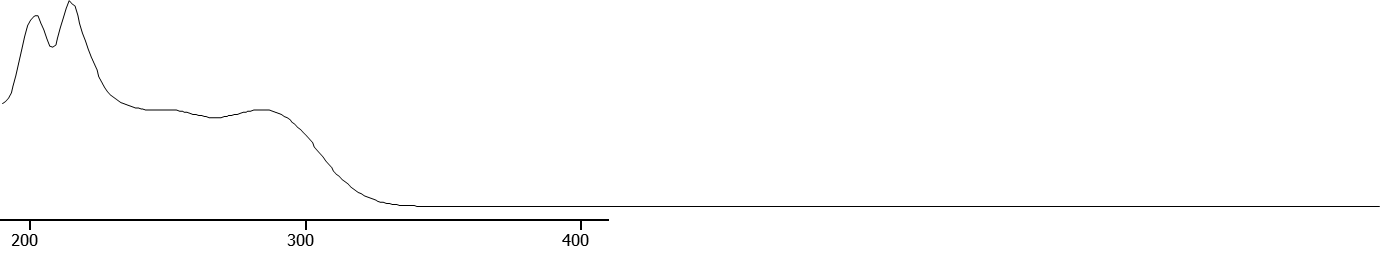


254

286

202

[nm]

**Supplementary Figure 67.** UV spectrum of (*Z*)-3-benzylidene-9-fluoro-4-methyl-3,4-dihydro-1*H*-benzo[1,4]diazepine-2,5-dione (**29d**).

220


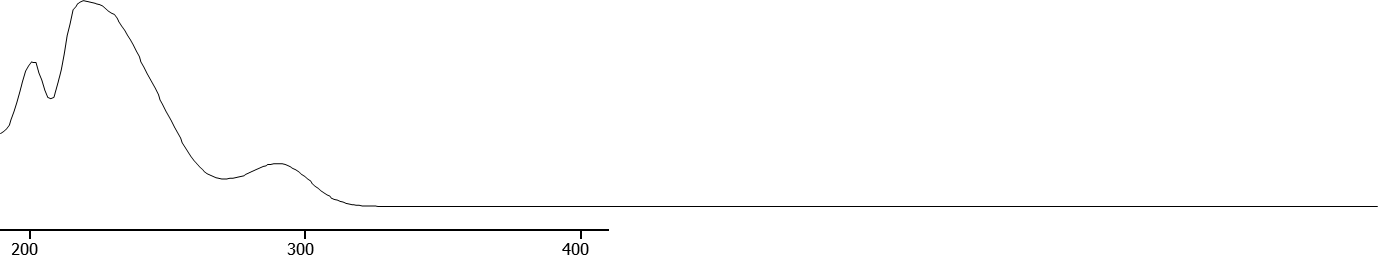


291

202

[nm]

**Supplementary Figure 68.** UV spectrum of (3'*S*)-9-fluoro-4-methyl-3'-phenylspiro[benzo[1,4]diazepine-3,2'-oxirane]-2,5(1*H*,4*H*)-dione (**30d**).

218

202


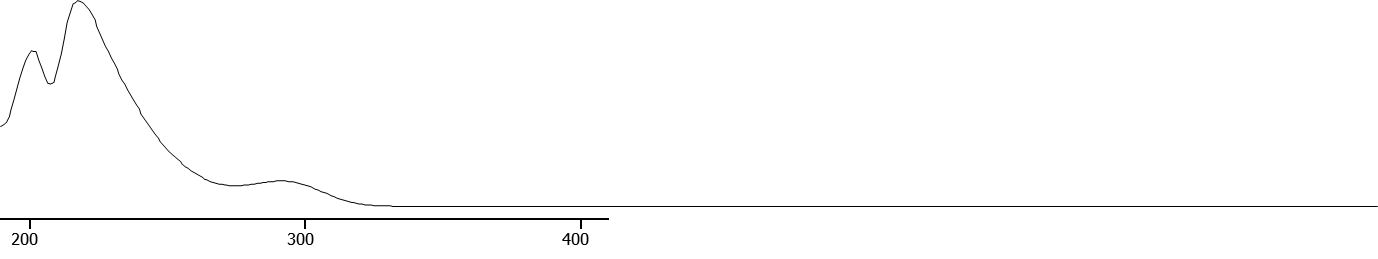


293

[nm]

**Supplementary Figure 69.** UV spectrum of 9-fluoro-3-hydroxy-3-(hydroxy(phenyl)methyl)-4-methyl-3,4-dihydro-1*H*-benzo[1,4]diazepine-2,5-dione (**31d**).

2018


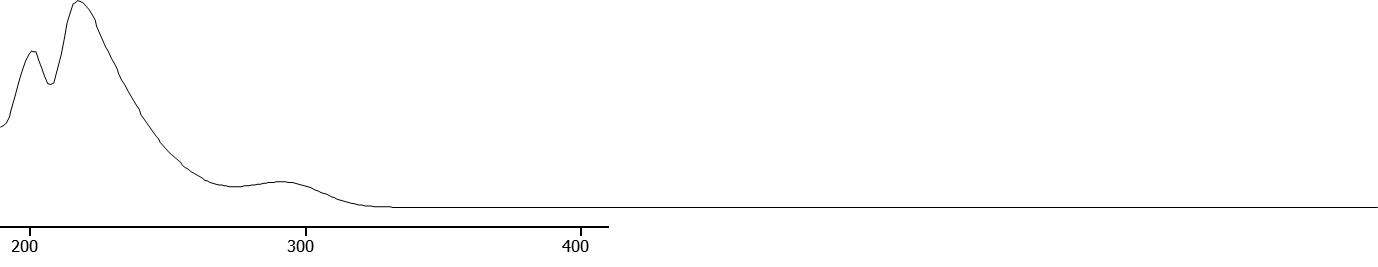


[nm]

288

202

**Supplementary Figure 70.** UV spectrum of 3-hydroxy-1,4-dimethyl-3,4-dihydro-1*H*-benzo[1,4]diazepine-2,5-dione (**50**).

200

228


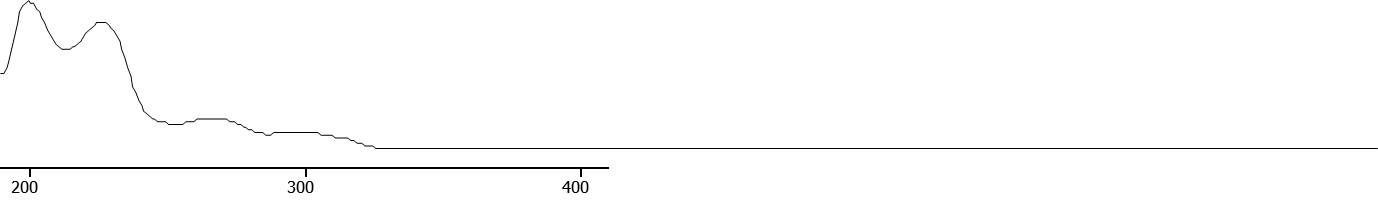


298

267

[nm]

**Supplementary Figure 71.** UV spectrum of (*R*)-2-hydroxy-2,3-dihydropyrrolo[2,1-*b*]quinazolin-9(1*H*)-one (**66**).

# **6. NMR Data**

**Oxazolidinones**

**Supplementary Figure 72.** ^1^H-NMR spectrum of (9*H*-fluoren-9-yl)methyl (*S*)-4-isobutyl-5-oxooxazolidine-3-carboxylate (**13a**). Ethyl acetate was not removable due to high viscosity of the product.

**Supplementary Figure 73.** ^13^C-NMR spectrum of (9*H*-fluoren-9-yl)methyl (*S*)-4-isobutyl-5-oxooxazolidine-3-carboxylate (**13a**).

**Supplementary Figure 74.** ^1^H-NMR spectrum of (9*H*-fluoren-9-yl)methyl (*S*)-4-benzyl-5-oxooxazolidine-3-carboxylate (**13b**).

Ethyl acetate was not removable due to high viscosity of the product.

**Supplementary Figure 75.** ^13^C-NMR spectrum of (9*H*-fluoren-9-yl)methyl (*S*)-4-benzyl-5-oxooxazolidine-3-carboxylate (**13b**).

**Fmoc-*N*-methyl amino acids**

**Supplementary Figure 76.** ^1^H-NMR spectrum of *N*-(((9*H*-fluoren-9-yl)methoxy)carbonyl)-*N*-methyl-l-leucine (**11a**).

**Supplementary Figure 77.** ^13^C-NMR spectrum of *N*-(((9*H*-fluoren-9-yl)methoxy)carbonyl)-*N*-methyl-l-leucine (**11a**).

**Figure S78.** ^1^H-NMR spectrum of *N*-(((9*H*-fluoren-9-yl)methoxy)carbonyl)-*N*-methyl-l-phenylalanine (**11b**).

**Figure S79.** ^13^C-NMR spectrum of *N*-(((9*H*-fluoren-9-yl)methoxy)carbonyl)-*N*-methyl-l-phenylalanine (**11b**).

***N*-Fmoc-amino acids**

**Supplementary Figure 80.** ^1^H-NMR spectrum of 2-((((9*H*-fluoren-9-yl)methoxy)carbonyl)amino)-6-fluorobenzoic acid (**16a**). The large signal at 3.58 ppm represents residual dioxane.

**Supplementary Figure 81.** ^13^C-NMR spectrum of 2-((((9*H*-fluoren-9-yl)methoxy)carbonyl)amino)-6-fluorobenzoic acid (**16a**). The large signal at 67.6 ppm represents residual dioxane.

**Supplementary Figure 82.** ^19^F-NMR spectrum of 2-((((9*H*-fluoren-9-yl)methoxy)carbonyl)amino)-6-fluorobenzoic acid (**16a**).

**Supplementary Figure 83.** ^1^H-NMR spectrum of 2-((((9*H*-fluoren-9-yl)methoxy)carbonyl)amino)-5-fluorobenzoic acid (**16b**). The large signal at 3.58 ppm represents residual dioxane.

**Supplementary Figure 84.** ^13^C-NMR spectrum of 2-((((9*H*-fluoren-9-yl)methoxy)carbonyl)amino)-5-fluorobenzoic acid (**16b**).

**Supplementary Figure 85.** ^19^F-NMR spectrum of 2-((((9*H*-fluoren-9-yl)methoxy)carbonyl)amino)-5-fluorobenzoic acid (**16b**).

**Supplementary Figure 86.** ^1^H-NMR spectrum of 2-((((9*H*-fluoren-9-yl)methoxy)carbonyl)amino)-4-fluorobenzoic acid (**16c**).

**Supplementary Figure 87.** ^13^C-NMR spectrum of 2-((((9*H*-fluoren-9-yl)methoxy)carbonyl)amino)-4-fluorobenzoic acid (**16c**).

**Supplementary Figure 88.** ^19^F-NMR spectrum of 2-((((9*H*-fluoren-9-yl)methoxy)carbonyl)amino)-4-fluorobenzoic acid (**16c**).

**Supplementary Figure 89.** ^1^H-NMR spectrum of 2-((((9*H*-fluoren-9-yl)methoxy)carbonyl)amino)-3-fluorobenzoic acid (**16d**).

**Supplementary Figure 90.** ^13^C-NMR spectrum of 2-((((9*H*-fluoren-9-yl)methoxy)carbonyl)amino)-3-fluorobenzoic acid (**16d**).

**Supplementary Figure 91.** ^19^F-NMR spectrum of 2-((((9*H*-fluoren-9-yl)methoxy)carbonyl)amino)-3-fluorobenzoic acid (**16d**).

**Supplementary Figure 92.** ^1^H-NMR spectrum of 2-((((9*H*-fluoren-9-yl)methoxy)carbonyl)amino)-6-chlorobenzoic acid (**16e**). The large signal at 3.58 ppm represents residual dioxane.

**Supplementary Figure 93.** ^13^C-NMR spectrum of 2-((((9*H*-fluoren-9-yl)methoxy)carbonyl)amino)-6-chlorobenzoic acid (**16e**).

**Supplementary Figure 94.** ^1^H-NMR spectrum of 2-((((9*H*-fluoren-9-yl)methoxy)carbonyl)amino)-5-chlorobenzoic acid (**16f**).

**Supplementary Figure 95.** ^13^C-NMR spectrum of 2-((((9*H*-fluoren-9-yl)methoxy)carbonyl)amino)-5-chlorobenzoic acid (**16f**).

**Supplementary Figure 96.** ^1^H-NMR spectrum of 2-((((9*H*-fluoren-9-yl)methoxy)carbonyl)amino)-4-chlorobenzoic acid (**16g**).

**Supplementary Figure 97.** ^13^C-NMR spectrum of 2-((((9*H*-fluoren-9-yl)methoxy)carbonyl)amino)-4-chlorobenzoic acid (**16g**).

**Supplementary Figure 98.** ^1^H-NMR spectrum of 2-((((9*H*-fluoren-9-yl)methoxy)carbonyl)amino)-4-nitrobenzoic acid (**16h**).

**Supplementary Figure 99.** ^13^C-NMR spectrum of 2-((((9*H*-fluoren-9-yl)methoxy)carbonyl)amino)-4-nitrobenzoic acid (**16h**).

**Supplementary Figure 100.** ^1^H-NMR spectrum of 2-((((9*H*-fluoren-9-yl)methoxy)carbonyl)amino)-6-methylbenzoic acid (**16i**). The large signal at 3.58 ppm represents residual dioxane.

**Supplementary Figure 101.** ^13^C-NMR spectrum of 2-((((9*H*-fluoren-9-yl)methoxy)carbonyl)amino)-6-methylbenzoic acid (**16i**). The large signal at 67.6 ppm represents residual dioxane.

**Supplementary Figure 102.** ^1^H-NMR spectrum of 2-((((9*H*-fluoren-9-yl)methoxy)carbonyl)amino)-5-methylbenzoic acid (**16j**). The signal at 3.58 ppm represents residual dioxane.

**Supplementary Figure 103.** ^13^C-NMR spectrum of 2-((((9*H*-fluoren-9-yl)methoxy)carbonyl)amino)-5-methylbenzoic acid (**16j**).

**Supplementary Figure 104.** ^1^H-NMR spectrum of 2-((((9*H*-fluoren-9-yl)methoxy)carbonyl)amino)-3-methylbenzoic acid (**16k**).

**Supplementary Figure 105.** ^13^C-NMR spectrum of 2-((((9*H*-fluoren-9-yl)methoxy)carbonyl)amino)-3-methylbenzoic acid (**16k**).

**Supplementary Figure 106.** ^1^H-NMR spectrum of 3-((((9*H*-fluoren-9-yl)methoxy)carbonyl)amino)-2-naphthoic acid (**16l**).

**Supplementary Figure 107.** ^13^C-NMR spectrum of 3-((((9*H*-fluoren-9-yl)methoxy)carbonyl)amino)-2-naphthoic acid (**16l**).

**Supplementary Figure 108.** ^1^H-NMR spectrum of (2*S*,4*R*)-1-(((9*H*-fluoren-9-yl)methoxy)carbonyl)-4-hydroxypyrrolidine-2-carboxylic acid (**SI-1**). The large signal at 3.58 ppm represents residual dioxane.

**Supplementary Figure 109.** ^13^C-NMR spectrum of (2*S*,4*R*)-1-(((9*H*-fluoren-9-yl)methoxy)carbonyl)-4-hydroxypyrrolidine-2-carboxylic acid (**SI-1**). The large signal at 67.6 ppm represents residual dioxane.

**Supplementary Figure 110.** ^1^H-NMR spectrum of (2*S*,4*R*)-1-(((9*H*-fluoren-9-yl)methoxy)carbonyl)-4-((*tert*-butyldimethylsilyl)oxy)pyrrolidine-2-carboxylic acid (**68**).

**Supplementary Figure 111.** ^13^C-NMR spectrum of (2*S*,4*R*)-1-(((9*H*-fluoren-9-yl)methoxy)carbonyl)-4-((*tert*-butyldimethylsilyl)oxy)pyrrolidine-2-carboxylic acid (**68**).

**Linear Precursors**

**Supplementary Figure 112.** ^1^H-NMR spectrum of *N*-(2-hydroxybenzoyl)-*N*-methyl-l-leucine (**88a**).

**Supplementary Figure 113.** ^13^C-NMR spectrum of *N*-(2-hydroxybenzoyl)-*N*-methyl-l-leucine (**88a**).

**Supplementary Figure 114.** ^1^H-NMR spectrum of *N*-(2-hydroxybenzoyl)-*N*-methyl-l-phenylalanine (**88b**).

**Supplementary Figure 115.** ^13^C-NMR spectrum of *N*-(2-hydroxybenzoyl)-*N*-methyl-l-phenylalanine (**88b**).

**Supplementary Figure 116.** ^1^H-NMR spectrum of methyl (*S*)-2-hydroxy-4-methylpentanoate (**81a**).

**Supplementary Figure 117.** ^1^H-NMR spectrum of methyl (*S*)-2-hydroxy-3-phenylpropanoate (**81b**).

**Supplementary Figure 118.** ^1^H-NMR spectrum of (*S*)-1-methoxy-4-methyl-1-oxopentan-2-yl 2-nitrobenzoate (**82a**).

**Supplementary Figure 119.** ^13^C-NMR spectrum of (*S*)-1-methoxy-4-methyl-1-oxopentan-2-yl 2-nitrobenzoate (**82a**).

**Supplementary Figure 120.** ^1^H-NMR spectrum of (*S*)-1-methoxy-1-oxo-3-phenylpropan-2-yl 2-nitrobenzoate (**82b**).

**Supplementary Figure 121.** ^13^C-NMR spectrum of (*S*)-1-methoxy-1-oxo-3-phenylpropan-2-yl 2-nitrobenzoate (**82b**).

**Supplementary Figure 122.** ^1^H-NMR spectrum of (*S*)-4-methyl-2-((2-nitrobenzoyl)oxy)pentanoic acid (**83a**).

**Supplementary Figure 123.** ^13^C-NMR spectrum of (*S*)-4-methyl-2-((2-nitrobenzoyl)oxy)pentanoic acid (**83a**).

**Supplementary Figure 124.** ^1^H-NMR spectrum of (*S*)-2-((2-nitrobenzoyl)oxy)-3-phenylpropanoic acid (**83b**).

**Supplementary Figure 125.** ^13^C-NMR spectrum of (*S*)-2-((2-nitrobenzoyl)oxy)-3-phenylpropanoic acid (**83b**).

**Supplementary Figure 126.** ^1^H-NMR spectrum of (*S*)-2-((2-aminobenzoyl)oxy)-4-methylpentanoic acid (**84a**).

**Supplementary Figure 127.** ^13^C-NMR spectrum of (*S*)-2-((2-aminobenzoyl)oxy)-4-methylpentanoic acid (**84a**).

**Supplementary Figure 128.** ^1^H-NMR spectrum of (*S*)-2-((2-aminobenzoyl)oxy)-3-phenylpropanoic acid (**84b**).

**Supplementary Figure 129.** ^13^C-NMR spectrum of (*S*)-2-((2-aminobenzoyl)oxy)-3-phenylpropanoic acid (**84b**).

**Substrates**

**Supplementary Figure 130.** ^1^H-NMR spectrum of (*R*)-3-isobutyl-4-methyl-3,4-dihydro-1*H*-benzo[1,4]diazepine-2,5-dione ((*R*)‑**9**).

**Supplementary Figure 131.** ^13^C-NMR spectrum of (*R*)-3-isobutyl-4-methyl-3,4-dihydro-1*H*-benzo[1,4]diazepine-2,5-dione ((*R*)‑**9**).

**Supplementary Figure 132.** ^1^H-NMR spectrum of (*S*)-6-fluoro-3-isobutyl-4-methyl-3,4-dihydro-1*H*-benzo[1,4]diazepine-2,5-dione (**9a**).

**Supplementary Figure 133.** ^13^C-NMR spectrum of (*S*)-6-fluoro-3-isobutyl-4-methyl-3,4-dihydro-1*H*-benzo[1,4]diazepine-2,5-dione (**9a**).

**Supplementary Figure 134.** ^19^F-NMR spectrum of (*S*)-6-fluoro-3-isobutyl-4-methyl-3,4-dihydro-1*H*-benzo[1,4]diazepine-2,5-dione (**9a**).

**Supplementary Figure 135.** ^1^H-NMR spectrum of (*S*)-7-fluoro-3-isobutyl-4-methyl-3,4-dihydro-1*H*-benzo[1,4]diazepine-2,5-dione (**9b**).

**Supplementary Figure 136.** ^13^C-NMR spectrum of (*S*)-7-fluoro-3-isobutyl-4-methyl-3,4-dihydro-1*H*-benzo[1,4]diazepine-2,5-dione (**9b**).

**Supplementary Figure 137.** ^19^F-NMR spectrum of (*S*)-7-fluoro-3-isobutyl-4-methyl-3,4-dihydro-1*H*-benzo[1,4]diazepine-2,5-dione (**9b**).

**Supplementary Figure 138.** ^1^H-NMR spectrum of (*S*)-8-fluoro-3-isobutyl-4-methyl-3,4-dihydro-1*H*-benzo[1,4]diazepine-2,5-dione (**9c**).

**Supplementary Figure 139.** ^13^C-NMR spectrum of (*S*)-8-fluoro-3-isobutyl-4-methyl-3,4-dihydro-1*H*-benzo[1,4]diazepine-2,5-dione (**9c**).

**Supplementary Figure 140.** ^19^F-NMR spectrum of (*S*)-8-fluoro-3-isobutyl-4-methyl-3,4-dihydro-1*H*-benzo[1,4]diazepine-2,5-dione (**9c**).

**Supplementary Figure 141.** ^1^H-NMR spectrum of (*S*)-9-fluoro-3-isobutyl-4-methyl-3,4-dihydro-1*H*-benzo[1,4]diazepine-2,5-dione (**9d**).

**Supplementary Figure 142.** ^13^C-NMR spectrum of (*S*)-9-fluoro-3-isobutyl-4-methyl-3,4-dihydro-1*H*-benzo[1,4]diazepine-2,5-dione (**9d**).

**Supplementary Figure 143.** ^19^F-NMR spectrum of (*S*)-9-fluoro-3-isobutyl-4-methyl-3,4-dihydro-1*H*-benzo[1,4]diazepine-2,5-dione (**9d**).

**Supplementary Figure 144.** ^1^H-NMR spectrum of (*S*)-6-chloro-3-isobutyl-4-methyl-3,4-dihydro-1*H*-benzo[1,4]diazepine-2,5-dione (**9e**). Minor signals represent inseparable impurities from substrate synthesis.

**Supplementary Figure 145.** ^13^C-NMR spectrum of (*S*)-6-chloro-3-isobutyl-4-methyl-3,4-dihydro-1*H*-benzo[1,4]diazepine-2,5-dione (**9e**).

**Supplementary Figure 146.** ^1^H-NMR spectrum of (*S*)-7-chloro-3-isobutyl-4-methyl-3,4-dihydro-1*H*-benzo[1,4]diazepine-2,5-dione (**9f**).

**Supplementary Figure 147.** ^13^C-NMR spectrum of (*S*)-7-chloro-3-isobutyl-4-methyl-3,4-dihydro-1*H*-benzo[1,4]diazepine-2,5-dione (**9f**).

**Supplementary Figure 148.** ^1^H-NMR spectrum of (*S*)-8-chloro-3-isobutyl-4-methyl-3,4-dihydro-1*H*-benzo[1,4]diazepine-2,5-dione (**9g**).

**Supplementary Figure 149.** ^13^C-NMR spectrum of (*S*)-8-chloro-3-isobutyl-4-methyl-3,4-dihydro-1*H*-benzo[1,4]diazepine-2,5-dione (**9g**).

**Supplementary Figure 150.** ^1^H-NMR spectrum of (*S*)-3-isobutyl-4,6-dimethyl-3,4-dihydro-1*H*-benzo[1,4]diazepine-2,5-dione (**9i**). Minor signals represent inseparable impurities from substrate synthesis.

**Supplementary Figure 151.** ^13^C-NMR spectrum of (*S*)-3-isobutyl-4,6-dimethyl-3,4-dihydro-1*H*-benzo[1,4]diazepine-2,5-dione (**9i**).

**Supplementary Figure 152.** ^1^H-NMR spectrum of (*S*)-3-isobutyl-4,7-dimethyl-3,4-dihydro-1*H*-benzo[1,4]diazepine-2,5-dione (**9j**).

**Supplementary Figure 153.** ^13^C-NMR spectrum of (*S*)-3-isobutyl-4,7-dimethyl-3,4-dihydro-1*H*-benzo[1,4]diazepine-2,5-dione (**9j**).

**Supplementary Figure 154.** ^1^H-NMR spectrum of (*S*)-3-isobutyl-4,9-dimethyl-3,4-dihydro-1*H*-benzo[1,4]diazepine-2,5-dione (**9k**).

**Supplementary Figure 155.** ^13^C-NMR spectrum of (*S*)-3-isobutyl-4,9-dimethyl-3,4-dihydro-1*H*-benzo[1,4]diazepine-2,5-dione (**9k**).

**Supplementary Figure 156.** ^1^H-NMR spectrum of (*S*)-3-isobutyl-4-methyl-3,4-dihydro-1*H*-naphtho[2,3‑*e*][1,4]diazepine-2,5-dione (**9l**).

**Supplementary Figure 157.** ^13^C-NMR spectrum of (*S*)-3-isobutyl-4-methyl-3,4-dihydro-1*H*-naphtho[2,3‑*e*][1,4]diazepine-2,5-dione (**9l**).

**Supplementary Figure 158.** ^1^H-NMR spectrum of (*S*)-4-ethyl-3-isobutyl-3,4-dihydro-1*H*-benzo[1,4]diazepine-2,5-dione (**9m**).

**Supplementary Figure 159.** ^13^C-NMR spectrum of (*S*)-4-ethyl-3-isobutyl-3,4-dihydro-1*H*-benzo[1,4]diazepine-2,5-dione (**9m**).

**Supplementary Figure 160.** ^1^H-NMR spectrum of (*R*)-3-benzyl-4-methyl-3,4-dihydro-1*H*-benzo[1,4]diazepine-2,5-dione ((*R*)‑**10**).

**Supplementary Figure 161.** ^13^C-NMR spectrum of (*R*)-3-benzyl-4-methyl-3,4-dihydro-1*H*-benzo[1,4]diazepine-2,5-dione ((*R*)‑**10**).

**Supplementary Figure 162.** ^1^H-NMR spectrum of (*S*)-3-benzyl-6-fluoro-4-methyl-3,4-dihydro-1*H*-benzo[1,4]diazepine-2,5-dione (**10a**).

**Supplementary Figure 163.** ^13^C-NMR spectrum of (*S*)-3-benzyl-6-fluoro-4-methyl-3,4-dihydro-1*H*-benzo[1,4]diazepine-2,5-dione (**10a**).

**Supplementary Figure 164.** ^19^F-NMR spectrum of (*S*)-3-benzyl-6-fluoro-4-methyl-3,4-dihydro-1*H*-benzo[1,4]diazepine-2,5-dione (**10a**).

**Supplementary Figure 165.** ^1^H-NMR spectrum of (*S*)-3-benzyl-7-fluoro-4-methyl-3,4-dihydro-1*H*-benzo[1,4]diazepine-2,5-dione (**10b**).

**Supplementary Figure 166.** ^13^C-NMR spectrum of (*S*)-3-benzyl-7-fluoro-4-methyl-3,4-dihydro-1*H*-benzo[1,4]diazepine-2,5-dione (**10b**).

**Supplementary Figure 167.** ^19^F-NMR spectrum of (*S*)-3-benzyl-7-fluoro-4-methyl-3,4-dihydro-1*H*-benzo[1,4]diazepine-2,5-dione (**10b**).

**Supplementary Figure 168.** ^1^H-NMR spectrum of (*S*)-3-benzyl-8-fluoro-4-methyl-3,4-dihydro-1*H*-benzo[1,4]diazepine-2,5-dione (**10c**).

**Supplementary Figure 169.** ^13^C-NMR spectrum of (*S*)-3-benzyl-8-fluoro-4-methyl-3,4-dihydro-1*H*-benzo[1,4]diazepine-2,5-dione (**10c**).

**Supplementary Figure 170.** ^19^F-NMR spectrum of (*S*)-3-benzyl-8-fluoro-4-methyl-3,4-dihydro-1*H*-benzo[1,4]diazepine-2,5-dione (**10c**).

**Supplementary Figure 171.** ^1^H-NMR spectrum of (*S*)-3-benzyl-9-fluoro-4-methyl-3,4-dihydro-1*H*-benzo[1,4]diazepine-2,5-dione (**10d**).

**Supplementary Figure 172.** ^13^C-NMR spectrum of (*S*)-3-benzyl-9-fluoro-4-methyl-3,4-dihydro-1*H*-benzo[1,4]diazepine-2,5-dione (**10d**).

**Supplementary Figure 173.** ^19^F-NMR spectrum of (*S*)-3-benzyl-9-fluoro-4-methyl-3,4-dihydro-1*H*-benzo[1,4]diazepine-2,5-dione (**10d**).

**Supplementary Figure 174.** ^1^H-NMR spectrum of (*S*)-3-benzyl-6-chloro-4-methyl-3,4-dihydro-1*H*-benzo[1,4]diazepine-2,5-dione (**10e**). Minor signals represent inseparable impurities from substrate synthesis.

**Supplementary Figure 175.** ^13^C-NMR spectrum of (*S*) 3-benzyl-6-chloro-4-methyl-3,4-dihydro-1*H*-benzo[1,4]diazepine-2,5-dione (**10e**).

**Supplementary Figure 176.** ^1^H-NMR spectrum of (*S*)-3-benzyl-7-chloro -4-methyl-3,4-dihydro-1*H*-benzo[1,4]diazepine-2,5-dione (**10f**).

**Supplementary Figure 177.** ^13^C-NMR spectrum of (*S*)-3-benzyl-7-chloro -4-methyl-3,4-dihydro-1*H*-benzo[1,4]diazepine-2,5-dione (**10f**).

**Supplementary Figure 178.** ^1^H-NMR spectrum of (*S*)-3-benzyl-8-chloro -4-methyl-3,4-dihydro-1*H*-benzo[1,4]diazepine-2,5-dione (**10g**).

**Supplementary Figure 179.** ^13^C-NMR spectrum of (*S*)-3-benzyl-8-chloro -4-methyl-3,4-dihydro-1*H*-benzo[1,4]diazepine-2,5-dione (**10g**).

**Supplementary Figure 180.** ^1^H-NMR spectrum of (*S*)-3-benzyl-4,6-dimethyl-3,4-dihydro-1*H*-benzo[1,4]diazepine-2,5-dione (**10i**).

**Supplementary Figure 181.** ^13^C-NMR spectrum of (*S*)-3-benzyl-4,6-dimethyl-3,4-dihydro-1*H*-benzo[1,4]diazepine-2,5-dione (**10i**).

**Supplementary Figure 182.** ^1^H-NMR spectrum of (*S*)-3-benzyl-4,7-dimethyl-3,4-dihydro-1*H*-benzo[1,4]diazepine-2,5-dione (**10j**).

**Supplementary Figure 183.** ^13^C-NMR spectrum of (*S*)-3-benzyl-4,7-dimethyl-3,4-dihydro-1*H*-benzo[1,4]diazepine-2,5-dione (**10j**).

**Supplementary Figure 184.** ^1^H-NMR spectrum of (*S*)-3-benzyl-4,9-dimethyl-3,4-dihydro-1*H*-benzo[1,4]diazepine-2,5-dione (**10k**).

**Supplementary Figure 185.** ^13^C-NMR spectrum of (*S*)-3-benzyl-4,9-dimethyl-3,4-dihydro-1*H*-benzo[1,4]diazepine-2,5-dione (**10k**).

**Supplementary Figure 186.** ^1^H-NMR spectrum of (*S*)-3-benzyl-4-methyl-3,4-dihydro-1*H*-naphtho[2,3-*e*][1,4]diazepine-2,5-dione (**10l**). Minor signals represent inseparable impurities from substrate synthesis.

**Supplementary Figure 187.** ^13^C-NMR spectrum of (*S*)-3-benzyl-4-methyl-3,4-dihydro-1*H*-naphtho[2,3-*e*][1,4]diazepine-2,5-dione (**10l**).

**Supplementary Figure 188.** ^1^H-NMR spectrum of (*S*)-4-ethyl-3-benzyl-3,4-dihydro-1*H*-benzo[1,4]diazepine-2,5-dione (**10m**).

**Supplementary Figure 189.** ^13^C-NMR spectrum of (*S*)-4-ethyl-3-benzyl-3,4-dihydro-1*H*-benzo[1,4]diazepine-2,5-dione (**10m**).

**Supplementary Figure 190.** ^1^H-NMR spectrum of (*S*)-3-(cyclohexylmethyl)-4-ethyl-3,4-dihydro-1*H*-benzo[1,4]diazepine-2,5-dione (**33**).

**Supplementary Figure 191.** ^13^C-NMR spectrum of (*S*)-3-(cyclohexylmethyl)-4-ethyl-3,4-dihydro-1*H*-benzo[1,4]diazepine-2,5-dione (**33**).

**Supplementary Figure 192.** ^1^H-NMR spectrum of (*S*)-4-ethyl-3-(4-methoxybenzyl)-3,4-dihydro-1*H*-benzo[1,4]diazepine-2,5-dione (**34**).

**Supplementary Figure 193.** ^13^C-NMR spectrum of (*S*)-4-ethyl-3-(4-methoxybenzyl)-3,4-dihydro-1*H*-benzo[1,4]diazepine-2,5-dione (**34**).

**Supplementary Figure 194.** ^1^H-NMR spectrum of (*S*)-4-ethyl-3-benzyl-3,4-dihydro-1*H*-benzo[1,4]diazepine-2,5-dione (**40**).

**Supplementary Figure 195.** ^13^C-NMR spectrum of (*S*)-4-ethyl-3-benzyl-3,4-dihydro-1*H*-benzo[1,4]diazepine-2,5-dione (**40**).

**Supplementary Figure 196.** ^1^H-NMR spectrum of (*S*)-1,2,3,11a-tetrahydro-5*H*-benzopyrrolo[1,2-*a*][1,4]diazepine-5,11(10*H*)-dione (**54**).

**Supplementary Figure 197.** ^13^C-NMR spectrum of (*S*)-1,2,3,11a-tetrahydro-5*H*-benzopyrrolo[1,2-*a*][1,4]diazepine-5,11(10*H*)-dione (**54**).

**Supplementary Figure 198.** ^1^H-NMR spectrum of (*S*)-7,8,9,10-tetrahydrobenzopyrido[1,2-a][1,4]diazepine-6,12(5*H*,6a*H*)-dione (**55**).

**Supplementary Figure 199.** ^13^C-NMR spectrum of (*S*)-7,8,9,10-tetrahydrobenzopyrido[1,2-a][1,4]diazepine-6,12(5*H*,6a*H*)-dione (**55**).

**Supplementary Figure 200.** ^1^H-NMR spectrum of (2*R*,11a*S*)-2-hydroxy-1,2,3,11a-tetrahydro-5*H*-benzopyrrolo[1,2‑*a*][1,4]diazepine-5,11(10*H*)-dione (**69**).

**Supplementary Figure 201.** ^13^C-NMR spectrum of (2*R*,11a*S*)-2-hydroxy-1,2,3,11a-tetrahydro-5*H*-benzopyrrolo[1,2‑*a*][1,4]diazepine-5,11(10*H*)-dione (**69**).

**Supplementary Figure 202.** ^1^H-NMR spectrum of (*S*)-3-isobutyl-4-methyl-8-nitro-3,4-dihydro-1*H*-benzo[1,4]diazepine-2,5-dione (**9h**).

**Supplementary Figure 203.** ^13^C-NMR spectrum of (*S*)-3-isobutyl-4-methyl-8-nitro-3,4-dihydro-1*H*-benzo[1,4]diazepine-2,5-dione (**9h**).

**Supplementary Figure 204.** ^1^H-NMR spectrum of (*S*)-3-benzyl-4-methyl-8-nitro-3,4-dihydro-1*H*-benzo[1,4]diazepine-2,5-dione (**10h**). Minor signals represent inseparable impurities from substrate synthesis.

**Supplementary Figure 205.** ^13^C-NMR spectrum of (*S*)-3-benzyl-4-methyl-8-nitro-3,4-dihydro-1*H*-benzo[1,4]diazepine-2,5-dione (**10h**).

**Supplementary Figure 206.** ^1^H-NMR spectrum of 1,4-dimethyl-3,4-dihydro-1*H*-benzo[1,4]diazepine-2,5-dione (**49n**).

**Supplementary Figure 207.** ^13^C-NMR spectrum of 1,4-dimethyl-3,4-dihydro-1*H*-benzo[1,4]diazepine-2,5-dione (**49n**).

**Supplementary Figure 208.** ^1^H-NMR spectrum of (*S*)-3-isobutyl-1,4-dimethyl-3,4-dihydro-1*H*-benzo[1,4]diazepine-2,5-dione (**9n**).

**Supplementary Figure 209.** ^13^C-NMR spectrum of (*S*)-3-isobutyl-1,4-dimethyl-3,4-dihydro-1*H*-benzo[1,4]diazepine-2,5-dione (**9n**).

**Supplementary Figure 210.** ^1^H-NMR spectrum of (*S*)-3-benzyl-1,4-dimethyl-3,4-dihydro-1*H*-benzo[1,4]diazepine-2,5-dione (**10n**).

**Supplementary Figure 211.** ^13^C-NMR spectrum of (*S*)-3-benzyl-1,4-dimethyl-3,4-dihydro-1*H*-benzo[1,4]diazepine-2,5-dione (**10n**).

**Supplementary Figure 212.** ^1^H-NMR spectrum of (*S*)-3-isobutylbenzo[1,4]oxazepine-2,5(1*H*,3*H*)-dione (**79a**).

**Supplementary Figure 213.** ^13^C-NMR spectrum of (*S*)-3-isobutylbenzo[1,4]oxazepine-2,5(1*H*,3*H*)-dione (**79a**).

**Supplementary Figure 214.** ^1^H-NMR spectrum of (*S*)-3-benzylbenzo[1,4]oxazepine-2,5(1*H*,3*H*)-dione (**79b**).

**Supplementary Figure 215.** ^13^C-NMR spectrum of (*S*)-3-benzylbenzo[1,4]oxazepine-2,5(1*H*,3*H*)-dione (**79b**).

**Supplementary Figure 216.** ^1^H-NMR spectrum of 2-amino-4-fluoro-*N*-methylbenzamide (**SI-19**).

**Supplementary Figure 217.** ^13^C-NMR spectrum of 2-amino-4-fluoro-*N*-methylbenzamide (**SI-19**).

**Supplementary Figure 218.** ^19^F-NMR spectrum of 2-amino-4-fluoro-*N*-methylbenzamide (**SI-19**).

**Supplementary Figure 219.** ^1^H-NMR spectrum of 7-fluoro-3-methylquinazoline-2,4(1*H*,3*H*)-dione (**22c**).

**Supplementary Figure 220.** ^13^C-NMR spectrum of 7-fluoro-3-methylquinazoline-2,4(1*H*,3*H*)-dione (**22c**).

**Supplementary Figure 221.** ^19^F-NMR spectrum of 7-fluoro-3-methylquinazoline-2,4(1*H*,3*H*)-dione (**22c**).

**Supplementary Figure 222.** ^1^H-NMR spectrum of 2-amino-*N*-(4-hydroxybutyl)benzamide (**SI-22**).

**Supplementary Figure 223.** ^13^C-NMR spectrum of 2-amino-*N*-(4-hydroxybutyl)benzamide (**SI-22**).

**Supplementary Figure 224.** ^1^H-NMR spectrum of 2-amino-*N*-(4-((*tert*-butyldimethylsilyl)oxy)butyl)benzamide (**SI-23**).

**Supplementary Figure 225.** ^13^C-NMR spectrum of 2-amino-*N*-(4-((*tert*-butyldimethylsilyl)oxy)butyl)benzamide (**SI-23**).

**Supplementary Figure 226.** ^1^H-NMR spectrum of 3-(4-((*tert*-butyldimethylsilyl)oxy)butyl)quinazoline-2,4(1*H*,3*H*)-dione (**SI-24**).

**Supplementary Figure 227.** ^13^C-NMR spectrum of 3-(4-((*tert*-butyldimethylsilyl)oxy)butyl)quinazoline-2,4(1*H*,3*H*)-dione (**SI-24**).

**Supplementary Figure 228.** ^1^H-NMR spectrum of 3-(4-hydroxybutyl)quinazoline-2,4(1*H*,3*H*)-dione (**SI-25**).

**Supplementary Figure 229.** ^13^C-NMR spectrum of 3-(4-hydroxybutyl)quinazoline-2,4(1*H*,3*H*)-dione (**SI-25**).

**Supplementary Figure 230.** ^1^H-NMR spectrum of 4-(2,4-dioxo-1,4-dihydroquinazolin-3(2*H*)-yl)butanal (**76**).

**Supplementary Figure 231.** ^13^C-NMR spectrum of 4-(2,4-dioxo-1,4-dihydroquinazolin-3(2*H*)-yl)butanal (**76**).

**Isolated Enzymatic Products**

**Supplementary Figure 232.** ^1^H-NMR spectrum of 7-fluoro-3-methylquinazoline-2,4(1*H*,3*H*)-dione (**22c**).

**Supplementary Figure 233.** ^19^F-NMR spectrum of 7-fluoro-3-methylquinazoline-2,4(1*H*,3*H*)-dione (**22c**).

**Supplementary Figure 234.** ^1^H-NMR spectrum of 7-fluoro-2-isobutyl-3-methylquinazolin-4(3*H*)-one (**21c**).

**Supplementary Figure 235.** ^19^F-NMR spectrum of 7-fluoro-2-isobutyl-3-methylquinazolin-4(3*H*)-one (**21c**).

**Supplementary Figure 236.** ^1^H-NMR spectrum of 3-methylquinazoline-2,4(1*H*,3*H*)-dione-2-^13^*C* (**25**).

**Supplementary Figure 237.** ^13^C-NMR spectrum of 3-methylquinazoline-2,4(1*H*,3*H*)-dione-2-^13^*C* (**25**). Only isotope-labelled carbon signal is visible due to low sample concentration.

**Supplementary Figure 238.** ^1^H-NMR spectrum of 3-hydroxy-1,4-dimethyl-3,4-dihydro-1*H*-benzo[1,4]diazepine-2,5-dione (**50**).

**Supplementary Figure 239.** ^1^H-NMR spectrum of 9-oxo-2,3,4,9-tetrahydropyrrolo[2,1-*b*]quinazoline-3a(1*H*)-carboxylic acid (**74**, presumed).

**Supplementary Figure 240.** ^1^H-NMR spectrum of 2,3-dihydropyrrolo[2,1-*b*]quinazolin-9(1*H*)-one (**64**).

**Supplementary Figure 241.** ^1^H-NMR spectrum of (*R*)-2-hydroxy-2,3-dihydropyrrolo[2,1-*b*]quinazolin-9(1*H*)-one (**66**).

**Supplementary Figure 242.** ^1^H-NMR spectrum of (*R*)-2-hydroxy-2,3-dihydropyrrolo[2,1-*b*]quinazolin-9(1*H*)-one (**66**), with water suppression.

**Supplementary Figure 243.** ^1^H–^13^C HSQC-NMR spectrum of (*R*)-2-hydroxy-2,3-dihydropyrrolo[2,1-*b*]quinazolin-9(1*H*)-one (**66**).

**Supplementary Figure 244.** ^1^H-NMR spectrum of 3-hydroxy-6-methyl-4-phenylquinolin-2(1*H*)-one (**28j**).

**Comparisons of NMR spectra (enzymatic *vs*. synthetic products)**

**Figure S245.** Comparison of ^1^H-NMR spectra of enzymatic (top) and synthetic (bottom) 7-fluoro-3-methylquinazoline-2,4(1H,3H)-dione (**22c**).

**Supplementary Figure 246.** Comparison of ^1^H-NMR spectra of enzymatic presumed 9-oxo-2,3,4,9-tetrahydropyrrolo[2,1‑*b*]quinazoline-3a(1*H*)-carboxylic acid (**74**) (top) and synthetic 4-(2,4-dioxo-1,4-dihydroquinazolin-3(2*H*)-yl)butanal (**76**) (bottom).

**7. Supplementary References**

1. Rosenau C. P., Jelier B. J., Gossert A. D. & Togni A. Exposing the Origins of Irreproducibility in Fluorine NMR Spectroscopy. *Angew. Chem. Int. Ed.* **57**, 9528–9533 (2018).

2. Einsiedler M., Jamieson C. S., Maskeri M. A., Houk K. N. & Gulder T. A. M. Fungal Dioxygenase AsqJ Is Promiscuous and Bimodal: Substrate-Directed Formation of Quinolones versus Quinazolinones. *Angew. Chem. Int. Ed.* **60**, 8297–8302 (2021).

3. Bräuer A., Beck P., Hintermann L. & Groll M. Structure of the dioxygenase AsqJ: mechanistic insights into a one-pot multistep quinolone antibiotic biosynthesis. *Angew. Chem. Int. Ed.* **55**, 422–426 (2016).

4. Blaskovich M. A. & Kahn M. Mild Conditions for Oxazolidin-5-one Formation. *Synthesis* **30**, 379–380 (1998).

5. Biron E., Chatterjee J. & Kessler H. Optimized selective *N*-methylation of peptides on solid support. *J. Pept. Sci.* **12**, 213–219 (2006).

6. Poterała M. & Plenkiewicz J. Synthesis of new chiral ionic liquids from α-hydroxycarboxylic acids. *Tetrahedron: Asymmetry* **22**, 294–299 (2011).

7. Sarie J. C., Thiehoff C., Neufeld J., Daniliuc C. G. & Gilmour R. Enantioselective Synthesis of 3-Fluorochromanes via Iodine(I)/Iodine(III) Catalysis. *Angew. Chem. Int. Ed.* **59**, 15069–15075 (2020).

8. Breault G., Eyermann C. J., Geng B., Morningstar M. & Reck F., inventors; AstraZeneca plc., assignee. Compounds for the Treatment of Multi-Drug Resistant Bacterial Infections patent WO2006134378 A1. 2006.

9. Houlden C. E.*, et al.* Room-Temperature Palladium-Catalyzed CH Activation: ortho-Carbonylation of Aniline Derivatives. *Angew. Chem. Int. Ed.* **48**, 1830–1833 (2009).

10. Fang J. & Zhou J. Efficient syntheses of 2,3-disubstituted natural quinazolinones via iridium catalysis. *Org. Biomol. Chem.* **10**, 2389–2391 (2012).

11. Afanasyev O. I.*, et al.* Redox Condensations of o-Nitrobenzaldehydes with Amines under Mild Conditions: Total Synthesis of the Vasicinone Family. *J. Org. Chem.* **85**, 9347–9360 (2020).

12. Liao H.-J.*, et al.* Insights into the Desaturation of Cyclopeptin and its C3 Epimer Catalyzed by a non-Heme Iron Enzyme: Structural Characterization and Mechanism Elucidation. *Angew. Chem. Int. Ed.* **57**, 1831–1835 (2018).

13. Chang W.-c., Li J., Lee J. L., Cronican A. A. & Guo Y. Mechanistic Investigation of a Non-Heme Iron Enzyme Catalyzed Epoxidation in (−)-4′-Methoxycyclopenin Biosynthesis. *J. Am. Chem. Soc.* **138**, 10390–10393 (2016).

14. Huang J.-L.*, et al.* Mechanistic Investigation of Oxidative Decarboxylation Catalyzed by Two Iron(II)- and 2-Oxoglutarate-Dependent Enzymes. *Biochemistry* **57**, 1838–1841 (2018).
